# Vps34-orchestrated lipid signaling processes regulate the transitional heterogeneity and functional adaptation of effector regulatory T cells

**DOI:** 10.1371/journal.pbio.3003074

**Published:** 2025-04-11

**Authors:** Erienne G. Norton, Nicole M. Chapman, Hao Shi, Xiaoxi Meng, Hongling Huang, Anil KC, Sherri Rankin, Jordy Saravia, Sujing Yuan, Haoran Hu, Peter Vogel, Hongbo Chi

**Affiliations:** 1 Department of Immunology, St. Jude Children’s Research Hospital, Memphis, Tennessee, United States of America; 2 St. Jude Graduate School of Biomedical Sciences, St. Jude Children’s Research Hospital, Memphis, Tennessee, United States of America; 3 Department of Pathology, St. Jude Children’s Research Hospital, Memphis, Tennessee, United States of America; National Cancer Institute, UNITED STATES OF AMERICA

## Abstract

Regulatory T cell (Treg) heterogeneity exists in lymphoid and non-lymphoid tissues, but we have limited understanding of context-dependent functions and spatiotemporal regulators of heterogenous Treg states, especially during perinatal life when immune tolerance is established. Here, we revealed that the class III PI3K Vps34 orchestrates effector Treg (eTreg) transitional heterogeneity during perinatal life. We found that loss of Vps34 reduced terminal eTreg accumulation in lymphoid tissues, associated with decreased Treg generation in non-lymphoid tissues and development of an early-onset autoimmune-like disease. After perinatal life, Vps34-deficient eTreg accumulation was further impaired due to reduced cell survival, highlighting temporal regulation of eTreg heterogeneity and maintenance by Vps34. Accordingly, inhibition of Vps34 in mature Tregs disrupted immune homeostasis but boosted anti-tumor immunity. Mechanistically, multiomics profiling approaches uncovered that Vps34-orchestrated transcriptional and epigenetic remodeling promotes terminal eTreg programming. Further, via genetic deletion of the Vps34-interacting proteins Atg14 or Uvrag in Tregs, we established that Atg14 but not Uvrag was required for the overall survival, but not terminal differentiation, of eTregs, suggesting that autophagy but not endocytosis partly contributed to Vps34-dependent effects. Accordingly, mice with Treg-specific loss of Atg14, but not Uvrag, had moderately disrupted immune homeostasis and reduced tumor growth, with Vps34- or Atg14-dependent gene signatures also being elevated in intratumoral Tregs from human cancer patients. Collectively, our study reveals distinct Vps34-orchestrated signaling events that regulate eTreg heterogeneity and functional adaptation and the pathophysiological consequences on autoimmunity versus anti-tumor immunity.

## Abstract 2

Regulatory T cells (Tregs) are heterogeneous and maintain immune tolerance, thereby preventing autoimmune disease and hindering anti-tumor immunity. This study shows that Vps34 and Atg14 regulate signaling events that control effector Treg heterogeneity and functional adaptation, with pathophysiological consequences on autoimmunity and anti-tumor immunity.

## Introduction

Regulatory T cells (Tregs) are essential for the establishment of immune tolerance, as autoimmune pathologies and disrupted tissue homeostasis occur under conditions of Treg depletion or dysfunction [1,2]. In contrast, the immunosuppressive function of Tregs is deleterious for effector T cell responses in the tumor microenvironment (TME) [3], but we have limited understanding of how Tregs are programmed to impart their context-dependent functions (e.g., establishment of immune tolerance versus immune suppression in tumors). This knowledge gap remains a barrier for rationally designing Treg-based therapies for autoimmunity, cancer, and other inflammatory diseases. Treg function and tissue localization are linked to their activation state [1,2]. Specifically, central Tregs (cTregs) predominantly localize to lymphoid tissues [4] and can undergo activation and differentiation into highly suppressive effector Tregs (eTregs). These activated eTregs localize to lymphoid tissues and represent the major Treg population in non-lymphoid and tumor tissues [5–7]. Further, Tregs with eTreg-like properties develop during perinatal life to establish immune tolerance [8] and promote tolerance in non-lymphoid tissues [9,10]. Beyond this cTreg versus eTreg paradigm, additional heterogeneous Treg states are emerging in both lymphoid and non-lymphoid tissues [7,11–15]. Whether Treg heterogeneity or its alteration shapes the context-dependent function of Tregs is poorly understood, and the mechanisms regulating Treg heterogeneity, especially beyond transcription factors [11,13,14,16], remain to be fully established.

Metabolic rewiring and adaptation orchestrate immune cell fate and function [17], including the activation, differentiation, and suppressive activity of Tregs [18–21]. Of particular interest, lipid metabolism is upregulated in Treg populations and supports Treg function under homeostasis and in the TME [21–23]. Beyond lipid-associated metabolic processes (e.g., biomass accumulation or bioenergetics), lipid-dependent signaling also shapes Treg activation and functional specialization. For example, lipid-dependent post-translational modifications (specifically protein farnesylation and geranylgeranylation) contribute to the generation and maintenance of eTregs and support Treg function [24]. In contrast, deletion of the lipid phosphatase PTEN in Tregs leads to the hyperactivation of selective effector CD4^ +^ T cell responses but also unleashes anti-tumor immune responses [25–27], suggesting that phospholipid signaling may fine-tune Treg function in diverse contexts. However, whether phospholipid signaling impacts Treg functional heterogeneity and adaptation remains elusive.

Phosphatidylinositol-3-kinases (PI3Ks) are key regulators of phospholipid signaling and are subdivided into distinct classes that generate specific phospholipid products critical for coordinating signaling events [28,29]. Among PI3Ks, the class III PI3K Vps34 orchestrates diverse cellular processes, including autophagy, endocytosis, and endocytic trafficking [28,30]. Vps34-dependent signaling events are dictated by the interaction of Vps34 with distinct proteins, including Atg14, which is important for initiating autophagy (i.e., Vps34 complex I), and Uvrag, which supports the pro-endocytic functions of Vps34 (i.e., Vps34 complex II) [28,30]. Previous studies have shown that Vps34 orchestrates the survival and metabolic fitness of different T cell populations via regulating either autophagy or endocytosis [31–35]. However, it remains unknown whether Vps34 complexes I and/or II control Treg heterogeneity or functional adaptation for the establishment of immune tolerance or suppression of anti-tumor immune responses.

Here, we show that distinct Vps34-dependent signaling processes shape the transitional heterogeneity and functional adaptation of eTregs in several diverse contexts, including perinatal non-lymphoid tissues. We found that perinatal mice with Treg-specific loss of Vps34 had reduced accumulation of Tregs in non-lymphoid tissues that corresponded with a cell-intrinsic survival defect of eTregs. Accordingly, constitutive loss of Vps34 specifically in Tregs caused mice to develop an early-onset, type-I-dominant, *Scurfy*-like inflammatory disease. Further, tamoxifen-inducible ablation of Vps34 in adult mice impaired eTreg survival and disrupted immune homeostasis, suggesting that continuous expression of Vps34 is required to mediate Treg accumulation in lymphoid and non-lymphoid tissues after perinatal life. Using single-cell transcriptomics profiling and experimental validations, we further established that Vps34 is essential for orchestrating eTreg transitional heterogeneity during perinatal life. Specifically, terminal eTregs, marked by high expression of KLRG1, IL-33 receptor ST2, and PD-1, were underrepresented among Vps34-deficient eTregs in lymphoid and especially non-lymphoid tissues of perinatal mice, with such effects being cell-intrinsic. Mechanistically, multiomics profiling approaches, including bulk ATAC-seq and single-cell transcriptomics paired with single-cell ATAC-seq, uncovered that Vps34 orchestrated transcriptional and epigenetic remodeling to promote terminal eTreg and non-lymphoid tissue Treg programming during perinatal life. To establish the specific Vps34 complex that orchestrates eTreg accumulation and transitional heterogeneity, we generated mice with Treg-specific loss of the Vps34-interacting protein Atg14 or Uvrag. Mice with Atg14-deficient but not Uvrag-deficient Tregs displayed mildly disrupted immune homeostasis that was associated with reduced eTreg survival but without alterations in terminal eTreg generation, thereby establishing the importance of autophagy (but not endocytosis) in dictating eTreg maintenance. Finally, we found that targeting Vps34 or Atg14, but not Uvrag, in Tregs reduced tumor growth. Accordingly, Vps34- or Atg14-dependent gene signatures were elevated in intratumoral Tregs from human cancer patients. Collectively, our study establishes distinct phospholipid signaling events that regulate eTreg heterogeneity and functional adaptation, which constitute key mechanisms orchestrating context-dependent Treg function.

### Vps34 supports Treg maintenance in lymphoid and non-lymphoid tissues

**Fig 1:**
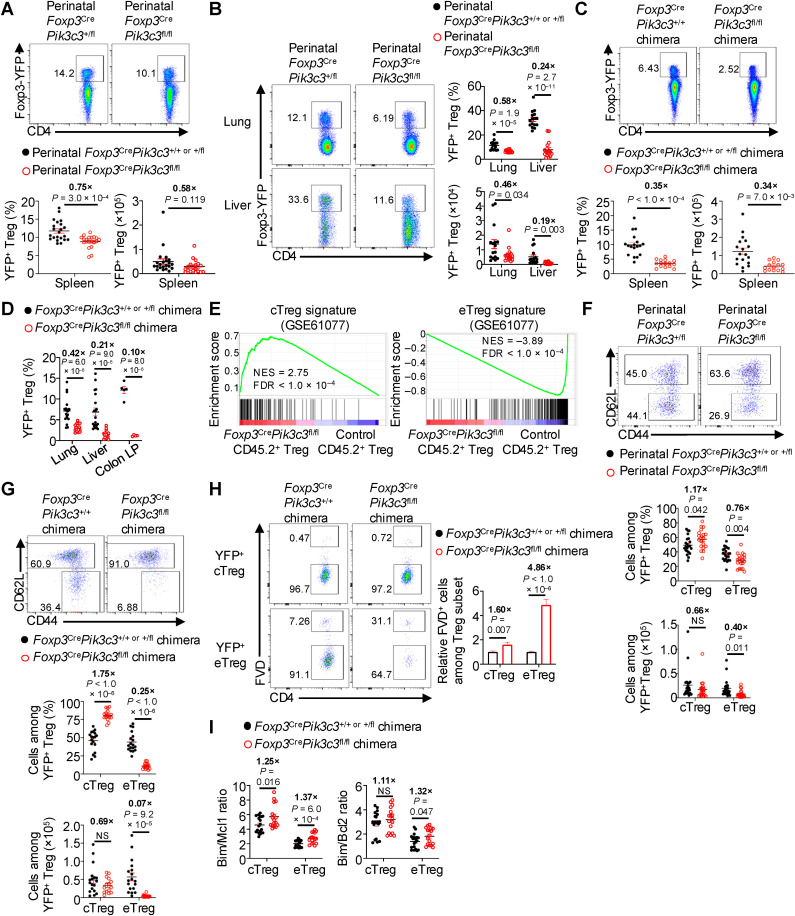
Vps34 supports Treg maintenance in lymphoid and non-lymphoid tissues. (**A**) Flow cytometry analysis (upper) and quantification (lower) of frequency and number of total TCRβ^+^CD4^+ ^Foxp3-YFP^+^ Tregs derived from the spleen of 7- to 11-day-old perinatal control (*n* = 22) or *Foxp3*^Cre^*Pik3c3*^fl/fl^ (*n* = 19) mice. (**B**) Flow cytometry analysis (left) and quantification (right) of frequencies and numbers of total TCRβ^+^CD4^+ ^Foxp3-YFP^+^ Tregs derived from the lung or liver of 7- to 11-day-old perinatal control or *Foxp3*^Cre^*Pik3c3*^fl/fl^ mice (*n* = 16 per group for both tissues). (**C**, **D**) Mixed BM chimera mice were generated by adoptive transfer of a 1:1 mixture of BM from CD45.2^+^ control or *Foxp3*^Cre^*Pik3c3*^fl/fl^ mice and CD45.1^+^ mice into sublethally irradiated *Rag1*^−/−^ mice. Flow cytometry analysis (upper) and quantification (lower) of frequencies and numbers of total TCRβ^+^CD4^+ ^Foxp3-YFP^+^ Tregs derived from the spleen of control (*n* = 18) or *Foxp3*^Cre^*Pik3c3*^fl/fl^ (*n* = 15) mixed BM chimera mice (**C**). Quantification of frequency of total TCRβ^+^CD4^+ ^Foxp3-YFP^+^ Tregs derived from the lung, liver, or colon LP of control (*n* = 19 for lung, 20 for liver, and 5 for colon LP) or *Foxp3*^Cre^*Pik3c3*^fl/fl^ (*n* = 16 for lung, 17 for liver, and 4 for colon LP) mixed BM chimera mice (**D**). BM, bone marrow; LP, lamina propria. (**E**) CD4^+ ^Foxp3-YFP^+^ Tregs (CD45.2^+^) were sort-purified from the spleen of control (*n* = 4) or *Foxp3*^Cre^*Pik3c3*^fl/fl^ (*n* = 5) mixed BM chimera mice and profiled by microarray analysis (see Materials and methods for details). GSEA enrichment plots showing increased cTreg signature (left; i.e., top 200 upregulated genes [log_2_FC > 0.5, FDR < 0.05] in cTregs versus eTregs) and decreased eTreg signature (right; i.e., top 200 upregulated genes [log_2_FC > 0.5, FDR < 0.05] in eTregs versus cTregs) in CD45.2^+^ Tregs from *Foxp3*^Cre^*Pik3c3*^fl/fl^ mixed BM chimera mice compared to control mixed BM chimera mice. Signatures were generated using GSE61077 [5]. GSEA, gene set enrichment analysis; NES, normalized enrichment score; FDR, false discovery rate. (**F**) Flow cytometry analysis (upper) and quantification (lower) of frequencies and numbers of TCRβ^+^CD4^+ ^Foxp3-YFP^+^CD44^low^CD62L^high^ cTregs or TCRβ^+^CD4^+ ^Foxp3-YFP^+^CD44^high^CD62L^low^ eTregs derived from the spleen of 7- to 11-day-old perinatal control (*n* = 22) or *Foxp3*^Cre^*Pik3c3*^fl/fl^ (*n* = 19) mice. (**G**) Flow cytometry analysis (upper) and quantification (lower) of frequencies and numbers of TCRβ^+^CD4^+ ^Foxp3-YFP^+^CD44^low^CD62L^high^ cTregs or TCRβ^+^CD4^+ ^Foxp3-YFP^+^CD44^high^CD62L^low^ eTregs derived from the spleen of control (*n* = 18) or *Foxp3*^Cre^*Pik3c3*^fl/fl^ (*n* = 15) mixed BM chimera mice. (**H**) Flow cytometry analysis (left) and quantification (right) of relative (normalized to average control in each experiment) frequencies of FVD^+^ non-viable TCRβ^+^CD4^+ ^Foxp3^+ ^CD44^low^CD62L^high^ cTregs or TCRβ^+^CD4^+ ^Foxp3^+ ^CD44^high^CD62L^low^ eTregs (all pre-gated on CD45.2^+^ cells) derived from the spleen of control (*n* = 22) or *Foxp3*^Cre^*Pik3c3*^fl/fl^ (*n* = 21) mixed BM chimera mice. FVD, fixable viability dye. (**I**) Quantification of Bim/Mcl1 (left) and Bim/Bcl2 (right) ratios (based on gMFIs) from TCRβ^+^CD4^+ ^Foxp3^+ ^CD44^low^CD62L^high^ cTregs or TCRβ^+^CD4^+ ^Foxp3^+ ^CD44^high^CD62L^low^ eTregs derived from the spleen of control (*n* = 19) or *Foxp3*^Cre^*Pik3c3*^fl/fl^ (*n* = 17) mixed BM chimera mice. gMFI, geometric mean fluorescence intensity. Data are shown as mean ± s.e.m. (**A**–**D**, **F**–**I**). Relative (normalized to average control in each experiment) fold change comparisons between genotypes in each tissue (**B**, **D**) or cell type (**A**, **C**, **F**–**I**) are shown in bold. Two-tailed Student *t* test (**A**–**D**, **F**–**I**) or Benjamini–Hochberg test (**E**); NS, not significant. Data are compiled from 15 (**A**, **F**), 12 (**B**), 5 (**C**, **G**, **I**), 6 (**D**, lung and liver; **H**), or 2 (**D**, colon LP) independent experiments. Numbers in plots indicate percentages of cells in gates (**A**–**C**, **F**–**H**). The numerical data underlying the graphs shown in this figure are found in S1 Data (**A**–**C**, **F**–**H**). Raw microarray data used for analysis in (**E**) have been deposited to GEO SuperSeries access number GSE279606.

To establish a functional role for Vps34 in Tregs, we bred mice bearing the floxed alleles of *Pik3c3* (encoding for Vps34; *Pik3c3*^fl/fl^) [36] with *Foxp3*^YFP-Cre^ mice [37] to generate *Foxp3*^YFP-Cre^*Pik3c3*^fl/fl^ mice (hereafter called *Foxp3*^Cre^*Pik3c3*^fl/fl^ mice). *Pik3c3* was efficiently deleted in Tregs from *Foxp3*^Cre^*Pik3c3*^fl/fl^ mice compared to control (*Foxp3*^Cre^*Pik3c3*^+ / +^ or *Foxp3*^Cre^*Pik3c3*^+ /fl^ mice; denoted as *Foxp3*^Cre^*Pik3c3*^+ / + or + /fl^) mice, as determined by quantitative PCR analysis (S1A Fig). Flow cytometry analysis revealed a reduced frequency, albeit not number, of Tregs in the spleen of perinatal (i.e., 7–11 days after birth) *Foxp3*^Cre^*Pik3c3*^fl/fl^ mice compared to littermate control mice (Fig 1A), with such effects also being evident after perinatal life (i.e., ≥3 weeks after birth) (S1B Fig). There was a more pronounced reduction of Treg cellularity in the lung and liver (40%–50% and ~80% decrease in cell number, respectively) of perinatal *Foxp3*^Cre^*Pik3c3*^fl/fl^ mice compared to littermate control mice (Fig 1B), indicating that Vps34 supports Treg accumulation, especially in non-lymphoid tissues, during perinatal life.

To examine cell-intrinsic effects associated with loss of Vps34 in Tregs, we used two complementary systems. First, we generated mixed bone marrow (BM) chimeras by mixing BM from CD45.2^ +^ control *Foxp3*^Cre^*Pik3c3*^+ /+ or +/fl^ or *Foxp3*^Cre^*Pik3c3*^fl/fl^ mice with “spike” BM from control CD45.1^ +^ mice at a 1:1 ratio, followed by adoptive transfer into sublethally irradiated *Rag1*^−/−^ mice (hereafter called control or *Foxp3*^Cre^*Pik3c3*^fl/fl^ mixed BM chimeras). The CD45.1^ +^ BM-derived Tregs circumvent inflammation that may occur in mice with reduced Treg accumulation, thereby allowing for analysis of cell-intrinsic effects [21,24,38]. Flow cytometry analysis revealed significantly reduced Tregs in the spleen of *Foxp3*^Cre^*Pik3c3*^fl/fl^ mixed BM chimeras compared to control counterparts (Fig 1C). Further, Treg frequencies were reduced in non-lymphoid tissues (i.e., lung, liver, and colon lamina propria [LP]) of *Foxp3*^Cre^*Pik3c3*^fl/fl^ compared to control mixed BM chimeras (Fig 1D). Second, we used *Foxp3*^Cre/+ ^*Pik3c3*^fl/fl^ mosaic female mice that contain ~50% *Foxp3*^Cre^-expressing Tregs (due to random inactivation of the X-chromosome that contains *Foxp3*) to mediate deletion of *Pik3c3* and ~50% wild-type Tregs that can circumvent inflammation [24]. Similar to mixed BM chimeras, Tregs were reduced in the spleen and lung of *Foxp3*^Cre/+ ^*Pik3c3*^fl/fl^ mosaic mice compared to control *Foxp3*^Cre/+ ^*Pik3c3*^+/+ or +/fl^ mosaic mice (S1C and S1D Fig). Thus, Vps34 plays a cell-intrinsic role in driving Treg accumulation in lymphoid and non-lymphoid tissues.

To unbiasedly establish the cell-intrinsic mechanisms by which Vps34 regulates Treg fitness, we performed transcriptome profiling on total Tregs isolated from control or *Foxp3*^Cre^*Pik3c3*^fl/fl^ mixed BM chimeras via microarray analysis. Differential expression analysis revealed that Vps34-deficient Tregs had marked transcriptional alterations compared to control counterparts, including altered expression of genes associated with Treg suppressive function (e.g., *Icos*, *Il10*, *Klrg1*) (S1E Fig and S1 Table). Further, gene set enrichment analysis (GSEA) revealed that Vps34-deficient Tregs had upregulated cTreg and downregulated eTreg gene signatures [5] (Fig 1E), suggesting altered proportions of cTregs and eTregs in the absence of Vps34. Indeed, flow cytometry analysis revealed a significant reduction in the frequency and number of eTregs in perinatal *Foxp3*^Cre^*Pik3c3*^fl/fl^ mice with a corresponding increase in the frequency (albeit not number) of cTregs (Fig 1F), and these effects on eTreg accumulation were maintained after perinatal life (S1F Fig). Moreover, eTregs were reduced in *Foxp3*^Cre^*Pik3c3*^fl/fl^ mixed BM chimeras (Fig 1G) and *Foxp3*^Cre/+ ^*Pik3c3*^fl/fl^ mosaic mice (S1G Fig). These results indicate that Vps34 has a cell-intrinsic role in supporting eTreg accumulation in vivo.

We next explored the possible mechanisms underlying defective Vps34-deficient eTreg accumulation using the abovementioned cell-intrinsic systems. First, we examined whether Treg stability was altered by examining Foxp3 and CD25 expression, as their downregulation can mark “unstable” or “ex” Tregs [39–42]. In mixed BM chimeras, we found that Vps34-deficient eTregs (and cTregs) showed comparable or increased expression of Foxp3 and CD25 (S1H Fig), suggesting that Vps34 does not affect the stability of Treg populations in vivo. Second, we performed Ki67 staining of Treg populations, which revealed no significant differences in the proportions of Ki67^ +^ cells among total Tregs, cTregs, or eTregs in mixed BM chimeras (S1I Fig), indicating that the observed decreases in Treg accumulation across tissues are unlikely due to a proliferation defect. Third, as TCR-dependent signals are important for generating and maintaining eTregs [5,6], we sort-purified CD44^low^CD62L^high^ cTregs from control or *Foxp3*^Cre/+ ^*Pik3c3*^fl/fl^ mosaic mice and stimulated them in vitro with anti-CD3 and anti-CD28 antibodies plus IL-2 for 72 h to promote eTreg-like cell generation [24,43]. We found that Vps34-deficient cTregs differentiated into CD44^high^CD62L^low^ eTreg-like cells in vitro (S1J Fig), suggesting that these cells can undergo activation in response to TCR stimulation.

Fourth, we explored whether cell death contributes to the loss of eTregs and found a marked increase in the frequencies of fixable viability dye-permeable (FVD)^ +^ cells that marked non-viable eTregs from both *Foxp3*^Cre^*Pik3c3*^fl/fl^ mixed BM chimeras and *Foxp3*^Cre/+ ^*Pik3c3*^fl/fl^ mosaic mice as compared to their respective control counterparts (Figs 1H and S1K), indicative of increased eTreg death in the absence of Vps34. Moreover, flow cytometry staining for pro-apoptotic protein Bim and anti-apoptotic proteins Mcl1 and Bcl2 (of note, Mcl1 plays a more dominant role in promoting Treg survival than Bcl2 [44]) revealed that Bim/Mcl-1 and Bim/Bcl2 ratios (indicative of the relative balance of pro-apoptotic versus anti-apoptotic proteins [45]) were both increased in Vps34-deficient eTregs from *Foxp3*^Cre^*Pik3c3*^fl/fl^ mixed BM chimeras compared to control counterparts (Fig 1I), further supporting the notion that Vps34 deficiency promotes eTreg death. There were also elevated FVD^ +^ percentage and Bim/Mcl1 ratio in Vps34-deficient versus control cTregs, albeit with more modest effects compared to the eTreg compartment (Figs 1H, 1I, and S1K). Collectively, these results establish that Vps34 is important for eTreg (and to a lesser extent cTreg) survival, which likely contributes to the reduced accumulation of Tregs in lymphoid and non-lymphoid tissues.

### Vps34 orchestrates Treg function in vivo

**Fig 2:**
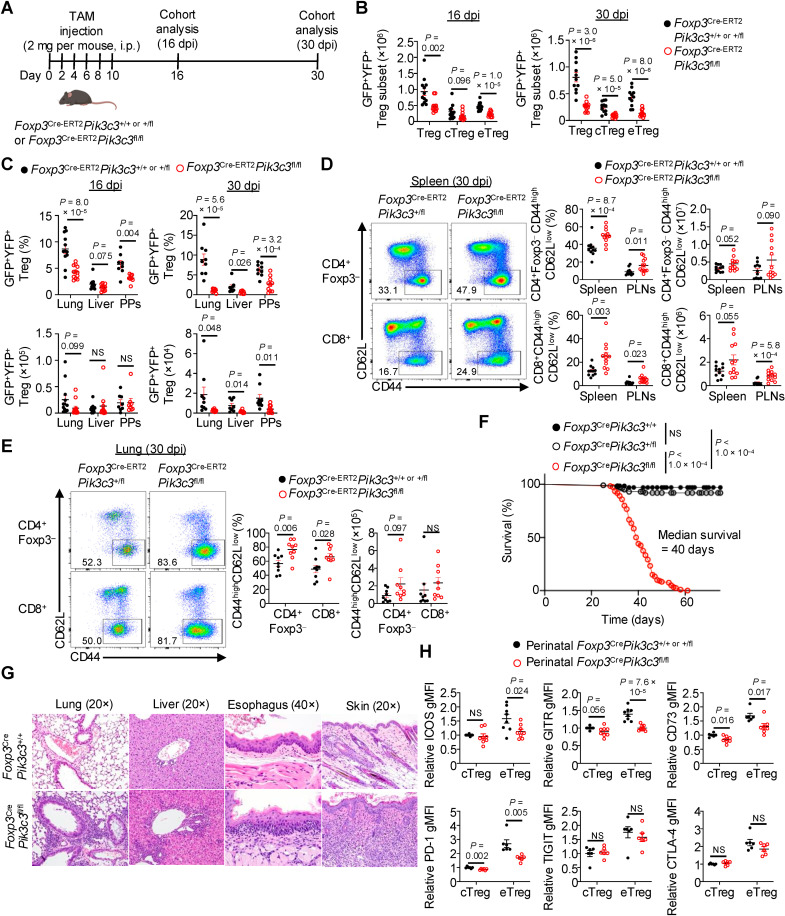
Vps34 orchestrates Treg function in vivo. (**A**) Experimental schematic for temporal analysis of mature Tregs upon TAM-inducible deletion of *Pik3c3*. Control or *Foxp3*^Cre-ERT2^*Pik3c3*^fl/fl^ mice were given i.p. injections of TAM (2 mg/mouse dissolved in corn oil) every other day for a total of 6 injections. Mice were analyzed at 16 or 30 days after the first TAM injection (16 and 30 dpi, respectively). i.p., intraperitoneal; TAM, tamoxifen; dpi, days post-injection. Mouse image created with BioRender. (**B**) Quantification of numbers of total GFP^+^YFP^+^ Tregs, GFP^+^YFP^+^CD44^low^CD62L^high^ cTregs, or GFP^+^YFP^+^CD44^high^CD62L^low^ eTregs among TCRβ^+^CD4^+^ cells derived from the spleen of control (*n* = 12 for 16 dpi and 11 for 30 dpi) or *Foxp3*^Cre-ERT2^*Pik3c3*^fl/fl^ (*n* = 12 for 16 dpi and 12 for 30 dpi) mice at 16 (left) and 30 (right) dpi. (**C**) Quantification of frequencies (upper) and numbers (lower) of total GFP^+^YFP^+^ Tregs among TCRβ^+^CD4^+^ cells derived from the lung, liver, or PPs of control (*n* = 12 for 16 dpi lung, 12 for 16 dpi liver, 8 for 16 dpi PPs, 9 for 30 dpi lung, 8 for 30 dpi liver, and 9 for 30 dpi PPs) or *Foxp3*^Cre-ERT2^*Pik3c3*^fl/fl^ (*n* = 12 for 16 dpi lung, 11 for 16 dpi liver, 8 for 16 dpi PPs, 9 for 30 dpi lung, 8 for 30 dpi liver, and 10 for 30 dpi PPs) mice at 16 (left) and 30 (right) dpi. PPs, Peyer’s patches. (**D**) Flow cytometry analysis (left) and quantification (right) of frequencies and numbers of CD44^high^CD62L^low^ effector/memory cells among TCRβ^+^CD4^+ ^GFP^−^YFP^−^ (denoted as CD4^+ ^Foxp3^−^) and TCRβ^+^CD8^+^ T cells derived from the spleen or PLNs of control (*n* = 11 for both tissues) or *Foxp3*^Cre-ERT2^*Pik3c3*^fl/fl^ (*n* = 12 for both tissues) mice at 30 dpi. PLNs, peripheral lymph nodes. (**E**) Flow cytometry analysis (left) and quantification (right) of frequencies and numbers of CD44^high^CD62L^low^ effector/memory cells among TCRβ^+^CD4^+ ^GFP^−^YFP^−^ (denoted as CD4^+ ^Foxp3^−^) and TCRβ^+^CD8^+^ T cells derived from the lung of control or *Foxp3*^Cre-ERT2^*Pik3c3*^fl/fl^ mice at 30 dpi (*n* = 9 per group). (**F**) Survival analysis of *Foxp3*^Cre^*Pik3c3*^+/+^ (*n* = 70), *Foxp3*^Cre^*Pik3c3*^+ /fl^ (*n* = 102), and *Foxp3*^Cre^*Pik3c3*^fl/fl^ (*n* = 120) mice, with the median survival of *Foxp3*^Cre^*Pik3c3*^fl/fl^ mice indicated. (**G**) Hematoxylin and eosin staining of lung, liver, esophagus, and skin from 30-day-old control or *Foxp3*^Cre^*Pik3c3*^fl/fl^ mice. (**H**) Quantification of relative (normalized to average control in each experiment) gMFIs of ICOS (*n* = 8 per group), GITR (*n* = 8 per group), CD73 (*n* = 6 for control and 7 for *Foxp3*^Cre^*Pik3c3*^fl/fl^), PD-1 (*n* = 6 per group), TIGIT (*n* = 6 per group), and CTLA-4 (*n* = 6 per group) in CD44^low^CD62L^high^ cTregs or CD44^high^CD62L^low^ eTregs among total TCRβ^+^CD4^+ ^Foxp3-YFP^+^ (ICOS, GITR, CD73, PD-1) or TCRβ^+^CD4^+ ^Foxp3^+^ (TIGIT, CTLA-4) Tregs derived from the spleen of 7- to 11-day-old perinatal control or *Foxp3*^Cre^*Pik3c3*^fl/fl^ mice, as determined by flow cytometry analysis. gMFI, geometric mean fluorescence intensity. Data are shown as mean ± s.e.m. (**B**–**E**, **H**). Two-tailed Student *t* test (**B**–**E**, **H**) or Mantel–Cox test (**F**); NS, not significant. Data are compiled from 5 (**B**, **C**, lung and liver; **D**, **E**), or ≥3 (**C**, PPs; **H**) independent experiments or are representative of 2 biological replicates (**G**). Numbers in plots indicate percentages of cells in gates (**D**, **E**). The numerical data underlying the graphs shown in this figure are found in S2 Data (**B**–**F**, **H**).

To determine whether Vps34 orchestrates the maintenance and function of mature Tregs after perinatal life [46], we bred *Pik3c3*^fl/fl^ mice with *Foxp3*^Cre-ERT2-GFP^*Rosa26*-YFP^fl/fl^ mice to generate *Foxp3*^Cre-ERT2^*Pik3c3*^fl/fl^ mice. Upon tamoxifen treatment, Cre-ERT2 mediates deletion of *Pik3c3* specifically in mature Tregs as marked by co-expression of GFP and YFP [22,38,46]. Specifically, we intraperitoneally injected control *Foxp3*^Cre-ERT2^*Pik3c3*^+ /+ or + /fl^ or *Foxp3*^Cre-ERT2^*Pik3c3*^fl/fl^ mice with tamoxifen on days 0, 2, 4, 6, 8, and 10 for a total of six injections [38], followed by analysis of mice at day 16 or 30 after the first tamoxifen injection (called 16 dpi and 30 dpi, respectively) (Fig 2A). *Pik3c3* expression was decreased in sort-purified GFP^+^YFP^ +^ Tregs from *Foxp3*^Cre-ERT2^*Pik3c3*^fl/fl^ mice compared to control counterparts at 16 dpi (S2A Fig). We observed marked reductions in the numbers of total GFP^+^YFP^ +^ Tregs in the spleen at both the early (16 dpi) and late (30 dpi) timepoints after tamoxifen injection in *Foxp3*^Cre-ERT2^*Pik3c3*^fl/fl^ mice compared to control counterparts, as well as reductions of eTregs at both timepoints (Fig 2B). Treg accumulation was also markedly reduced in non-lymphoid tissues (i.e., lung, liver, and Peyer’s patches [PPs]) of tamoxifen-treated *Foxp3*^Cre-ERT2^*Pik3c3*^fl/fl^ mice compared to control mice (Fig 2C). Thus, Vps34 is important for the maintenance of mature Tregs, especially the activated eTreg population, in both lymphoid and non-lymphoid tissues.

Reduced eTreg accumulation is often associated with disrupted immune tolerance and spurious T cell activation [1,2,5,7]. At 30 dpi, we found that CD44^high^CD62L^low^ effector/memory CD4^ +^ and CD8^ +^ T cells had accumulated in the spleen and peripheral lymph nodes (PLNs) of tamoxifen-treated *Foxp3*^Cre-ERT2^*Pik3c3*^fl/fl^ mice compared to control mice (Fig 2D). The frequencies (albeit not numbers) of effector/memory CD4^ +^ and CD8^ +^ T cells were also elevated in the lung at 30 dpi (Fig 2E), suggesting that the loss of Vps34 in mature Tregs promotes dysregulated immune tolerance. Given these results, we next examined T cell homeostasis in ≥3-week-old *Foxp3*^Cre^*Pik3c3*^fl/fl^ mice. Similar to tamoxifen-treated *Foxp3*^Cre-ERT2^*Pik3c3*^fl/fl^ mice, there were increased effector/memory CD4^ +^ and CD8^ +^ T cells in *Foxp3*^Cre^*Pik3c3*^fl/fl^ mice compared to control mice (S2B Fig), indicative of disrupted T cell homeostasis. Next, we stimulated splenocytes in vitro with phorbol myristate acetate (PMA) and ionomycin and examined IL-4, IL-17A, and IFN-γ production by Foxp3^− ^CD4^+^ T cells and IFN-γ production by CD8^+^ T cells. There were marked elevations in IFN-γ-producing CD4^ +^ and CD8^ +^ T cells and more modest increases in CD4^+ ^IL-4^+ ^, but not CD4^+ ^IL-17A^+ ^, cells in *Foxp3*^Cre^*Pik3c3*^fl/fl^ mice compared to control mice (S2C Fig), suggesting that loss of Vps34 in Tregs triggers a type-I-dominant inflammatory disorder. These results indicate that Tregs require continuous Vps34 expression to maintain immune tolerance in vivo.

Given these results, we next examined whether mice bearing Vps34-deficient Tregs showed features of disrupted immune tolerance. As compared to either *Foxp3*^Cre^*Pik3c3*^+/+^ or *Foxp3*^Cre^*Pik3c3*^+/fl^ control mice, *Foxp3*^Cre^*Pik3c3*^fl/fl^ mice developed an inflammatory, *Scurfy*-like phenotype [47] including focal dermatitis on the ears and tail (S2D Fig). Further, *Foxp3*^Cre^*Pik3c3*^fl/fl^ mice had increased size and cellularity of both the spleen and PLNs (S2E Fig) and showed early-onset lethality compared to control counterparts, with a median survival of 40 days (Fig 2F). Histological analysis using hematoxylin and eosin (H&E) staining revealed increased inflammation and leukocytic infiltration in the lung, liver, esophagus, and skin of *Foxp3*^Cre^*Pik3c3*^fl/fl^ mice compared to control counterparts (Fig 2G), indicating that Vps34 deficiency in Tregs contributes to loss of tissue tolerance. Thus, ablation of Vps34 in Tregs leads to development of a *Scurfy*-like autoimmune disease, suggesting that Vps34-deficient Tregs have defective function in vivo.

Tregs that arise during perinatal life are essential for the establishment of immune and tissue tolerance [8–10]. As *Foxp3*^Cre^*Pik3c3*^fl/fl^ mice rapidly developed autoimmune-like inflammatory disease, we hypothesized that Vps34 may be important for perinatal Treg function. In line with this notion, effector/memory CD4^+ ^, but not CD8^+ ^, T cells were increased in perinatal *Foxp3*^Cre^*Pik3c3*^fl/fl^ mice compared to their littermate controls (S2F Fig). Perinatal *Foxp3*^Cre^*Pik3c3*^fl/fl^ mice also had trending increased proportions of IFN-γ-producing CD4^ +^ and CD8^ +^ T cells (S2G Fig), suggesting that Vps34 supports Treg suppressive function to promote establishment of immune and tissue tolerance during perinatal life. Further, several Treg suppressive markers, including ICOS, GITR, CD73, and PD-1 (but not TIGIT or CTLA-4), were reduced in splenic eTregs from perinatal *Foxp3*^Cre^*Pik3c3*^fl/fl^ mice compared to littermate controls, with no or more modest alterations observed in Vps34-deficient cTregs (Fig 2H). Largely similar results were observed for Vps34-deficient eTregs from *Foxp3*^Cre^*Pik3c3*^fl/fl^ mixed BM chimeras (S2H Fig), highlighting a cell-intrinsic effect. Thus, consistent with its role in supporting eTreg maintenance, Vps34 shapes Treg function in vivo and the expression of suppressive molecules on eTregs.

### Vps34 is essential for the terminal differentiation of eTregs during perinatal life

The above results suggested that Vps34 deficiency led to cell-intrinsic reductions in eTreg survival and function. Interestingly, unlike the Vps34-deficient Tregs isolated from mixed BM chimeras or mosaic mice (Figs 1H and S1K), the frequency of FVD^ +^ eTregs showed only a modest, trending increase in perinatal *Foxp3*^Cre^*Pik3c3*^fl/fl^ mice compared to littermate controls (S2I Fig). Further, we found that FVD^ +^ cTregs and eTregs accumulated at 30 dpi in tamoxifen-treated *Foxp3*^Cre-ERT2^*Pik3c3*^fl/fl^ mice compared to control mice but were not significantly altered at 16 dpi (S2J Fig). These results suggest that the increased cell death phenotype is unlikely to fully account for the defects in Vps34-deficient Tregs in establishing immune tolerance, in line with the notion that expression of suppressive molecules was reduced on eTregs from perinatal mice as described above.

**Fig 3:**
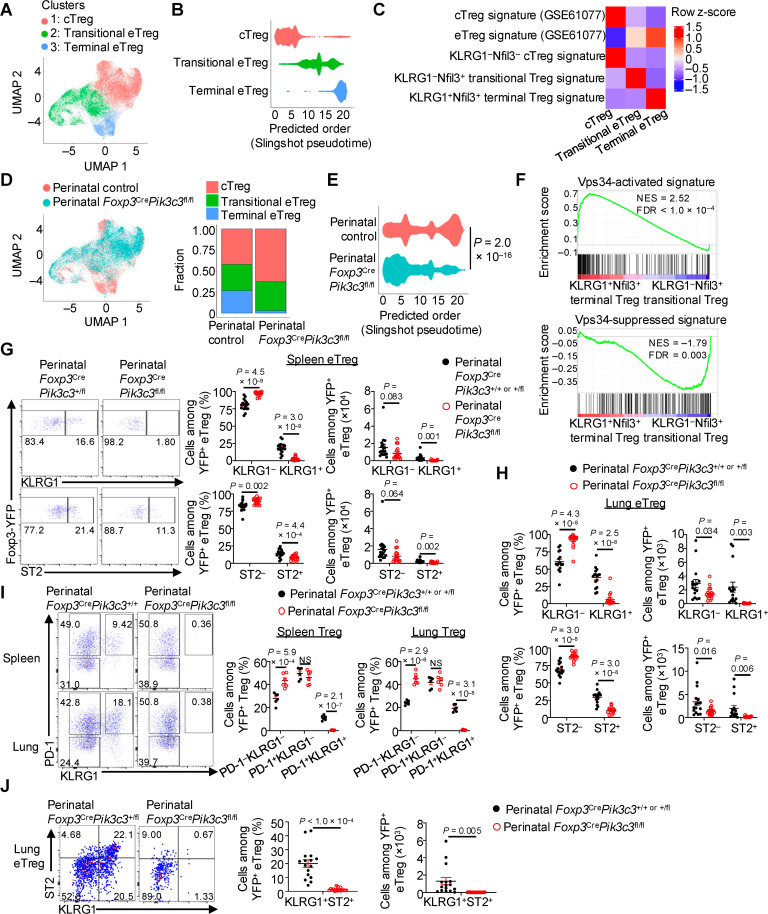
Vps34 is essential for terminal differentiation of eTregs during perinatal life. (**A**–**E**) TCRβ^+^CD4^+ ^Foxp3-YFP^ +^ Tregs were sort-purified from the spleen of 10- to 12-day-old perinatal control or *Foxp3*^Cre^*Pik3c3*^fl/fl^ mice and profiled by single-cell RNA-seq (scRNA-seq) or single-nuclear RNA-seq (snRNA-seq). scRNA-seq and snRNA-seq analyses of Tregs from perinatal control (*n* = 2) or *Foxp3*^Cre^*Pik3c3*^fl/fl^ (*n* = 3) mice were merged before performing subsequent analyses (see Materials and methods for details). UMAP plot depicting the clusters of three cell states (1: cTreg; 2: transitional eTreg, and 3: terminal eTreg) among Tregs from both perinatal control and perinatal *Foxp3*^Cre^*Pik3c3*^fl/fl^ mice. UMAP, uniform manifold approximation and projection (**A**). Slingshot pseudotime analysis showing the predicted differentiation order of cTregs, transitional eTregs, and terminal eTregs from perinatal control mice (**B**). Heatmap depicting the relative average expression of the indicated gene signatures (curated from public datasets [5,11]; see Materials and methods for details) in the indicated Treg states from single-cell transcriptome profiling (**C**). UMAP plot of Tregs derived from 10- to 12-day-old perinatal control and *Foxp3*^Cre^*Pik3c3*^fl/fl^ mice with genotypes color-coded (left). Quantification of the proportions of cTregs, transitional eTregs, and terminal eTregs in each genotype (right) (**D**). Predicted differentiation order of Tregs from perinatal control and *Foxp3*^Cre^*Pik3c3*^fl/fl^ mice based on Slingshot pseudotime analysis (**E**). (**F**) GSEA enrichment plots showing increased Vps34-activated signature (upper; i.e., top 200 downregulated genes [log_2_FC < −0.5, *P* < 0.05]) in Vps34-deficient Tregs versus control Tregs from mixed BM chimeras; see S2 Table and Materials and methods for details) and decreased Vps34-suppressed signature (lower; i.e., top 200 upregulated genes [log_2_FC > 0.5, *P* < 0.05]) in Vps34-deficient Tregs versus control Tregs from mixed BM chimeras; see S2 Table and Materials and methods for details) in KLRG1^+ ^Nfil3^+^ terminal Tregs compared to KLRG1^− ^Nfil3^+^ transitional Tregs (from a public RNA-seq dataset GSE130884 [11]; see Materials and methods for details). GSEA, gene set enrichment analysis; NES, normalized enrichment score; FDR, false discovery rate. (**G**) Flow cytometry analysis (left) and quantification (right) of the frequencies and numbers of KLRG1^−^ and KLRG1^+^ cells (upper) or ST2^−^ and ST2^+^ cells (lower) among TCRβ^+^CD4^+ ^Foxp3-YFP^+^CD44^high^CD62L^low^ eTregs derived from the spleen of 7- to 11-day-old perinatal control (*n* = 18) or *Foxp3*^Cre^*Pik3c3*^fl/fl^ (*n* = 16) mice. (**H**) Quantification of the frequencies and numbers of KLRG1^−^ and KLRG1^+^ (upper) or ST2^−^ and ST2^+^ (lower) populations among TCRβ^+^CD4^+ ^Foxp3-YFP^+^CD44^high^CD62L^low^ eTregs derived from the lung of 7- to 11-day-old perinatal control or *Foxp3*^Cre^*Pik3c3*^fl/fl^ mice (*n* = 16 per group). (**I**) Flow cytometry analysis (left) and quantification (right) of the frequencies of PD-1^− ^KLRG1^−^ cTregs, PD-1^+ ^KLRG1^−^ transitional Tregs, and PD-1^+ ^KLRG1^+^ terminal Tregs among total TCRβ^+^CD4^+ ^Foxp3-YFP^+^ Tregs derived from the spleen or lung of 7- to 11-day-old perinatal control or *Foxp3*^Cre^*Pik3c3*^fl/fl^ mice (*n* = 6 per group). (**J**) Flow cytometry analysis (left) and quantification (right) of the frequency and number of KLRG1^+ ^ST2^+^ cells among TCRβ^+^CD4^+ ^Foxp3-YFP^+^CD44^high^CD62L^low^ eTregs derived from the lung of 7- to 11-day-old perinatal control or *Foxp3*^Cre^*Pik3c3*^fl/fl^ mice (*n* = 16 per group). Data are shown as mean ± s.e.m. (**G**–**J**). Wilcoxon rank sum test (**E**), Benjamini–Hochberg test (**F**), or two-tailed Student *t* test (**G**–**J**); NS, not significan*t*. Data are compiled from 14 (**G**), 12 (**H**), 4 (**I**), or 11 (**J**) independent experiments. Numbers in plots indicate percentages of cells in gates (**G**, **I**, **J**). The numerical data underlying the graphs shown in this figure are found in S3 Data (**G**–**J**). Raw scRNA-seq and snRNA-seq or microarray data used for analyses in (**A**–**F**) have been deposited to GEO SuperSeries access number GSE279606.

To establish mechanisms by which Vps34 supports perinatal Treg fitness and functional adaptation, we performed single-cell transcriptome profiling using Tregs isolated from the spleen of perinatal control or *Foxp3*^Cre^*Pik3c3*^fl/fl^ mice (see Materials and methods for details). Uniform Manifold Approximation and Projection (UMAP) and clustering analyses revealed three unique clusters of Tregs in these perinatal mice (Fig 3A). Cluster 1 expressed high levels of cTreg-associated genes (*Sell*, *Tcf7*, and *Il2ra*) [4,48,49] and was therefore denoted as cTregs (Figs 3A, S3A, and S3B). Clusters 2 and 3 expressed progressively lower levels of these cTreg-associated genes but higher levels of the eTreg-associated gene *Cd44* [4], leading to their classification as eTregs (Figs 3A, S3A, and S3B). Compared to cluster 2, cluster 3 had high expression of *Klrg1* and *Il1rl1* (encodes for ST2), two genes that are enriched in terminal populations of Tregs [11,50–53], whereas cluster 2 had higher expression of the proliferation marker *Mki67* (Figs 3A, S3A, and S3B); thus, we classified cluster 2 as transitional eTregs and cluster 3 as terminal eTregs. Slingshot pseudotime analysis [54] predicted the stepwise maturation of perinatal Tregs from cTregs via transitional eTregs toward terminal eTregs (Fig 3B), highlighting the transitional heterogeneity of perinatal Tregs. Further, the cTreg signature [5] was highest in cTregs, whereas the eTreg signature [5] was enriched in transitional and terminal eTregs (Fig 3C). Signatures corresponding to KLRG1^−^Nfil3^−^ cTregs [11], KLRG1^−^Nfil3^ +^ transitional Tregs [11], and KLRG1^+ ^Nfil3^ +^ terminal Tregs [11] were enriched in the cTregs, transitional eTregs, and terminal eTregs, respectively (Fig 3C), further supporting the classifications of the perinatal Treg states. Collectively, these results identify Treg heterogeneity in the perinatal environment, including two discrete eTreg states.

Next, we determined the effects of Vps34 deficiency on Treg transitional heterogeneity during perinatal life. Single-cell transcriptome profiling revealed that Vps34-deficient Tregs from perinatal *Foxp3*^Cre^*Pik3c3*^fl/fl^ mice had an increased frequency of cTregs (Fig 3D), in line with the flow cytometry analysis described above (Fig 1F). Further, Vps34 deficiency led to a modest accumulation of transitional eTregs, whereas there was a striking decrease of the terminal eTreg population (Fig 3D). Moreover, pseudotime analysis inference revealed an impaired differentiation trajectory of perinatal Tregs lacking Vps34, as Vps34-deficient Tregs from perinatal *Foxp3*^Cre^*Pik3c3*^fl/fl^ mice accumulated earlier in pseudotime than control counterparts (Fig 3E). Thus, Vps34 is likely important for the terminal differentiation of perinatal Tregs. In support of this notion, we found that the Vps34-activated signature (derived from downregulated genes in Vps34-deficient versus control Tregs; see S2 Table and Materials and methods) was increased in a public dataset of KLRG1^+ ^Nfil3^ +^ terminal Tregs compared to KLRG1^−^Nfil3^ +^ transitional Tregs [11], while the Vps34-suppressed signature (derived from upregulated genes in Vps34-deficient versus control Tregs; see S2 Table and Materials and methods) was decreased in KLRG1^+ ^Nfil3^ +^ terminal Tregs compared to KLRG1^−^Nfil3^ +^ transitional Tregs (Fig 3F), indicating upregulation of Vps34 activity during terminal differentiation. Together, these results suggest that Vps34 is a crucial regulator of eTreg heterogeneity and terminal differentiation during perinatal life.

As KLRG1 and ST2 are surface markers expressed by terminally differentiated eTregs [11,50–53], we utilized these two surface markers and flow cytometry analysis to rigorously establish the differential requirement of Vps34 for the accumulation of transitional and terminal eTreg populations. Among splenic eTregs, there was a significant decrease in both the frequency and number of KLRG1^ +^ or ST2^ +^ terminal eTregs and a corresponding increase in the frequency (albeit not number) of KLRG1^−^ or ST2^−^ transitional eTregs from perinatal *Foxp3*^Cre^*Pik3c3*^fl/fl^ mice compared to their littermate controls (Fig 3G). Moreover, KLRG1^ +^ or ST2^ +^ terminal eTreg numbers were reduced in the lung (Fig 3H) and liver (S3C Fig). There were also modestly reduced numbers of KLRG1^−^ or ST2^−^ transitional eTregs in these tissues (Figs 3H and S3C), likely attributed to an overall reduction in eTreg cellularity (S3D Fig). KLRG1^+^ and ST2^+^ terminal eTreg numbers were also decreased in the spleen and especially lung of *Foxp3*^Cre^*Pik3c3*^fl/fl^ mixed BM chimeras compared to control counterparts (S3E and S3F Fig). Thus, Vps34 plays a cell-intrinsic role in mediating terminal eTreg accumulation in both lymphoid and non-lymphoid tissues.

Recent studies have established that precursors for non-lymphoid tissue Tregs exist in lymphoid tissues [11,13,14,16,52]. In particular, differential expression of PD-1 and KLRG1 distinguishes Treg populations with discrete potentials to differentiate into ST2^+ ^KLRG1^ +^ tissue-resident Tregs in non-lymphoid tissues, with PD-1^+ ^KLRG1^+^ Tregs representing a terminal and stable population that arises from PD-1^+^KLRG1^−^ Tregs [11]. We found that there was a drastic loss in PD-1^+ ^KLRG1^+^ Tregs, with PD-1^+ ^KLRG1^−^ Tregs remaining largely unaltered, in both spleen and lung (Fig 3I), further supporting the notion that Vps34 is selectively required for the formation of the most terminal Treg population. Finally, KLRG1 and ST2 are often co-expressed on terminally differentiated Tregs in non-lymphoid tissues [11,51–53], and we found that Vps34 deficiency led to a reduction of KLRG1^+ ^ST2^ +^ eTregs in the lung of perinatal *Foxp3*^Cre^*Pik3c3*^fl/fl^ mice compared to littermate control mice (Fig 3J). Together, these results suggest that Vps34 selectively orchestrates the accumulation of terminal eTregs in both lymphoid and non-lymphoid tissues, including those with co-expression of KLRG1 and ST2.

### Vps34 orchestrates transcriptional and epigenetic remodeling events corresponding to terminal eTreg differentiation during perinatal life

**Fig 4:**
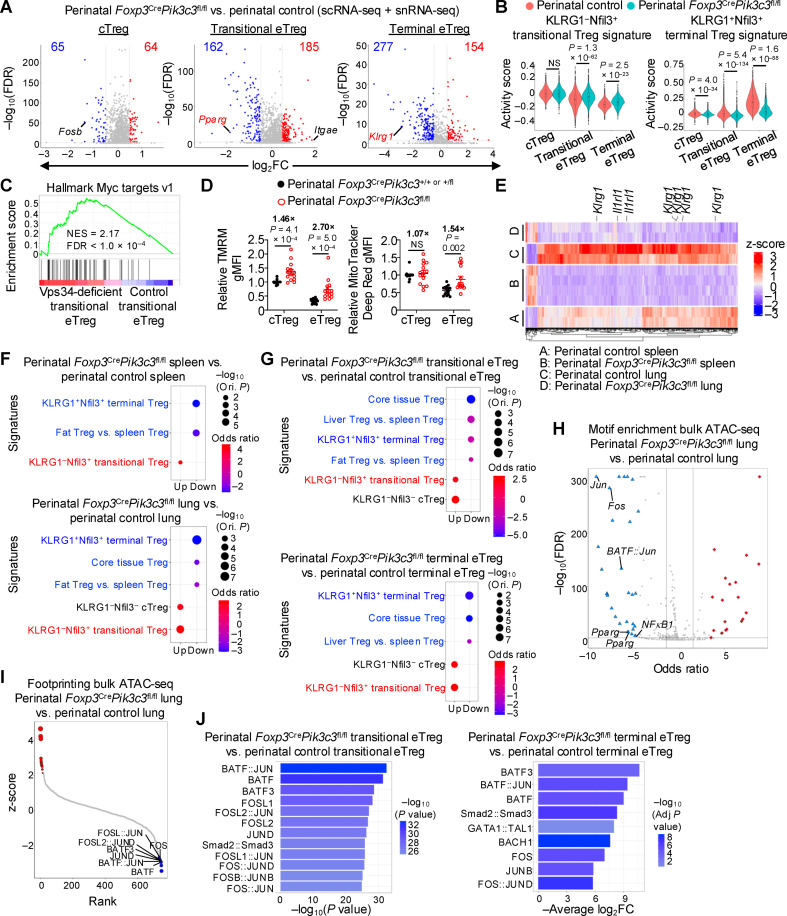
Vps34 orchestrates transcriptional and epigenetic remodeling events corresponding to terminal eTreg differentiation during perinatal life. (**A**) Volcano plots of differentially expressed genes (|log_2_FC | >  0.5, *P* < 0.05) in 10- to 12-day-old perinatal *Foxp3*^Cre^*Pik3c3*^fl/fl^ versus control mice. The three splenic Treg states (cTregs, left; transitional eTregs, middle; terminal eTregs, right) were identified via single-cell transcriptome profiling (see Fig 3 and Materials and methods for details). The numbers of upregulated and downregulated genes in the indicated comparisons are shown in red and blue, respectively, and certain transitional or terminal eTreg genes are labeled in red font. See also S3 Table. (**B**) Violin plots showing the activity scores of a curated KLRG1^− ^Nfil3^+^ transitional Treg signature (left; i.e., top 200 upregulated genes [log_2_FC >  0.5, FDR <  0.05]) in KLRG1^−^Nfil3^+^ Tregs compared to KLRG1^+ ^Nfil3^+^ Tregs from a public RNA-seq dataset GSE130884 [11]; see Materials and methods for details) and a curated KLRG1^+ ^Nfil3^+^ terminal Treg signature (right; top 200 upregulated genes [log_2_FC >  0.5, FDR <  0.05]) in KLRG1^+ ^Nfil3^+^ Tregs compared to KLRG1^−^Nfil3^+^ Tregs from a public RNA-seq dataset GSE130884 [11], see Materials and methods for details) in cTregs, transitional eTregs, and terminal eTregs from the single-cell transcriptomics data from 10- to 12-day-old perinatal control and *Foxp3*^Cre^*Pik3c3*^fl/fl^ mice. (**C**) GSEA enrichment plot showing increased Hallmark Myc targets signature in Vps34-deficient transitional eTregs versus control transitional eTregs from single-cell transcriptome profiling (described in Fig 3). GSEA, gene set enrichment analysis; NES, normalized enrichment score; FDR, false discovery rate. See also S4 Table. (**D**) Quantification of relative (normalized to average control in each experiment) gMFIs of TMRM and MitoTracker Deep Red in cTregs and eTregs derived from the spleen of 7- to 11-day-old perinatal control or *Foxp3*^Cre^*Pik3c3*^fl/fl^ mice, as determined by flow cytometry analysis (*n* = 15 for control and 14 for *Foxp3*^Cre^*Pik3c3*^fl/fl^ for TMRM and MitoTracker Deep Red). gMFI, geometric mean fluorescence intensity. (**E**, **F**) Total TCRβ^+^CD4^+ ^Foxp3-YFP^+^ Tregs were sort-purified from the spleen and lung of 10-day-old perinatal control (*n* = 4 for spleen, 2 for lung) and *Foxp3*^Cre^*Pik3c3*^fl/fl^ (*n* = 2 for both tissues) mice and profiled by bulk ATAC-seq (see Materials and methods for details). Heatmap depicting the differentially accessible chromatin regions (selected based on | log_2_FC | >  0.5, adjusted *P* < 0.05, see Materials and methods for details) that are shared between Vps34-deficient Tregs versus control Tregs in both the spleen and lung (e.g., *Klrg1* and *Il1rl1*), as determined by bulk ATAC-seq analysis. See also S4E Fig and S5 Table (**E**). PSEA of differentially accessible peaks (|log_2_FC | >  0.5, adjusted *P* < 0.05) in Tregs derived from the spleen (upper) or lung (lower) of 10-day-old perinatal *Foxp3*^Cre^*Pik3c3*^fl/fl^ versus perinatal control mice, using curated transcriptome signatures of the indicated Treg populations (derived from transcriptome profiling [11]; see Materials and methods for details). PSEA, peak set enrichment analysis (**F**). (**G**) scATAC-seq analysis (via multiome profiling) was performed in total TCRβ^+^CD4^+ ^Foxp3-YFP^+^ Tregs sort-purified from the spleen of 12-day-old perinatal control (*n* = 1) or *Foxp3*^Cre^*Pik3c3*^fl/fl^ (*n* = 2) mice. PSEA of differentially accessible peaks in transitional eTregs (upper; | log_2_FC | >  0.3, *P* < 0.05) or terminal eTregs (lower; | log_2_FC | >  0.5, *P* < 0.05) from the spleen of perinatal *Foxp3*^Cre^*Pik3c3*^fl/fl^ versus perinatal control mice, using public gene signatures [11] of the indicated Treg populations (see Materials and methods for details). (**H**) Transcription factor motif enrichment analysis of differentially accessible peaks (|log_2_FC | >  0.5, *P* <  0.05) from bulk ATAC-seq data (as described in **E**, **F**) of Tregs from the lung of perinatal *Foxp3*^Cre^*Pik3c3*^fl/fl^ versus perinatal control mice. Transcription factors with predicted decreased activity are labeled, including AP-1-related, PPARγ, and NFκB-related transcription factors. See also S7 Table. (**I**) Transcription factor footprinting analysis from bulk ATAC-seq data (as described in **E**, **F**) of Tregs from the lung of perinatal *Foxp3*^Cre^*Pik3c3*^fl/fl^ versus perinatal control mice, ranked based on z-scores. AP-1-related transcription factors with predicted decreased activity are labeled. See also S8 Table. (**J**) Transcription factor motif enrichment analysis of open chromatin regions with reduced accessibility (log_2_FC <  0) from scATAC-seq data (as described in **G**) of transitional eTregs from the spleen of perinatal *Foxp3*^Cre^*Pik3c3*^fl/fl^ versus perinatal control mice (left). ChromVAR analysis identified motifs with reduced activity in terminal eTregs from the spleen of perinatal *Foxp3*^Cre^*Pik3c3*^fl/fl^ versus perinatal control mice (right). Top transcription factors with predicted decreased activity are labeled, including AP-1-related transcription factors. See also S9 Table. Data are shown as mean ± s.e.m. (**D**). Wilcoxon rank-sum test (**A**, **B**; **J**, right), Benjamini–Hochbert test (**C**), two-tailed Student *t* test (**D**, **I**), Fisher’s exact test (**F**–**H**), or bionomial test (**J**, left); NS, not significant. Data are compiled from 10 independent experiments (**D**). The numerical data underlying the graphs shown in this figure are found in S4 Data (**D**). Raw scRNA-seq, snRNA-seq, bulk ATAC-seq, and scATAC-seq data used for analyses in (**A**–**C, E–J**) have been deposited to GEO SuperSeries access number GSE279606.

To establish molecular mechanisms by which Vps34 orchestrates terminal eTreg accumulation, we next performed comprehensive, unbiased multiomics profiling analyses. Differential expression analysis (from single-cell transcriptome profiling) revealed that Vps34 deficiency led to transcriptional alterations in cTregs and, to a greater extent, transitional eTregs and terminal eTregs (Fig 4A and S3 Table). Of note, Vps34-deficient transitional eTregs had reduced expression of *Pparg* (Fig 4A), a transcription factor that supports the stepwise differentiation of specialized populations of non-lymphoid tissue Tregs [13,16,55]. To establish whether Vps34 regulates terminal versus transitional eTreg transcriptional programming, we examined the activities of the KLRG1^−^Nfil3^ +^ transitional Treg and KLRG1^+ ^Nfil3^+^ terminal Treg gene signatures [11] within the perinatal Treg populations identified in our single-cell transcriptomics data. This analysis revealed that Vps34-deficient eTregs (both transitional and terminal) had increased activity of the KLRG1^−^Nfil3^ +^ transitional Treg gene signature [11] but reduced activity of the KLRG1^+ ^Nfil3^+^ terminal Treg gene signature [11] (Fig 4B), suggesting that Vps34 is important for dampening the transitional gene expression program while promoting the terminal program in perinatal eTregs.

Besides these effects on transitional and terminal Treg programs, GSEA using Hallmark gene sets revealed enriched Myc gene signature in transitional but not terminal eTregs (Fig 4C and S4 Table). Myc activity supports eTreg fitness [19,39,56] and mitochondrial metabolic reprogramming in Tregs [19], and Vps34-deficient eTregs (and cTregs) from perinatal *Foxp3*^Cre^*Pik3c3*^fl/fl^ mice had increased staining for TMRM (indicator for mitochondrial membrane potential) and MitoTracker Deep Red (indicator for mitochondrial content) (Fig 4D), with such effects also observed in eTregs (and cTregs) from *Foxp3*^Cre^*Pik3c3*^fl/fl^ chimeras (S4A Fig). Further, the levels of mitochondrial reactive oxygen species (ROS; based on MitoSOX staining) and total cellular ROS (based on CellROX staining) were increased in Vps34-deficient eTregs from perinatal (S4B Fig) and mixed BM chimera mice (S4A Fig), suggesting that mitochondrial fitness and redox balance are altered in the absence of Vps34. In contrast, the gene signature for Hallmark TNFα signaling via NFκB, a transcription factor essential for eTreg generation and function [57–59], was reduced in terminal but not transitional eTregs (S4C Fig and S4 Table). Together, these data reveal that Vps34 orchestrates transcriptional programming in perinatal Tregs and may coordinate eTreg functional adaptation via Myc- and NFκB-related transcriptional pathways.

Next, we assessed Vps34-dependent epigenetic alterations using two complementary approaches. First, we performed assay for transposase-accessible chromatin with high-throughput sequencing (ATAC-seq) analysis using total Tregs isolated from the spleen and lung (a site enriched for terminal ST2^+ ^KLRG1^+^ eTregs; see Fig 3J) of perinatal littermate control and *Foxp3*^Cre^*Pik3c3*^fl/fl^ mice. Principal component analysis (PCA) revealed marked chromatin alterations between the Treg populations from the spleen versus lung, with further changes induced by Vps34 deficiency in Tregs from the spleen and especially the lung (S4D Fig). In the spleen, 659 upregulated peaks and 1,137 downregulated peaks displayed differential accessibility in Vps34-deficient Tregs compared to control Tregs, whereas there were 1,486 upregulated peaks and 3,399 downregulated peaks with differential accessibility in Vps34-deficient versus control Tregs from the lung (S4E Fig and S5 Table). These results further support the notion that Vps34 orchestrates chromatin remodeling to a greater extent in lung Tregs than splenic Tregs. Of note, a subset of the differentially accessible peaks was shared between these two tissues (75 upregulated and 880 downregulated peaks) (S4E Fig), including the terminal eTreg-associated genes *Klrg1* and *Il1rl1* (Fig 4E and S5 Table), suggesting epigenetic regulation of terminal eTreg fate by Vps34.

To further establish Treg epigenetic programs orchestrated by Vps34, we first annotated the closest genes related to the differentially accessible peaks in Vps34-deficient versus control Tregs and then performed peak set enrichment analysis (PSEA) using curated gene signatures corresponding to distinct Treg transitional states [11] (namely, KLRG1^−^Nfil3^−^ cTregs, KLRG1^−^Nfil3^ +^ transitional Tregs, and KLRG1^+ ^Nfil3^ +^ terminal Tregs as described above in Fig 3C) and non-lymphoid tissue Tregs (e.g., fat or liver Tregs versus spleen Tregs and core tissue Tregs [11]; see Materials and methods for details). In both tissues, Vps34-deficient Tregs had reduced terminal and non-lymphoid tissue-associated epigenetic signatures, whereas those related to transitional Tregs were elevated (Fig 4F). Collectively, these results suggest that Vps34 is important for promoting terminal and non-lymphoid tissue Treg-associated epigenetic reprogramming in perinatal mice.

Second, we performed multiome analysis that combines single-cell ATAC-seq (scATAC-seq) with single-nuclear RNA-seq (snRNA-seq) to more specifically characterize Vps34-dependent chromatin alterations in each of the splenic Treg states. To further validate these annotations, we analyzed the chromatin accessibility of certain cTreg-, eTreg-, and terminal eTreg-associated genes. As expected, *Ccr7* and *Tcf7* had enhanced accessibility in cTregs, while genes associated with eTreg suppressive function and terminal differentiation (*Tigit*, *Il10*, *Klrg1*) [11,50,60–62] were more accessible in transitional and especially terminal eTregs (S4F Fig). Differential accessibility analysis of Vps34-deficient Treg states compared to control cells revealed altered chromatin profiles in all three Treg states, with the eTreg populations displaying the greatest number of differentially accessible peaks in the absence of Vps34 (S4G Fig and S6 Table). Among them, the accessibilities of *Il10* and *Klrg1* were decreased in the Vps34-deficient terminal eTregs compared to control counterparts (S4G Fig and S6 Table), further supporting the notion that Vps34 is important for orchestrating terminal eTreg-related epigenetic programming. Finally, PSEA of the scATAC-seq data revealed that Vps34-deficient transitional and terminal eTregs had decreased terminal and non-lymphoid tissue-associated gene signatures, whereas those corresponding to transitional eTregs were increased (Fig 4G), in line with the results from the bulk ATAC-seq analysis described above (Fig 4F). Together, these results reveal that Vps34 orchestrates terminal eTreg and non-lymphoid tissue Treg-associated epigenetic programming during perinatal life.

We next performed transcription factor motif enrichment and footprinting analyses [24,63] to infer changes in transcription factor activity in the absence of Vps34. In our bulk ATAC-seq profiling, both motif enrichment (Fig 4H and S7 Table) and footprinting (Fig 4I and S8 Table) analyses predicted decreased activity of several AP-1-related transcription factors (e.g., Jun, Fos, BATF) in Vps34-deficient perinatal Tregs from the lung as compared to control counterparts, with motif enrichment also predicting reductions in PPARγ- and NFκB-related transcriptional activities (Fig 4H and S7 Table). Similar reductions in AP-1-related transcription factors were observed in Vps34-deficient perinatal Tregs derived from the spleen (S4H and S4I Fig and S7 and S8 Tables). To extend these analyses to heterogenous Treg states, we performed motif enrichment analysis using the splenic Tregs profiled by the multiome analysis. We found that NFκB-related and Jun/Fos transcription factors were among the top-ranking transcription factors predicted to have decreased activity in Vps34-deficient cTregs (S4J Fig). Further, in both transitional and terminal eTregs, AP-1-related transcription factors (e.g., Jun, Fos, BATF) represented the majority of the top-ranked transcription factors with predicted decreased activities in the absence of Vps34 (Fig 4J), and NFκB1 activity was also reduced in transitional and terminal eTregs (ranked 66th and 32nd, respectively) (S9 Table). These results were notable, because AP-1-related transcription factors, including JunB and BATF, orchestrate eTreg differentiation and function, as well as non-lymphoid tissue Treg generation [11,53,61,64]. Collectively, these data suggest that Vps34 coordinates the transcriptional and epigenetic programming of Tregs during perinatal life and is an important regulator of AP-1-related transcription factor activity that supports eTreg transitional heterogeneity and functional adaptation.

### Atg14 but not Uvrag deficiency in Tregs leads to a cell-intrinsic reduction in eTregs

**Fig 5:**
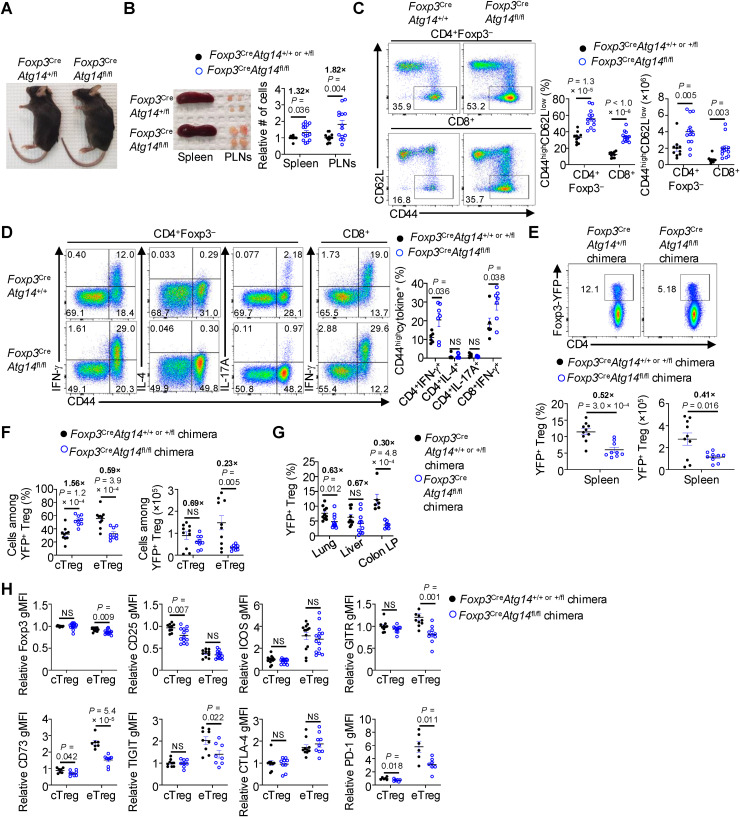
Atg14 deficiency in Tregs induces a type-I-dominant lymphoproliferative disease and cell-intrinsic reductions in eTregs. (**A**) Representative images of 5-month-old control and *Foxp3*^Cre^*Atg14*^fl/fl^ mice. (**B**) Representative images of spleen and PLNs (left) and quantification (right) of the relative (normalized to average control in each experiment) total cell numbers from indicated tissues of 5-month-old control (*n* =  11 for the spleen, 13 for the PLNs) or *Foxp3*^Cre^*Atg14*^fl/fl^ (*n* =  14 for both tissues) mice. PLNs, peripheral lymph nodes. (**C**) Flow cytometry analysis (left) and quantification (right) of frequencies and numbers of CD44^high^CD62L^low^ effector/memory cells among TCRβ^+^CD4^+ ^Foxp3-YFP^−^ (denoted as CD4^+ ^Foxp3^−^) and CD8^+^ (gated as CD4^−^TCRβ^+^) T cells derived from the spleen of 4- to 5-month-old control (*n* =  10) or *Foxp3*^Cre^*Atg14*^fl/fl^ (*n* =  13) mice. (**D**) Splenocytes were stimulated with PMA and ionomycin in the presence of GolgiStop for 4 h. Flow cytometry analysis (left) and quantification (right) of the frequencies of IFN-γ^+^, IL-4^+^, or IL-17A^+^ cells among ΤCRβ^+^CD4^+ ^Foxp3^− ^CD44^high^ conventional Τ cells and IFN-γ^+^ cells among ΤCRβ^+^CD8^+ ^CD44^high^ T cells from 4- to 5-month-old control (*n* =  6) or *Foxp3*^Cre^*Atg14*^fl/fl^ (*n* =  7) mice. (**E**−**H**) Mixed BM chimera mice were generated by adoptive transfer of a 1:1 mixture of BM from CD45.2^ +^ control or *Foxp3*^Cre^*Atg14*^fl/fl^ mice and CD45.1^ +^ mice into sublethally irradiated *Rag1*^−/−^ mice. BM, bone marrow. Flow cytometry analysis (upper) and quantification (lower) of frequency and number of total TCRβ^+^CD4^+ ^Foxp3-YFP^ +^ Tregs derived from the spleen of control (*n* =  10) or *Foxp3*^Cre^*Atg14*^fl/fl^ (*n* =  9) mixed BM chimera mice (**E**). Quantification of the frequencies (left) and numbers (right) of CD44^low^CD62L^high^ cTregs and CD44^high^CD62L^low^ eTregs among total TCRβ^+^CD4^+ ^Foxp3-YFP^ +^ Tregs derived from the spleen of control (*n* =  10) or *Foxp3*^Cre^*Atg14*^fl/fl^ (*n* =  9) mixed BM chimera mice (**F**). Quantification of total TCRβ^+^CD4^+ ^Foxp3-YFP^ +^ Treg frequency in the lung, liver, or colon LP of control (*n* =  10 for lung and liver and 6 for colon LP) or *Foxp3*^Cre^*Atg14*^fl/fl^ (*n* =  10 for lung and liver and 7 for colon LP) mixed BM chimera mice. LP, lamina propria (**G**). Quantification of relative (normalized to average control in each experiment) gMFIs of Foxp3 (*n* =  10 for control mixed BM chimeras; 13 for *Foxp3*^Cre^*Atg14*^fl/fl^ mixed BM chimeras), CD25 (*n* =  10 for control mixed BM chimeras; 13 for *Foxp3*^Cre^*Atg14*^fl/fl^ mixed BM chimeras), ICOS (*n* =  13 for control mixed BM chimeras; 14 for *Foxp3*^Cre^*Atg14*^fl/fl^ mixed BM chimeras), GITR (*n* =  10 for both control and *Foxp3*^Cre^*Atg14*^fl/fl^ mixed BM chimeras), CD73 (*n* =  7 for control mixed BM chimeras; 8 for *Foxp3*^Cre^*Atg14*^fl/fl^ mixed BM chimeras), TIGIT (*n* =  9 for control mixed BM chimeras; 8 for *Foxp3*^Cre^*Atg14*^fl/fl^ mixed BM chimeras), CTLA-4 (*n* =  10 for control mixed BM chimeras; 9 for *Foxp3*^Cre^*Atg14*^fl/fl^ mixed BM chimeras), and PD-1 (*n* =  6 for control mixed BM chimeras; 7 for *Foxp3*^Cre^*Atg14*^fl/fl^ mixed BM chimeras) in CD44^low^CD62L^high^ cTregs and CD44^high^CD62L^low^ eTregs among total TCRβ^+^CD4^+ ^Foxp3-YFP^ +^ (CD25, ICOS, GITR, CD73, PD-1) or TCRβ^+^CD4^+ ^Foxp3^+^ (Foxp3, TIGIT, CTLA-4) Tregs (all pre-gated on CD45.2^+^ cells) derived from the spleen of control or *Foxp3*^Cre^*Atg14*^fl/fl^ mixed BM chimera mice (**H**). Relative (normalized to average control in each experiment) fold-change comparisons between genotypes in each tissue (**B**, **G**) or cell type (**E**, **F**) are shown in bold. Data are shown as mean ±  s.e.m. (**B**–**H**). Two-tailed Student *t* test (**B**–**H**); NS, not significant. Data are compiled from 6 (**B**), 5 (**C**), ≥ 3 (**D**, **H**), 4 (**E**, **F**; **G**, lung and liver), or 2 (**G**, colon LP) independent experiments. Numbers in plots indicate percentages of cells in gates (**C**–**E**). The numerical data underlying the graphs shown in this figure are found in S5 Data (**B**–**H**).

Vps34 forms distinct signaling complexes with Atg14 or Uvrag to either initiate autophagy (complex I) or promote endosome maturation and endocytic trafficking to the lysosome (complex II), respectively [28,30]. While Atg14 function has been examined in myeloid cells [65,66] and the function of Uvrag has been explored in T cells [67], the roles of these molecules in Treg transitional heterogeneity and functional adaptation are unknown. Therefore, we generated mice with Treg-specific loss of either Atg14 (*Foxp3*^Cre^*Atg14*^fl/fl^ mice) or Uvrag (*Foxp3*^Cre^*Uvrag*^fl/fl^ mice) by crossing *Atg14*^fl/fl^ mice [68] or *Uvrag*^fl/fl^ mice [67] with *Foxp3*^YFP-Cre^ mice [37], thereby allowing for the genetic dissection of the contributions of Vps34 pro-autophagic versus pro-endocytic complexes to Treg fate and function in vivo. We verified the deletion efficiency of *Atg14* or *Uvrag* in Tregs from the abovementioned mice (S5A and S5B Fig). We first investigated whether Atg14 or Uvrag deficiency phenocopies the effects of Vps34 in promoting autoimmune-like disease in mice. *Foxp3*^Cre^*Atg14*^fl/fl^ mice showed no visual features of autoimmune-like disease (e.g., dermatitis) even at ≥5 months of age (Fig 5A), in striking contrast to the early-onset *Scurfy*-like phenotype that develops in *Foxp3*^Cre^*Pik3c3*^fl/fl^ mice (S2D Fig). Moreover, *Foxp3*^Cre^*Atg14*^fl/fl^ mice showed only a modest increase in the size of spleen and PLNs (Fig 5B), in contrast to the drastic splenomegaly and lymphadenopathy of *Foxp3*^Cre^*Pik3c3*^fl/fl^ mice (S2E Fig), suggesting that mice with Atg14-deficient Tregs develop a milder lymphoproliferative disease as compared to those with Vps34-deficient Tregs. However, no differences in cellularity were observed in the spleen or PLNs of *Foxp3*^Cre^*Uvrag*^fl/fl^ mice compared to control counterparts (S5C Fig). Thus, in contrast to Vps34, ablation of either Atg14 or Uvrag in Tregs does not promote fatal autoimmune-like disease, although loss of Atg14 in Tregs likely promotes excessive lymphoproliferation.

We next examined features of T cell homeostasis in *Foxp3*^Cre^*Atg14*^fl/fl^ and *Foxp3*^Cre^*Uvrag*^fl/fl^ mice. Flow cytometry profiling revealed that effector/memory CD4^ +^ and CD8^ +^ T cells were increased in *Foxp3*^Cre^*Atg14*^fl/fl^ mice compared to control mice (Fig 5C), whereas there were only trending minor increases in these populations in *Foxp3*^Cre^*Uvrag*^fl/fl^ mice (S5D Fig), indicating that loss of Atg14 but not Uvrag in Tregs disrupts T cell homeostasis. There were also increased frequencies of CD4^+ ^IFN-γ^+^ and CD8^+ ^IFN-γ^+^ T cells in *Foxp3*^Cre^*Atg14*^fl/fl^ mice compared to control mice (Fig 5D), indicating enhanced type-I inflammation. In contrast, no differences in cytokine production were observed in *Foxp3*^Cre^*Uvrag*^fl/fl^ mice compared to control mice (S5E Fig). Together, these findings suggest that Atg14 but not Uvrag contributes to Treg-dependent suppression of effector/memory T cell accumulation and type-I inflammation.

Given the cell-intrinsic role of Vps34 in shaping eTreg accumulation (as described in Fig 1), we next explored Treg-specific phenotypes upon ablation of either Atg14 or Uvrag. Consistent with the absence of autoimmune-like or inflammatory features, we observed a trending accumulation of total Tregs but no differences in cTreg and eTreg subsets in *Foxp3*^Cre^*Uvrag*^fl/fl^ mice compared to control counterparts (S5F and S5G Fig). Similarly, there were no differences in these populations in the cell-intrinsic system of *Foxp3*^Cre/+ ^*Uvrag*^fl/fl^ mosaic mice compared to control counterparts (S5H and S5I Fig), suggesting that Uvrag is dispensable for both total Treg and eTreg accumulation. In contrast, we found a significant decrease in Treg frequency (albeit not number) in the spleen of *Foxp3*^Cre^*Atg14*^fl/fl^ mice compared to control mice (S6A Fig). Further, there was a reduction in eTreg cellularity (S6B Fig). To circumvent the possible effects of inflammation and examine cell-intrinsic effects, we generated control *Foxp3*^Cre^*Atg14*^+/+ or +/fl^ and *Foxp3*^Cre^*Atg14*^fl/fl^ mixed BM chimeras. We observed significantly decreased total Tregs (Fig 5E) and eTregs (Fig 5F) in the spleen of *Foxp3*^Cre^*Atg14*^fl/fl^ mixed BM chimeras. Further, there were cell-intrinsic reductions of Tregs in the lung and cLP but not liver of *Foxp3*^Cre^*Atg14*^fl/fl^ mixed BM chimeras (Fig 5G). Of note, the reductions of non-lymphoid tissue Tregs and splenic eTregs were more pronounced in the absence of Vps34 than Atg14 (compare the fold-change values in Fig 1D and 1G with 5F and 5G), which may partly account for the overall reduced disease severity in mice bearing Atg14-deficient Tregs than those with Vps34-deficient Tregs.

Finally, we examined Treg suppressive markers in Atg14-deficient cTregs and eTregs from mixed BM chimeras. We found that loss of Atg14 expression in Tregs resulted in modestly decreased expression of Foxp3 (but not CD25) and markedly reduced expression of several Treg suppressive markers, including GITR, CD73, TIGIT, and PD-1 (but not ICOS or CTLA-4) in eTregs from *Foxp3*^Cre^*Atg14*^fl/fl^ mixed BM chimeras (Fig 5H). Atg14-deficient cTregs also had modest reductions of CD25, CD73, and PD-1 expression. The reductions of GITR, CD73, and PD-1 were shared features of both Vps34-deficient and Atg14-deficient cTregs and eTregs (compare Figs 5H and S2H), suggesting that the Atg14-containing Vps34 complex I partly contributes to Vps34-mediated suppressive function of Tregs in vivo. Altogether, these results establish a role for Vps34 complex I (containing Atg14) but not Vps34 complex II (containing Uvrag) in orchestrating eTreg and non-lymphoid Treg accumulation, as well as Treg function that is important for maintaining T cell homeostasis and curtailing type-I inflammation in vivo. However, only loss of Vps34 promotes the development of a fatal and early-onset autoimmune-like disease.

### Atg14 plays a cell-intrinsic role in orchestrating eTreg survival but not terminal differentiation

**Fig 6:**
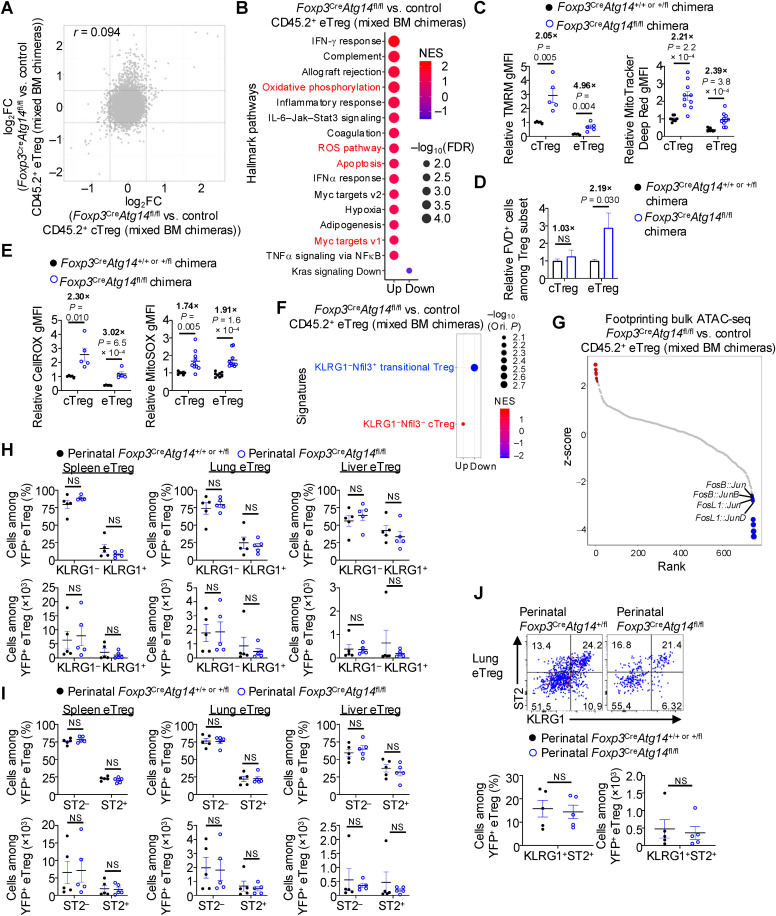
Atg14 plays a cell-intrinsic role in orchestrating eTreg survival but not terminal differentiation. (**A**, **B**) CD45.2^+ ^CD4^+ ^Foxp3-YFP^+^CD44^low^CD62L^high^ cTregs and CD45.2^+ ^CD4^+ ^Foxp3-YFP^+^CD44^high^CD62L^low^ eTregs were sort-purified from the spleen of control (*n* =  4) or *Foxp3*^Cre^*Atg14*^fl/fl^ (*n* =  3) mixed BM chimera mice and profiled by microarray analysis (see Materials and methods for details). Fold-change/fold-change plot of transcriptome profiling of Atg14-deficient eTregs versus control eTregs (y-axis) compared with Atg14-deficient cTregs versus control cTregs (x-axis). *r*, Pearson’s correlation coefficient (**A**). Bubble plot of GSEA results showing upregulated (Up) or downregulated (Down) Hallmark pathway signatures in CD45.2^+ ^CD4^+ ^Foxp3-YFP^+^CD44^high^CD62L^low^ eTregs from *Foxp3*^Cre^*Atg14*^fl/fl^ mixed BM chimera mice compared to control counterparts. GSEA, gene set enrichment analysis; NES, normalized enrichment score; FDR, false discovery rate (**B**). (**C**) Quantification of relative (normalized to average control in each experiment) gMFIs of TMRM (*n* =  5 per group) and MitoTracker Deep Red (*n* =  8 for control mixed BM chimera mice and 10 for *Foxp3*^Cre^*Atg14*^fl/fl^ mixed BM chimera mice) in TCRβ^+^CD4^+ ^Foxp3-YFP^+^CD44^low^CD62L^high^ cTregs and TCRβ^+^CD4^+ ^Foxp3-YFP^+^CD44^high^CD62L^low^ eTregs derived from the spleen of control or *Foxp3*^Cre^*Atg14*^fl/fl^ mixed BM chimera mice, as determined by flow cytometry analysis. gMFI, geometric mean fluorescence intensity. (**D**) Quantification of the relative frequencies (normalized to average control in each experiment) of FVD^ +^ non-viable CD44^low^CD62L^high^ cTregs and CD44^high^CD62L^low^ eTregs among total TCRβ^+^CD4^+ ^Foxp3^ +^ Tregs (all pre-gated on CD45.2^ +^ cells) derived from the spleen of control (*n* = 10) or *Foxp3*^Cre^*Atg14*^fl/fl^ (*n* = 9) mixed BM chimera mice. FVD, fixable viability dye. (**E**) Quantification of relative (normalized to average control in each experiment) gMFIs of CellROX (*n* = 5 per group) and MitoSOX (*n* = 8 for control mixed BM chimera mice and 10 for *Foxp3*^Cre^*Atg14*^fl/fl^ mixed BM chimera mice) in TCRβ^+^CD4^+ ^Foxp3-YFP^+^CD44^low^CD62L^high^ cTregs and TCRβ^+^CD4^+ ^Foxp3-YFP^+^CD44^high^CD62L^low^ eTregs derived from the spleen of control or *Foxp3*^Cre^*Atg14*^fl/fl^ mixed BM chimera mice, as determined by flow cytometry analysis. (**F**, **G**) CD45.2^+ ^CD4^+ ^Foxp3-YFP^+^CD44^low^CD62L^high^ cTregs and CD45.2^+ ^CD4^+ ^Foxp3-YFP^+^CD44^high^CD62L^low^ eTregs were sort-purified from the spleen of control (*n* =  3) and *Foxp3*^Cre^*Atg14*^fl/fl^ (*n* =  4) mixed BM chimera mice and profiled by bulk ATAC-seq (see Materials and methods for details). PSEA of differentially accessible peaks in eTregs (|log_2_FC | >  0.5, *P* <  0.05) derived from the spleen of *Foxp3*^Cre^*Atg14*^fl/fl^ mixed BM chimera mice versus control counterparts, using public gene signatures [11] of the indicated Treg populations (see Materials and methods for details) (**F**). PSEA, peak set enrichment analysis. Transcription factor footprinting analysis from bulk ATAC-seq data of eTregs derived from the spleen of *Foxp3*^Cre^*Atg14*^fl/fl^ mixed BM chimera mice versus control counterparts, ranked based on z-scores. Jun/Fos transcription factors with predicted decreased activity in Atg14-deficient versus control eTregs are labeled. See also S13 Table (**G**). (**H**) Quantification of the frequencies (upper) and numbers (lower) of KLRG1^−^ and KLRG1^+^ cells among TCRβ^+^CD4^+ ^Foxp3-YFP^+^CD44^high^CD62L^low^ eTregs derived from the spleen (left), lung (middle), or liver (right) of 7- to 11-day-old perinatal control or *Foxp3*^Cre^*Atg14*^fl/fl^ mice (*n* =  5 per group). (**I**) Quantification of the frequencies (upper) and numbers (lower) of ST2^ −^ and ST2^ +^ cells among TCRβ^+^CD4^+ ^Foxp3-YFP^+^CD44^high^CD62L^low^ eTregs derived from the spleen (left), lung (middle), or liver (right) of 7- to 11-day-old perinatal control or *Foxp3*^Cre^*Atg14*^fl/fl^ mice (*n* =  5 per group). (**J**) Flow cytometry analysis (upper) and quantification (lower) of the frequency and numbers of KLRG1^+ ^ST2^ +^ cells among TCRβ^+^CD4^+ ^Foxp3-YFP^+^CD44^high^CD62L^low^ eTregs from the lung of control or *Foxp3*^Cre^*Pik3c3*^fl/fl^ mice (*n* =  5 per group). Relative (normalized to average control in each experiment) fold-change comparisons between genotypes in each cell type (**C**–**E**) are shown in bold. Data are shown as mean ±  s.e.m. (**C**–**E**, **H**–**J**). Pearson’s correlation (**A**), Benjamini–Hochberg test (**B**), two-tailed Student *t* test (**C**–**E, G**–**J**), or Fisher’s exact test (**F**); NS, not significant. Data are compiled from ≥2 (**C**, **E**) or 4 (**D**, **H**–**J**) independent experiments. Numbers in plots indicate percentages of cells in gates (**J**). The numerical data underlying the graphs shown in this figure are found in S6 Data (**C**–**E, H**–**J**). Raw microarray and bulk ATAC-seq data used for analyses in (**A**, **B**, **F**, **G**) have been deposited to GEO SuperSeries access number GSE279606.

The cell-intrinsic reductions in eTregs in *Foxp3*^Cre^*Atg14*^fl/fl^ mixed BM chimeras prompted us to investigate the underlying mechanisms. To this end, we first tested whether Atg14-deficient Tregs can be activated in response to TCR stimulation in vitro as described above [24,43], and found that Atg14 deficiency had no impact on the generation of eTreg-like cells in vitro (S6C Fig), suggesting that these cells can undergo activation in response to TCR stimulation. Second, we performed Ki67 staining and found no significant differences in the proportions of Ki67^ +^ cells among total Tregs, cTregs, or eTregs derived from control or *Foxp3*^Cre^*Atg14*^fl/fl^ mixed BM chimeras (S6D Fig), indicating that the observed decreases in Treg accumulation across tissues are not due to a proliferation defect. Third, we sort-purified CD45.2^+^ cTregs and eTregs from control or *Foxp3*^Cre^*Atg14*^fl/fl^ mixed BM chimeras, followed by transcriptome profiling via microarray analysis. Differential expression analysis revealed a greater number of altered genes in Atg14-deficient eTregs versus control eTregs (589 total genes; S10 Table) compared to Atg14-deficient cTregs versus control cTregs (109 total genes; S10 Table), with minimal overlap between them based on fold-change/fold-change plot analysis (Fig 6A). These results are in line with the notion that eTregs are the major population impacted by Atg14 ablation. GSEA using the Hallmark gene sets revealed that several pathways were upregulated in Atg14-deficient eTregs, including metabolism-associated pathways (e.g., Hallmark oxidative phosphorylation and Myc signaling) (Fig 6B and S11 Table). Accordingly, Atg14-deficient eTregs had increased levels of TMRM and MitoTracker Deep Red staining (Fig 6C), similar to those effects described above for Vps34-deficient Treg subsets (S4A Fig). Thus, the Atg14-containing Vps34 complex affects the mitochondrial fitness of Tregs.

Notably, Atg14-deficient eTregs were enriched for the Hallmark apoptosis gene signature (Fig 6B and S11 Table). Accordingly, there were increased proportions of FVD^ +^ non-viable eTregs from both *Foxp3*^Cre^*Atg14*^fl/fl^ mixed BM chimeras and *Foxp3*^Cre/+^*Atg14*^fl/fl^ mosaic mice compared to respective control counterparts (Figs 6D and S6E), as well as an increased Bim/Mcl1 (but not Bim/Bcl2) ratio in Atg14-deficient eTregs from mixed BM chimeras (S6F Fig). These data indicate that a cell-intrinsic survival defect occurs in Atg14-deficient eTregs, consistent with the aforementioned observation that Vps34 supports eTreg survival (as described in Fig 1). GSEA also revealed increased Hallmark ROS signature in Atg14-deficient eTregs (Fig 6B and S11 Table). Accordingly, we found significantly increased total cellular and mitochondrial ROS in both cTregs and eTregs from *Foxp3*^Cre^*Atg14*^fl/fl^ mixed BM chimeras compared to control counterparts (Fig 6E), with such effects also similar to those observed in Tregs lacking Vps34 (S4A Fig).

To examine whether Treg-associated epigenetic programs are orchestrated by Atg14, we sort-purified cTregs and eTregs from the spleen of control and *Foxp3*^Cre^*Atg14*^fl/fl^ mixed BM chimeras and performed bulk ATAC-seq analysis. PCA revealed minor global differences between Atg14-deficient cTregs or eTregs compared to their control counterparts (S6G Fig). However, detailed analysis revealed more differentially accessible chromatin regions in Atg14-deficient eTregs versus control eTregs (with 2,732 upregulated and 1,471 downregulated regions) compared to Atg14-deficient cTregs versus control cTregs (with 1,459 upregulated and 1,016 downregulated regions) (S12 Table), further supporting the notion that Atg14 deficiency has a greater impact on eTregs than cTregs. Next, we annotated the closest genes related to the differentially accessible peaks in Atg14-deficient versus control cTregs or eTregs, and then applied our previously curated gene signatures associated with Treg transitional states or tissue Tregs [11] as described above. In contrast to Vps34 deficiency (Fig 4F and 4G), Atg14-deficient eTregs did not exhibit reductions in KLRG1^+ ^Nfil3^ +^ terminal Treg- or non-lymphoid tissue Treg-associated epigenetic programs. Instead, Atg14-deficient eTregs had a reduced KLRG1^−^Nfil3^ +^ transitional Treg epigenetic signature and an increased KLRG1^−^Nfil3^−^ cTreg epigenetic signature (Fig 6F). Further, transcription factor footprinting analysis [24,63] predicted that Atg14-deficient eTregs had decreased transcriptional activity of Jun/Fos but without alterations in BATF activity (Fig 6G and S13 Table). Together, these results establish that Atg14 regulates Jun/Fos activity but is dispensable for BATF activity and terminal and non-lymphoid tissue eTreg-associated epigenetic programs.

Based on these results, we next explored whether Atg14 orchestrates perinatal Treg fate and function. Flow cytometry profiling revealed no significant differences in effector/memory CD4^ +^ or CD8^ +^ T cells (S6H Fig) or cytokine-producing CD4^+ ^IFN-γ^+^ or CD8^+ ^IFN-γ^+^ T cells (S6I Fig), indicating that loss of Atg14 in Tregs is not associated with disrupted T cell homeostasis in perinatal mice. Further, there were no significant alterations in total Tregs, cTregs, or eTregs in perinatal *Foxp3*^Cre^*Atg14*^fl/fl^ mice compared to littermate control mice (S6J Fig). Moreover, Treg frequencies and numbers were similar in the lung and liver of perinatal *Foxp3*^Cre^*Atg14*^fl/fl^ mice and littermate control counterparts (S6K Fig). Thus, unlike Vps34, Atg14 is dispensable for Treg function and the accumulation of eTregs and non-lymphoid tissue Tregs in the perinatal environment.

Given the essential role of Vps34 in orchestrating eTreg transitional heterogeneity (described in Fig 3), we next examined whether Atg14 regulates this process. We found that eTregs from perinatal *Foxp3*^Cre^*Atg14*^fl/fl^ mice did not have defects in survival as revealed by FVD staining (S6L Fig), suggesting that Atg14 is not required for eTreg maintenance in the perinatal environment. Further, we observed no differences in KLRG1^ +^ or ST2^ +^ terminal eTregs (or KLRG1^ −^ or ST2^−^ transitional eTregs) in the spleen, liver, or lung of perinatal *Foxp3*^Cre^*Atg14*^fl/fl^ mice compared to littermate control counterparts (Fig 6H and 6I), in sharp contrast to Vps34 deficiency that led to a reduction of KLRG1^ +^ and ST2^ +^ terminal eTreg populations in perinatal mice (Figs 3G, 3H, and S3C). KLRG1^+ ^ST2^+^ eTregs were also unaltered in the lung of perinatal *Foxp3*^Cre^*Atg14*^fl/fl^ mice (Fig 6J), with such observation in contrast to loss of the KLRG1^+ ^ST2^+^ eTreg population observed in perinatal *Foxp3*^Cre^*Pik3c3*^fl/fl^ mice (Fig 3J). Thus, unlike Vps34, Atg14 is not required for the accumulation of terminal eTregs in lymphoid or non-lymphoid tissues during perinatal life. Rather, Atg14 selectively orchestrates the survival of eTregs to support their maintenance in lymphoid and selective non-lymphoid tissues.

### Targeting of Vps34 or Atg14, but not Uvrag, in Tregs reduces tumor growth in mouse models

**Fig 7:**
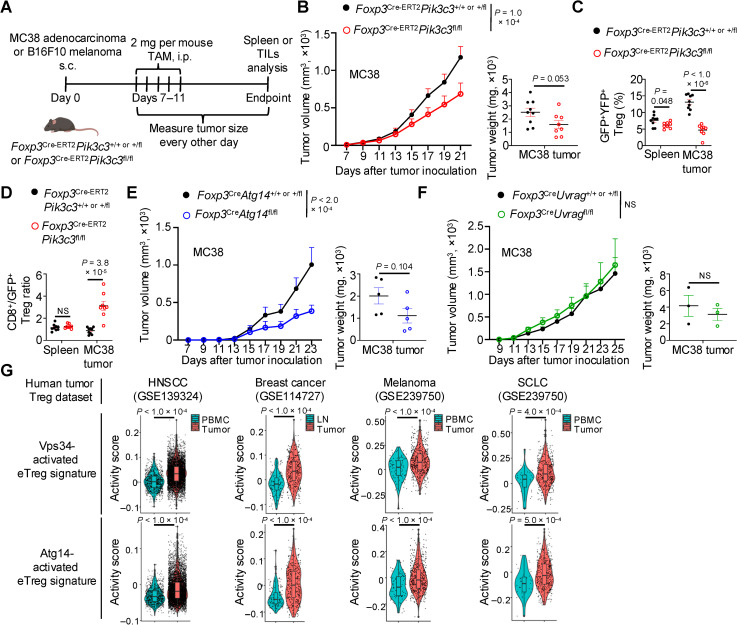
Targeting of Vps34 or Atg14, but not Uvrag, in Tregs reduces tumor growth in mouse models. (**A**) Experimental schematic for ablation of Vps34 in mature Tregs during early tumorigenesis. Control or *Foxp3*^Cre-ERT2^*Pik3c3*^fl/fl^ mice were subcutaneously (s.c.) inoculated with MC38 colon adenocarcinoma cells (5 × 10^5^) or B16F10 melanoma cells (2.5 × 10^5^; see S7A–C Fig) in the right flank. On days 7 to 11 after tumor inoculation, i.p. injections of TAM (2 mg/mouse dissolved in corn oil) were given daily for a total of 5 injections, and tumor size was measured every other day from 7 (MC38) or 11 (B16F10) days after tumor inoculation until endpoint tumor volume (1.5 × 10^3^ mm^3^) or humane endpoint was reached. i.p., intraperitoneal; TAM, tamoxifen. Mouse image created with BioRender. (**B**−**D**) Control (*n* =  9) or *Foxp3*^Cre-ERT2^*Pik3c3*^fl/fl^ (*n* =  8) mice were inoculated with MC38 tumors and treated with TAM as described in (**A**). Tumor growth curves (left) and tumor weights (right) at endpoint (day 21) in control or *Foxp3*^Cre-ERT2^*Pik3c3*^fl/fl^ mice (**B**). Quantification of the frequency of control or Vps34-deficient TCRβ^+^CD4^+ ^GFP^+^YFP^ +^ Tregs derived from the spleen or MC38 tumor of control or *Foxp3*^Cre-ERT2^*Pik3c3*^fl/fl^ mice at day 21 after tumor inoculation (**C**). Quantification of the ratio of total TCRβ^+^CD8^ +^ T cells to total TCRβ^+^CD4^+ ^GFP^ +^ Tregs derived from the spleen or MC38 tumor of control or *Foxp3*^Cre-ERT2^*Pik3c3*^fl/fl^ mice at day 21 after tumor inoculation (**D**). (**E**) Tumor growth curves (left) and tumor weights (right) at endpoint (day 23) in control or *Foxp3*^Cre^*Atg14*^fl/fl^ mice inoculated with MC38 tumors (*n* =  5 per group). (**F**) Tumor growth curves (left) and tumor weights of non-ulcerated tumors (right) at endpoint (day 25) in control (*n* =  4 for tumor growth; 3 for tumor weight) or *Foxp3*^Cre^*Uvrag*^fl/fl^ (*n* =  6 for tumor growth; 3 for tumor weight) mice inoculated with MC38 tumors. (**G**) Violin plots showing the activity scores of a curated Vps34-activated eTreg signature (upper; i.e., top 200 [ranked by log_2_FC] downregulated genes [log_2_FC ≤  –0.5 and FDR <  0.05] in Vps34-deficient eTregs [transitional +  terminal] versus control eTregs [transitional +  terminal] from single-cell transcriptome profiling as described in Fig 3; see S14 Table and Materials and methods for details) or curated Atg14-activated eTreg signature (lower; i.e., downregulated genes [log_2_FC ≤  −0.5 and *P* <  0.05] in Atg14-deficient eTregs versus control eTregs from mixed BM chimera mice; see S14 Table and Materials and methods for details) in publicly available scRNA-seq datasets of human Tregs from PBMCs, LN, or tumors in public datasets (GSE139324 [69]; GSE114727 [70]; GSE239750 [71] as indicated). BM, bone marrow; LN, lymph node; PBMC, peripheral blood mononuclear cells. Data are shown as mean ±  s.e.m. (**B**–**F**). Two-way ANOVA (tumor volume; **B**, **E**, **F**), Welch’s *t* test (tumor weight; **B**, **E, F**), two-tailed Student *t* test (**C**, **D)**, or Wilcoxon rank-sum test (**G**); NS, not significant. Data are representative of 6 (**B**–**D**) or 2 (**E**, **F**) independent experiments. The numerical data underlying the graphs shown in this figure are found in S7 Data (**B**–**F**). Genes in the Vps34-activated eTreg signature or Atg14-activated eTreg signature are shown in S14 Table and were applied to publicly available gene sets as described in the figure and figure legend (**G**).

Tregs represent immunotherapeutic targets for cancer due to their immunosuppressive role within the TME [3]. To explore the therapeutic effect of targeting Vps34 in mature Tregs during early tumorigenesis, we subcutaneously inoculated control *Foxp3*^Cre-ERT2^*Pik3c3*^+ / + or + /fl^ or *Foxp3*^Cre-ERT2^*Pik3c3*^fl/fl^ mice with MC38 colon adenocarcinoma or B16F10 melanoma cells. This inducible deletion system was chosen to circumvent the early lethality and fatal autoimmune-like disease of *Foxp3*^Cre^*Pik3c3*^fl/fl^ mice (described in Fig 2). At days 7 to 11 after tumor inoculation, we intraperitoneally injected the mice with daily tamoxifen for a total of 5 injections and monitored tumor growth every other day until the humane endpoint [22] (Fig 7A). In the MC38 tumor model, inducible deletion of Vps34 led to decreases in both tumor growth and tumor weight as compared to tamoxifen-treated control mice (Fig 7B), suggesting that Vps34 contributes to Treg-dependent suppression of anti-tumor immune responses. Flow cytometry profiling revealed a significantly reduced proportion of Vps34-deficient (GFP^+^YFP^+^) Tregs in the tumor (and modest reduction in the spleen) of tamoxifen-treated *Foxp3*^Cre-ERT2^*Pik3c3*^fl/fl^ mice compared to control counterparts (Fig 7C). Further, the ratio of total TCRβ^+^CD8^ +^ T cells to total GFP^ +^ Tregs (among TCRβ^+^CD4^+^ cells) was increased in the tumor (albeit not spleen) of *Foxp3*^Cre-ERT2^*Pik3c3*^fl/fl^ mice (Fig 7D), indicating enhanced anti-tumor immunity [22]. Similar effects of inducible Vps34 ablation were observed in the B16F10 tumor model (S7A–S7C Fig). Thus, targeting of Vps34 in Tregs during early tumorigenesis improves anti-tumor immunity.

To explore whether Atg14 or Uvrag mediates Treg-dependent suppression of anti-tumor immunity, we next inoculated *Foxp3*^Cre^*Atg14*^fl/fl^ or *Foxp3*^Cre^*Uvrag*^fl/fl^ mice and their respective control counterparts with MC38 tumors. MC38 tumor growth was significantly decreased in *Foxp3*^Cre^*Atg14*^fl/fl^ mice compared to their control counterparts, corresponding with a trending decrease in tumor weight (Fig 7E). In contrast, no differences in tumor growth or weight were observed between control and *Foxp3*^Cre^*Uvrag*^fl/fl^ mice (Fig 7F), indicating that Tregs do not require Uvrag to promote suppression of anti-tumor immune responses. These results reveal that Atg14, but not Uvrag, in Tregs orchestrates suppression of anti-tumor immune responses.

Next, we evaluated the clinical relevance of Vps34 or Atg14 activity in Tregs within the TME. Given that eTregs accumulate in tumors [3] and that Vps34 and Atg14 are both important for eTreg accumulation (as described above), we generated a Vps34-activated eTreg gene signature from the single-cell transcriptome data described in Fig 3 (i.e., genes downregulated in Vps34-deficient total eTregs compared to control total eTregs; see Materials and methods for details) (S14 Table) and an Atg14-activated eTreg gene signature from our transcriptome profiling described in Fig 6 (i.e., genes downregulated in Atg14-deficient eTregs compared to control eTregs; see Materials and methods for details) (S14 Table). Then, we applied these signatures to publicly available scRNA-seq datasets profiling Tregs from human tumor types, including head and neck squamous cell carcinoma (HNSCC) [69], breast cancer [70], melanoma [71], and small cell lung cancer (SCLC) [71] compared to those from non-tumor tissues (either patient-matched peripheral blood mononuclear cells or lymph nodes [LNs] as indicated). We found that in all four human cancer types, there were enrichments in the Vps34-activated and Atg14-activated gene signatures in intratumoral Tregs compared to their non-tumor tissue counterparts (Fig 7G), indicating that Vps34 and Atg14 may regulate the function of intratumoral Tregs in human cancer. Collectively, these results establish that Vps34 and Atg14 orchestrate the accumulation and/or immunosuppressive function of intratumoral Tregs.

## Discussion

Tregs help establish immune tolerance and mediate tumor immune suppression [1–3], but we have limited understanding of the mechanisms shaping context-dependent Treg function. There is also growing interest in understanding the spatiotemporal regulation of Treg biology, considering the distinct functional roles of perinatal and non-lymphoid tissue Tregs in preserving immune and tissue tolerance [8–10], as well as intratumoral Tregs in suppressing anti-tumor immunity [3]. Here, we establish discrete Vps34-orchestrated signaling processes in dictating terminal eTreg generation during perinatal life (independently of Atg14- or Uvrag-containing Vps34 complex I or II) and overall eTreg survival after perinatal life (via Vps34 complex I but not complex II). Further, we show that Vps34 complex I inhibition can unleash anti-tumor immunity in mouse tumor models, with possible extensions to human cancer. Together, these results establish roles for metabolic signaling, mediated by Vps34, in orchestrating the transitional heterogeneity and maintenance of eTregs, as well as the strong impacts of such functional adaptation on autoimmunity versus anti-tumor immunity (S7D Fig).

Conventional T cell heterogeneity plays an important role in the outcome of infections, cancer, and other diseases [72–74]. Recently, heterogenous Treg states have been described in lymphoid [11–14,16,52], non-lymphoid [11,12,52], and tumor [69–71,75] tissues, with discrete functional effects attributed to these populations. Moreover, there has been a particular focus on the molecular processes that dictate cTreg versus eTreg fate, because eTregs display greater immunosuppressive function and are enriched in both non-lymphoid and tumor tissues [1–3]. In lymphoid tissues, transitional states of activated Tregs with differential capacity to acquire non-lymphoid tissue residency programs have also been recently described in mice [11,13,14,16,76] and humans [77]. These include Nfil3^+ ^PD-1^+ ^KLRG1^−^ transitional Tregs, which can differentiate into Nfil3^+ ^PD-1^+ ^KLRG1^ +^ terminal Tregs that represent a stable population of Tregs enriched in non-lymphoid tissues [11]. Non-lymphoid tissue Treg differentiation from transitional Treg populations is orchestrated by transcription factors (e.g., PPARγ, Nfil3, BATF, Id2, and Id3) [11,13,14,16], as well as DNA methylation and chromatin remodeling events [11–13,60]. However, except for selective transcription factors (e.g., Foxp3; downregulation of Id3) [7,13,14] and extracellular cues (e.g., TCR, IL-33) [7,11,13,53,60], we have limited understanding of the molecular events that orchestrate transitional versus terminal eTreg programming, especially during perinatal life when immune and tissue tolerance-promoting Tregs arise [8–10]. Our study establishes Vps34 as an essential regulator of eTreg transitional heterogeneity that promotes terminal eTreg accumulation in both lymphoid and non-lymphoid tissues of perinatal mice. Mechanistically, our multiomics profiling revealed that Vps34 orchestrates transcriptional and epigenetic remodeling that increases terminal (and decreases transitional) eTreg and non-lymphoid tissue Treg programming, with such effects likely being partly dependent upon PPARγ, NFκB, Jun/Fos, and/or BATF activities that regulate eTreg fate and function [11,53,57–59,61,64]. Specifically, BATF functions as a pioneering transcription factor in CD4^ +^ T cells by promoting chromatin opening and recruitment of various transcription factors [78,79], including NFκB in intratumoral Tregs [71,80]. Moreover, BATF together with IRF4 induces PPARγ expression to promote the generation of Tregs in visceral adipose tissue [53]. Further, BATF cooperates with JunB, Blimp-1, and IRF4 to promote eTreg generation and non-lymphoid tissue Treg programming [11,61,64], with NFκB also contributing to these events via acting on distinct transcriptional programs [57–59]. Although loss of Atg14 is associated with reduced Jun/Fos activity in Tregs, Atg14 appears to be dispensable for BATF activation, suggesting that Vps34-dependent BATF activation is likely independent of Atg14. Overall, future studies are warranted to determine how these transcription factors are activated downstream of Vps34.

Metabolic rewiring is an essential regulator of immune cell fate decisions [17]. Further, metabolism-associated signaling processes are key regulators of cellular biology, including in T cells [81, 82]. In particular, metabolic signaling events orchestrated by lipids are emerging as critical regulators of Treg functional fitness. For example, the de novo mevalonate and lipid biosynthesis pathways differentially regulate signaling events in Tregs to shape their functional adaptation under homeostasis and in the TME [22–24]. However, aside from class I PI3K-dependent signaling [25–27,83], we have limited understanding of the physiological effects of other PI3K-derived phosphoinositides in Treg biology. In this study, we showed that the class III PI3K Vps34 orchestrates the functional fitness of Tregs for both the establishment of immune tolerance and suppression of anti-tumor immunity. Similar results were recently observed in an independent study, including in tumor models wherein Vps34 was inhibited in Tregs at the onset of tumor inoculation [84]. Moreover, consistent with findings in conventional T cells and Tregs [35,85], we found that Vps34 also regulated the metabolic fitness of Tregs, with Vps34-deficient eTregs displaying increased mitochondrial activity and ROS accumulation that may be regulated by enhanced Myc activity [19]. Similar to BATF, other AP-1 transcription factors, or NFκB [11,53,57–59,61,64], Myc is required for eTreg generation, but it is dispensable for their maintenance [19,56]. In contrast, excessive Myc activity, which can occur upon autophagy inhibition, leads to a loss of eTreg fitness, partly because it promotes hyperactivated glycolytic metabolism that may contribute to excessive ROS production [39,86]. Of note, we found that Vps34 and Atg14 deficiencies both cause elevated Myc activity, suggesting that autophagy defects also contribute to aberrant Myc activation in eTregs lacking those molecules. In terms of cell death, aberrant ROS accumulation in the absence of Vps34 contributes to the defective homeostasis or survival of NK cells [87], dendritic cells [88], and Tregs [84]. Mechanistically, excessive ROS levels may promote increased expression of Bim in either Vps34- or Atg14-deficient Tregs [89], whereas ineffective BATF-mediated repression of *Bcl2l11* (encoding Bim) transcription [90] could also contribute to excessive Bim expression in Vps34-deficient Tregs, thereby functionally overriding Mcl1- or Bcl2-dependent inhibition of Bim to promote Treg cell death [44, 45]. It is also possible that mitochondria metabolism-orchestrated epigenetic events contribute to the increased transcriptional and/or epigenetic remodeling of Vps34-deficient eTregs [82,91,92], which remains to be explored.

Vps34 forms multiprotein signaling complexes that dictate its downstream functions [28,30]. In particular, the pro-autophagy Vps34 complex I is defined by Atg14 (also called Atg14L), whereas Vps34 complex II is defined by Uvrag, which largely plays roles in regulating endocytic processes, including cargo sorting in multivesicular bodies and endosome–lysosome fusion that may contribute to autophagy [28,30]. Similar to Vps34 [31,33,34], naive CD4^+^ T cells lacking autophagy-related genes such as Atg5 or Atg7 [93,94] or Uvrag [67] exhibit reduced survival, suggesting that both autophagy and endocytosis regulate naïve T cell homeostasis downstream of Vps34. In contrast, our study shows that Atg14 but not Uvrag deficiency in Tregs only partially recapitulates the effects of Vps34 inhibition, including the development of a mild lymphoproliferative disease (but not *Scurfy*-like autoimmunity) and enhanced anti-tumor immunity. Such results are consistent with the notion that mice bearing Tregs lacking the autophagy-related proteins Atg7 or Atg16L develop late-onset inflammatory phenotypes that are associated with reduced eTreg fitness and improved tumor control [39,84,95]. Whether autophagy and endocytosis play functionally redundant roles to regulate Vps34-dependent terminal eTreg differentiation or if such effects are mediated by Atg14- or Uvrag-independent functions could be explored in future studies. Alternatively, Vps34 may integrate upstream signals that promote the terminal eTreg or non-lymphoid tissue Treg programming or accumulation, such as TCR- [5,13,76], TNF-receptor superfamily- [57], or IL-33 [11,13,53,60]-dependent signals, which warrants further investigation.

Collectively, our results establish a “two-hit” model by which Vps34 orchestrates eTreg fitness, wherein Vps34 coordinates both terminal eTreg generation during perinatal life and eTreg maintenance after perinatal life, as well as the underlying mechanisms (S7D Fig). Because mice bearing Vps34-deficient Tregs develop a fatal autoimmune-like syndrome, these findings suggest that the transitional heterogeneity of eTregs during perinatal life is critical for the establishment of immune tolerance. Therefore, engineering Tregs to acquire perinatal terminal eTreg-like fates may be beneficial for the treatment of autoimmunity, transplant rejection, or other inflammatory diseases [96]. Although pharmacological inhibitors of Vps34 are in development [97,98], our data suggest that those inhibitors selectively targeting the autophagy function of Vps34 may be more suitable to unleash anti-tumor immunity while minimizing the potential for systemic immune hyperactivation. Moreover, pharmacological inhibition of Vps34 complex I may decrease Treg accumulation in tumors via direct effects on Tregs [39] and indirect effects on tumor cells [99], thereby boosting therapeutic effects. Ultimately, the observations that distinct Vps34 complexes regulate eTreg transitional heterogeneity and functional adaptation may provide opportunities to selectively modulate Treg function in autoimmunity, cancer, and other diseases.

## Materials and methods

### Ethics statement

The research conducted in this study complied with all ethical regulations. Animal protocols were approved by the Institutional Animal Care and Use Committee (IACUC) of St. Jude Children’s Research Hospital (IACUC protocol 470–100586).

### Mice

*Rag1*^−/−^, CD45.1^+ ^, *Pik3c3*^tm1c(EUCOMM)Wtsi^, and *Rosa26*^YFP^ reporter (a *lox*P-site-flanked STOP cassette followed by the YFP-encoding sequence was inserted into the *Rosa26* locus) mice were purchased from the Jackson Laboratory. *Atg14*^fl/fl^ and *Uvrag*^fl/fl^ mice were obtained from T. Yoshimori [68] and T. Mak [67], respectively. *Foxp3*^Cre-YFP^ and *Foxp3*^GFP-Cre-ERT2^ mice were from A. Rudensky [37,46]. All genetic models were on the C57BL/6 background, with both male and female mice used for analysis and quantification. *Foxp3*^Cre-ERT2^*Pik3c3*^fl/fl^, *Foxp3*^Cre/+ ^*Pik3c3*^fl/fl^, *Foxp3*^Cre^*Atg14*^fl/fl^, and *Foxp3*^Cre^*Uvrag*^fl/fl^ mice were used at 6–20 weeks of age unless otherwise noted in the figure legends, with age- and sex-matched *Foxp3*^Cre-ERT2^ or *Foxp3*^Cre^-containing mice used as controls. Perinatal *Foxp3*^Cre^*Pik3c3*^fl/fl^ and *Foxp3*^Cre^*Atg14*^fl/fl^ mice were used at less than 12 days of age due to the development severe systemic inflammation, with age- and sex-matched *Foxp3*^Cre^-containing mice used as controls. For analysis of *Pik3c3* deletion, *Foxp3*^Cre-ERT2^*Pik3c3* mice were treated with tamoxifen (2 mg/mouse; prepared in corn oil) via intraperitoneal injection every 2 days for a total of six injections [38]. Mice were then euthanized 16 days after the first tamoxifen injection (16 dpi) or 30 days after the first tamoxifen injection (30 dpi). Tissues were harvested for flow cytometry analysis at both timepoints. Mixed BM chimeras were generated by transferring a total of 1 × 10^6^ (0.5 × 10^6^ BM cells from CD45.2^+^ control (i.e., *Foxp3*^Cre^*Pik3c3*^+/+ ^, *Foxp3*^Cre^*Pik3c3*^+ /fl^, *Foxp3*^Cre^*Atg14*^+/+ ^, or *Foxp3*^Cre^*Atg14*^+/+ ^; denoted as *Foxp3*^Cre^*Pik3c3*^+/+ or + /fl^ or *Foxp3*^Cre^*Atg14*^+/+ or +/fl^ in the text and figures), *Foxp3*^Cre^*Pik3c3*^fl/fl^, or *Foxp3*^Cre^*Atg14*^fl/fl^ mice mixed at a 1:1 ratio with 0.5 × 10^6^ BM cells from CD45.1^ +^ mice) T cell-depleted BM cells into sublethally irradiated (5.5 Gy) *Rag1*^−/−^ mice [21,24,38]. Of note, *Foxp3*^Cre^*Atg14*^fl/fl^ mice bearing an *Atg14*^+ /−^ or *Atg14*^−/−^ allele were analyzed in some experiments, and no notable differences between these mice and those bearing the *Atg14*^+ / +^ allele were observed for the reported phenotypes in this study. All mice were kept in a specific pathogen-free facility in the Animal Resource Center at St. Jude Children’s Research Hospital.

### Cellular isolation from lymphoid and non-lymphoid tissues

Single-cell suspensions were generated from spleen, PLNs, and PPs by gently grinding in HBSS (Life Technologies) containing 2% (vol/vol) fetal bovine serum (FBS). For liver and lung tissue, liver tissue was disrupted via gentle grinding and lung tissue was cut into ~2 mm pieces, and both tissues were digested in Roswell Park Memorial Institute (RPMI) 1640 medium (Life Technologies) containing 1-mg/mL Collagenase Type IV (Worthington Biochemical) for 1 h at 37 °C. The digested tissue was washed with HBSS and centrifuged for 1 min at 100*g* to remove debris (for liver). The supernatants from the liver and lung were passed through a sterile 70-μm nylon filter (Thermo Fisher Scientific). After centrifugation, the cell pellets from the liver tissue were resuspended in 80% Percoll (Life Technologies) underlaid beneath 40% Percoll, and lymphocytes were enriched by density centrifugation. Cells from the spleen, liver, and lung were additionally lysed with ACK lysis buffer (Gibco) to remove red blood cells prior to experimental use.

Colon LP isolation was performed as described with modification [18]. Briefly, colon tissue that was longitudinally dissected and cut into ~2 mm pieces was incubated with RPMI 1640 medium containing 2% (vol/vol) FBS, 25 mM HEPES (Gibco), 1% (vol/vol) penicillin–streptomycin–L-glutamine, 0.5 mM EDTA (Invitrogen), and 0.1 mM DTT for 20 min at 37 °C with gentle shaking (225 rpm). The tissue was then washed twice with RPMI 1640 containing 2% (vol/vol) FBS, 1% (vol/vol) penicillin–streptomycin–l-glutamine, 25 mM HEPES, and 0.2 mM EDTA for 10 min at 37 °C with gentle shaking to remove cells in the epithelial layer. To isolate the LP fraction, the colon tissue was digested with RPMI 1640 containing 25 mM HEPES, 1% (vol/vol) penicillin–streptomycin–l-glutamine, and 0.1 mg/ml Liberase TL (Roche) for 30 min at 37 °C with gentle shaking. The cells were washed with RPMI 1640 medium containing 25 mM HEPES, 1% (vol/vol) penicillin–streptomycin–l-glutamine, and 3% FBS. To enrich for lymphocytes from the colon LP, cells were resuspended in 40% Percoll overlaid onto 80% Percoll and enriched by density centrifugation at 1,000*g* for 20 min. All cell samples were counted using an automated cell counter (Nexcelom Biosciences) prior to experimental use.

### Flow cytometry

For analysis of surface markers, cells were stained in PBS containing 1% (wt/vol) bovine serum albumin (BSA) for 30 min on ice with anti-CD4 (RM4-5; 1:200), anti-CD8α (53-6.7; 1:300), anti-TCRβ (H57-597; 1:200), anti-CD45 (Ly-5) (30-F11; 1:200), anti-CD44 (IM7; 1:200 or 1:400), anti-CD62L (MEL-14; 1:300 or 1:1,000), anti-CD45.1 (A20; 1:200), anti-CD45.2 (104; 1:100), anti-CD25 (PC61.5; 1:200), anti-CD278 (ICOS) (7E.17G9 or C398.4A; 1:100), anti-CD73 (eBioTY/11.8; 1:200), anti-CD357 (AITR/GITR) (DTA-1; 1:200), anti-CD279 (PD-1) (J43 or 2GF.1A12; 1:200), and anti-KLRG1 (2F1/KLRG1; 1:200) (Tonbo Biosciences, eBioscience, or Biolegend). Surface staining for IL-33R (ST2) was performed using biotinylated anti-IL-33R (RMST2-33; 1:200) for 45 min on ice followed by fluorescently conjugated streptavidin (1:500) for 30 min on ice (eBioscience). Intracellular Foxp3 (FJK-16s; 1:200), GFP (FM264G; 1:200), Ki67 (16A8, B56, or SolA15; 1:400), TIGIT (GIGD7; 1:100), CD152 (CTLA-4) (UC10-4F10-11 or UC10-4B9; 1:200), IFN-γ (XMG1.2; 1:400), IL-4 (11B11; 1:200), and IL-17A (TC11-18H10.1; 1:200) (Tonbo Biosciences, eBioscience, BioLegend, or Cell Signaling Technology) were stained for 30 min on ice after fixation and permeabilization with the Foxp3/transcription factor staining buffer kit according to the manufacturer’s instructions (eBioscience, cat no. 00-5521-00). Intracellular Bcl2 (BCL/10C4; 1:200), Bim (C34C5; 1:200), and Mcl1 (LVUBKM; 1:200) (eBioscience, BioLegend, or Cell Signaling Technology) were stained for 30 min on ice after fixation and permeabilization with the Cytofix/Cytoperm kit according to the manufacturer’s instructions (BD Biosciences, cat no. 554714). For intracellular cytokine staining, splenocytes containing T cells were stimulated for 4 h with PMA and ionomycin in the presence of GolgiStop Protein Transport Inhibitor before intracellular staining according to the manufacturer’s instructions (BD Biosciences, cat no. 554724). For cell survival analysis, lymphocytes were stained with Fixable Viability Dye Aqua (FVD; 1:1,000) or 7-amino-actinomycin D (7-AAD; 1:1,000) per the manufacturer’s instructions (eBioscience, cat no. 65-0865-14 and 006993−50, respectively). For mitochondrial staining, lymphocytes were incubated for 30 min at 37 °C with 10-nM MitoTracker Deep Red (Life Technologies, cat no. M22426), 20-nM TMRM (Life Technologies, cat no. T668), 10-nM CellROX Deep Red (Life Technologies, cat no. C10422), or 5-μM MitoSOX Red (Life Technologies, cat no. M36008) after staining surface markers [22,100]. Flow cytometry data were acquired on a Symphony A3, LSR Fortessa, or LSRII flow cytometer (BD Biosciences) and analyzed using FlowJo software (version 10.10.0; Tree Star). See also S15 Table for more information about flow cytometry reagents used in this study. Gating strategies used in this study are shown in S1 File.

### Histology

All tissues were fixed in 10% (vol/vol) neutral buffered formalin, embedded in paraffin, sectioned, and stained with H&E using standard methods. All sections were examined and scored for leukocyte infiltration and inflammation by an experienced pathologist (P. Vogel).

### Cell purification and culture

Unless otherwise noted, lymphocytes were isolated from spleen and PLNs (including inguinal, auxiliary, and cervical LNs) and enriched for CD4^ +^ T cells via positive selection using magnetic CD4 (L3T4) MicroBeads (Miltenyi Biotec). Enriched samples were sort-purified for cTregs (CD4^+ ^Foxp3-YFP^+^CD44^low^CD62L^high^) from the *Foxp3*^Cre^-expressing mouse strains (as indicated in the figures and their legends) on a MoFlo (Beckman-Coulter) or Bigfoot (Life Technologies) cell sorter. Sort-purified cTregs were cultured in plates coated with anti-CD3 (145-2C11, 5 μg/ml) and anti-CD28 (37.51; 5 μg/ml; both from Bio X Cell) antibodies for 72 h in Click’s medium supplemented with β-mercaptoethanol, 10% (vol/vol) FBS, 1% (vol/vol) penicillin–streptomycin–l-glutamine, and IL-2 (100 U/ml) (hereafter described as complete Click’s medium) [24,43].

### RNA isolation and real-time quantitative PCR

RNA was isolated using the RNeasy Micro Kit (Qiagen, cat no. 74004) following the manufacturer’s instructions. RNA was converted to cDNA using the High-Capacity cDNA Reverse Transcription Kit (Thermo Fisher Scientific, cat. no 4368813) according to the manufacturer’s instructions. Quantitative PCR analysis was performed on the QuantStudio 7 Flex System (Applied Biosystems) using the following probes: *Uvrag* (Mm 01273471) and *Actb* (Mm 00607939_s1). The primers for SYBR Green master mix were *Pik3c3* (forward: 5′- CCGTGTCGCTCTTTGGAAAA -3′, reverse: 5′- TCTGCCGGGAGTTCTTGTAG -3′), *Atg14* (forward: 5′- TGCAAGATGAGGATTGAACAGC -3′, reverse: 5′- GTTGTGCCGCTGAATCTTCT-3′), and *Actb* (forward: 5′- TCCGGCACTACCGAGTTATC -3′, reverse: 5′- GATCCGGTGTAGCAGATCGC -3′).

### Tumor model and tumor-infiltrating lymphocyte isolation

MC38 colon adenocarcinoma cells were provided by D. Vignali, and B16F10 melanoma cells were obtained from ATCC. Both cell lines were maintained in the laboratory and cultured in RPMI 1640 medium supplemented with 10% (vol/vol) FBS and 1% (vol/vol) penicillin–streptomycin. Mice were injected subcutaneously with 5 × 10^5^ MC38 colon adenocarcinoma cells or 2.5 × 10^5^ B16F10 melanoma cells in the right flank [22,100]. Tumors were measured every other day beginning on day 7 (MC38) or day 11 (B16F10) after tumor inoculation using digital calipers, and tumor volumes were calculated using the following formula: Length ×  Width ×  [(Length ×  Width)^0.5^] ×  *π*/6. Tamoxifen (2 mg/mouse; prepared in corn oil) was intraperitoneally injected into control (i.e., *Foxp3*^Cre-ERT2^*Pik3c3*^+ / +^ or *Foxp3*^Cre-ERT2^*Pik3c3*^+ /fl^ mice) or *Foxp3*^Cre-ERT2^*Pik3c3*^fl/fl^ mice daily from days 7 to 11 after tumor inoculation to induce *Pik3c3* gene deletion during early tumorigenesis [22]. To prepare tumor-infiltrating lymphocytes (TILs) [22,100], tumors were excised, minced, and digested with 1-mg/ml Collagenase IV (Roche) +  200 U/ml DNase I (Sigma) for 1 h at 37 °C. TILs were isolated by density-gradient centrifugation over Percoll (Life Technologies). Cells were prepared for flow cytometry analysis as described above.

### Gene expression profiling

For transcriptome profiling via microarray analysis, total Tregs (CD4^+ ^Foxp3-YFP^+^) were sort-purified from control *Foxp3*^Cre^*Pik3c3*^+ /fl^ (*n* =  4) or *Foxp3*^Cre^*Pik3c3*^fl/fl^ (*n* =  5) mixed BM chimeras. Alternatively, cTregs (CD45.2^+ ^CD4^+ ^Foxp3-YFP^+^CD44^low^CD62L^high^) and eTregs (CD45.2^+ ^CD4^+ ^Foxp3-YFP^+^CD44^high^CD62L^low^) were sort-purified from control *Foxp3*^Cre^*Atg14*^+ /fl^ (*n* =  4 for cTregs; 4 for eTregs) or *Foxp3*^Cre^*Atg14*^fl/fl^ (*n* =  3 for cTregs; 3 for eTregs) mixed BM chimeras. RNA samples were isolated from CD45.2^ +^ control and Vps34- or Atg14*-*deficient Tregs sort-purified from the spleen of *Foxp3*^Cre^*Pik3c3* or *Foxp3*^Cre^*Atg14* mixed BM chimeras and analyzed with the Clariom S mouse array. The expression signals were summarized using the robust multi-array average algorithm Affymetrix Expression Console version 1.1. All plots were generated using the R package ggplot2 (version 3.5.1). Differential expression analysis of genes was performed using the lmFit method implemented in the R package limma (version 3.60.5), and the Benjamini–Hochberg method was used to estimate the false discovery rate (FDR) as described [21]. Differentially expressed genes were defined by |log_2_ fold change (FC)| ≥  0.5 and adjusted *P* (FDR) <  0.05 or *P* <  0.05 as indicated in figure legends and tables.

### Gene signature curation and gene set enrichment analysis

In-house gene signatures were generated as follows: (1) The Vps34-activated Treg signature was defined based on the top 200 downregulated genes (log_2_FC < −0.5 and *P* <  0.05) in Vps34-deficient total Tregs compared to control Tregs (from the microarray analysis from mixed BM chimeras; S2 Table). (2) The Vps34-suppressed Treg signature was defined based on the top 200 upregulated genes (log_2_FC >  0.5 and *P* <  0.05) in Vps34-deficient total Tregs compared to control Tregs (from the microarray analysis from mixed BM chimeras; S2 Table). (3) The Vps34-activated eTreg signature was defined based on the downregulated genes (log_2_FC ≤  −0.5 and FDR <  0.05) in total Vps34-deficient eTregs (transitional +  terminal eTreg) compared to control total eTreg (transitional +  terminal eTreg) (from the merged single-cell transcriptomics data from perinatal mice; S14 Table). (4) The Atg14-activated eTreg signature was defined based on the downregulated genes (log_2_FC ≤  −0.5 and *P* <  0.05) in Atg14-deficient eTregs compared to control eTreg (from the microarray analysis from mixed BM chimeras; S14 Table).

The following signatures were derived from publicly available gene sets: (1) The cTreg signature (top 200 upregulated genes in cTregs compared to eTregs; log_2_FC >  0.5 and FDR <  0.05) and eTreg signature (top 200 upregulated genes in eTregs compared to cTregs; log_2_FC >  0.5 and FDR <  0.05) were generated using GSE61077 [5]. (2) KLRG1^−^Nfil3^−^ cTreg signature (top 200 upregulated genes ranked by log_2_FC; log_2_FC >  1 and FDR <  0.05 in KLRG1^−^Nfil3^−^ Tregs versus others [combination of KLRG1^−^Nfil3^ +^ Tregs and KLRG1^+ ^Nfil3^ +^ Tregs]); (3) KLRG1^−^Nfil3^ +^ transitional Treg signature (top 200 upregulated genes ranked by log_2_FC; log_2_FC >  0.5 and FDR <  0.05 in KLRG1^−^Nfil3^ +^ Tregs versus others [combination of KLRG1^−^Nfil3^−^ Tregs and KLRG1^+ ^Nfil3^ +^ Tregs]); and (4) KLRG1^+ ^Nfil3^ +^ terminal Treg signature (top 200 upregulated genes ranked by log_2_FC; log_2_FC >  1 and FDR <  0.05 in KLRG1^+ ^Nfil3^ +^ Tregs versus others [combination of KLRG1^−^Nfil3^ +^ Tregs and KLRG1^−^Nfil3^−^ Tregs]). The KLRG1^−^Nfil3^−^ cTreg, KLRG1^−^Nfil3^ +^ transitional Treg, and KLRG1^+ ^Nfil3^ +^ terminal Treg signatures were generated using GSE130842 [11].

For the analysis of Vps34-deficient Tregs compared to control Tregs (microarray), GSEA was performed as described previously [21] using the above cTreg and eTreg signatures together with gene sets in the Hallmark collections of the Molecular Signatures Database (MSigDB). For analysis of Atg14-deficient eTregs compared to control eTregs (microarray), GSEA was performed using the gene sets in the Hallmark collections of MSigDB. For the analysis of KLRG1^+ ^Nfil3^ +^ terminal Tregs compared to KLRG1^−^Nfil3^ +^ transitional Tregs (from a public dataset [11]), GSEA was performed using the Vps34-activated Treg and Vps34-suppressed Treg signatures together with gene sets in the Hallmark collections of MSigDB. Finally, the activity scores of the Vps34-activated eTreg signature and Atg14-activated eTreg signature were applied to publicly available scRNA-seq datasets of human intratumoral Tregs from HNSCC (GSE139324 [69]), breast cancer (GSE114727 [70]), melanoma (GSE239750 [71]), and SCLC (GSE239750 [71]). The activity scores were plotted on violin plots, using the Vln.plot function in Seurat (v5.1.0).

### ATAC-seq analysis

#### Bulk ATAC-seq library preparation.

Total Tregs (TCRβ^+^CD4^+ ^Foxp3-YFP^+^) were sort-purified from the spleen (*n* =  4 for control and 2 for *Foxp3*^Cre^*Pik3c3*^fl/fl^) and lungs (*n* =  2 per genotype) of 10-day-old perinatal control *Foxp3*^Cre^*Pik3c3*^+ / + or + /fl^ and *Foxp3*^Cre^*Pik3c3*^fl/fl^ mice. Alternatively, cTregs (CD45.2^+ ^CD4^+ ^Foxp3-YFP^+^CD44^low^CD62L^high^) and eTregs (CD45.2^+ ^CD4^+ ^Foxp3-YFP^+^CD44^high^CD62L^low^) were sort-purified from control *Foxp3*^Cre^*Atg14*^+ /fl^ (*n* =  3 for cTregs; 3 for eTregs) or *Foxp3*^Cre^*Atg14*^fl/fl^ (*n* =  4 for cTregs; 4 for eTregs) mixed BM chimeras. ATAC-seq samples were prepared as previously described [63]. Briefly, cells were lysed in 50-μl lysis buffer (10 mM Tris-HCl, pH 7.4, 10 mM NaCl, 3 mM MgCl_2_, 0.1% IGEPAL CA-630) on ice for 10 min followed by centrifugation (500 *g*, 10 min, 4 °C). The supernatant was carefully discarded by pipette. The pelleted nuclei were resuspended in 50-μl transposase reaction mix (25-μl 2× TD buffer, 22.5-μl nuclease-free water, and 2.5-μl Transposase enzyme) and then incubated for 30 min at 37 °C. DNA from the Transposase reaction was cleaned up using the Qiagen MinElute kit. The barcoding reaction was amplified for 5 cycles according to the manufacturer’s instructions (Illumina) using the NEBNext HiFi kit and verified barcoding primers, and 5 μl of the barcoding reaction was used to determine the ideal remaining cycle number using KAPA SYBRFast (Kapa Biosystems) reagents and real-time PCR (Applied Biosystems 7900HT). The remaining 45 μl of sample was amplified in the same reaction mix using the optimal cycle number.

#### ATAC-seq data analysis.

ATAC-seq data were analyzed as described [63]. In brief, 2 × 100-bp paired-end reads from samples were trimmed for Nextera adaptor by trimmomatic (version 0.36) (with parameters ILLUMINACLIP:adaptor.fa:2:30:10 LEADING:10 TRAILING:10 SLIDINGWINDOW:4:18 MINLEN:25) and then aligned to the mouse genome mm9 (downloaded from gencode release M1, https://www.gencodegenes.org/mouse/releases.html) by BWA (version 0.7.16a, with default parameters) [101]. Duplicated reads were labeled with Picard (version 2.9.4), and only non-duplicated paired reads were analyzed further as determined by samtools (parameter “-q 1 -F 1804” version 1.9) [102]. With the adjustment of Tn5 shift (reads were offset by +4 bp for the sense strand and −5 bp for the antisense strand), the reads were separated into nucleosome-free, mono-, di-, and trinucleosomes by fragment size. A file of bigwig was generated by using the central fragment of 80-bp and then scaled to 30 × 10^6^ nucleosome-free reads. Several peaks, including nucleosome-free, mono-, di-, and tri-nucleosomes, were observed on IGV (version 2.18.4) [103]. Samples from each group had about 20 × 10^6^ nucleosome-free reads on average. MACS2 was used for peak calling on each sample (with default parameters and with “–extsize 200–nomodel”) [104]. To ensure reproducibility, nucleosome-free regions of each genotype were re-finalized. Peaks from each replicate were kept if they were present in at least 50% of the replicates, followed by merging of all the peaks if they overlapped by 100-bp. Nucleosome-free reads from each of the samples were calculated by the bedtools package (version 2.25.0) [105].

To find the differentially accessible regions, raw nucleosome-free read counts were normalized by the counts per million (CPM) method implemented in DEseq2 (version 1.44.0). FDR-corrected *P*-value <  0.05 and log_2_FC >  0.5 were used as cutoffs for more-accessible or less-accessible regions in *Foxp3*^Cre^*Pik3c3*^fl/fl^ samples. The differentially accessible regions in ATAC-seq data were annotated as the nearest genes using Homer annoatePeaks.pl (version 4.9.1). PSEA was performed using published or curated signatures from public transcriptomics datasets [11] of different Treg populations as follows: (1) KLRG1^−^Nfil3^−^ cTreg signature (top 200 upregulated genes ranked by log_2_FC; log_2_FC >  1 and FDR <  0.05 in KLRG1^−^Nfil3^−^ Tregs versus others [combination of KLRG1^−^Nfil3^ +^ Tregs and KLRG1^+ ^Nfil3^ +^ Tregs]); (2) KLRG1^−^Nfil3^ +^ transitional Treg signature (top 200 upregulated genes ranked by log_2_FC; log_2_FC >  0.5 and FDR <  0.05 in KLRG1^−^Nfil3^ +^ Tregs versus others [combination of KLRG1^−^Nfil3^−^ Tregs and KLRG1^+ ^Nfil3^ +^ Tregs]); (3) KLRG1^+ ^Nfil3^ +^ terminal Treg signature (top 200 upregulated genes ranked by log_2_FC; log_2_FC >  1 and FDR <  0.05 in KLRG1^+ ^Nfil3^ +^ Tregs versus others [combination of KLRG1^−^Nfil3^ +^ Tregs and KLRG1^−^Nfil3^−^ Tregs]); (4) Fat Treg versus spleen Treg signature (top 200 upregulated genes ranked by log_2_FC; log_2_FC >  1 and FDR <  0.05); (5) Liver Treg versus spleen Treg signature (top 200 upregulated genes ranked by log_2_FC; log_2_FC >  1 and FDR <  0.05); (6) Core tissue Treg signature (represents shared differentially accessible genes in fat, liver, lung [top 200 upregulated genes ranked by log_2_FC; log_2_FC >  1 and FDR <  0.05], and skin [top 200 upregulated genes ranked by log_2_FC; log_2_FC >  1 and FDR <  0.05] Tregs compared to spleen Tregs).

For motif enrichment analysis, the regions with CPM greater than the first quartile of all CPM and least variable according to their median absolute deviation score and FDR *P*-value >  0.5 were used as control regions. FIMO from MEME suite (version 4.11.3, “–thresh 1e-4–motif-pseudo 0.0001”) was selected for scanning motif (TRANSFAC database, only included vertebrata and not 3D structure-based) matched to the nucleosome-free regions [106]. The significantly enriched motifs were determined by comparing differentially accessible regions to the control regions using two-tailed Fisher’s exact test. Footprinting of transcription factor binding sites was performed using the methods and regulatory genome tools box as previously described [63,100].

#### Single cell RNA-seq and single-cell multiome profiling.

For 3′ scRNA-seq analysis, total Tregs (TCRβ^+^CD4^+ ^Foxp3-YFP^+^) were sort-purified from the spleen of 10-day-old control *Foxp3*^Cre^*Pik3c3*^+ / +^ (*n* =  1) and *Foxp3*^Cre^*Pik3c3*^fl/fl^ (*n* =  1) mice. All samples were centrifuged at 2,000 rpm for 5 min. The supernatant was removed, and cells were re-suspended in 100 μl of 1× PBS (Thermo Fisher Scientific) +  0.04% BSA (Amresco). The cells were then counted and checked for viability (>98%) using a Luna Dual Florescence Cell Counter (Logos Biosystems). Single cell suspensions were loaded onto the Chromium Controller according to their respective cell counts to generate single cell GEMs (gel beads in emulsion) per the manufacturer’s protocol. Each sample was loaded into a separate channel. Libraries were prepared using the Chromium Single Cell 3′ v2 Library and Gel Bead Kit (10× Genomics). The cDNA content of each sample after cDNA amplification of 12 cycles was quantified and quality checked using a High-Sensitivity DNA chip with a 2100 Bioanalyzer (Agilent Technologies) to determine the number of PCR amplification cycles to yield sufficient library for sequencing. After library quantification and quality check by DNA 1000 chip (Agilent Technologies), samples were diluted to 3.5 nM for loading onto the HiSeq 4000 (Illumina) with a 2 × 75 paired-end kit using the following read length: 26 bp Read1, 8 bp i7 Index, and 98 bp Read2. An average of 400,000,000 reads per sample was obtained (~100,000 reads per cell).

For multiome analysis, total Tregs (TCRβ^+^CD4^+ ^Foxp3-YFP^+^) were sort-purified from the spleen of 12-day-old control *Foxp3*^Cre^*Pik3c3*^+ /fl^ (*n* =  1) and *Foxp3*^Cre^*Pik3c3*^fl/fl^ (*n* =  2) mice. All samples were centrifuged at 2,000 rpm for 5 min at 4 °C. The supernatant was removed, and the pellets were resuspended in 50 μl 1× PBS plus 0.04% BSA. Nuclei for Single Cell Multiome ATAC +  Gene Expression Sequencing were isolated, washed, and counted per the manufacturer’s instructions (10× Genomics). Single nuclei suspensions were loaded onto the Chromium Controller according to their respective cell counts to generate single nuclei GEMs per the manufacturer’s protocol. Each sample was loaded into a separate channel. Libraries were prepared using the Chromium Next GEM Single Cell Multiome ATAC +  Gene Expression Reagent Bundle (10× Genomics). The cDNA content of each sample was amplified (7 cycles for ATAC library, 12 cycles for Gene Expression library), and then quantified and quality checked using a High Sensitivity DNA chip with a TapeStation 4200 analyzer (Agilent Technologies). Samples for ATAC library sequencing were diluted to 150 pM for loading onto the NovaSeq (Illumina) with a 2 × 100 paired-end kit using the following read cycles: 50 cycles Read 1N, 8 cycles i7 Index, 24 cycles i5 Index, and 49 cycles Read 2N. An average of 310,000,000 reads per sample was obtained (~49,063 read pairs per nucleus). Samples for Gene Expression library sequencing were diluted to 150 pM for loading onto the NovaSeq (Illumina) with a 2 × 100 paired-end kit using the following read cycles: 28 cycles Read 1, 10 cycles i7 Index, 10 cycles i5 Index, and 90 cycles Read 2N. An average of 470,000,000 reads per sample was obtained (~73,736 read pairs per nucleus).

### Analysis for single-cell transcriptome (scRNA-seq combined with snRNA-seq) data

#### Alignment, barcode assignment, and UMI counting.

For scRNA-seq analysis, the Cell Ranger 2.1.1 Single-Cell Software Suite (10× Genomics) was implemented to process the raw sequencing data from the Illumina HiSeq run. This pipeline performed de-multiplexing, alignment (mm10), and barcode processing to generate gene-cell matrices used for downstream analysis. Cells with low (potentially dead cells with broken membrane) or high (potentially two or more cells in a single droplet) unique molecular identifier (UMI) counts were filtered. A total of 6,464 cells (control: 3,525; *Foxp3*^Cre^*Pik3c3*^fl/fl^: 2,939) were captured with an average of 6,740 mRNA molecules (UMIs, median: 5,672, range: 2,736–19,956). The expression level of each gene was normalized using the NormalizeData function with a scale factor of 10^6^.

For snRNA-seq analysis from multiome profiling, the Cell Ranger ARC 2.0.0 Single-Cell Software Suite (10× Genomics) was implemented to process the raw sequencing data from the Illumina HiSeq run. This pipeline performed de-multiplexing, alignment (mm10), and barcode processing to generate gene-cell and peak-cell matrices used for downstream gene expression and ATAC analysis, respectively. Cells with low or high UMI counts were filtered. A total of 17,039 cells (control: 6,084; *Foxp3*^Cre^*Pik3c3*^fl/fl^ replicate 1: 4,125; *Foxp3*^Cre^*Pik3c3*^fl/fl^ replicate 2: 6,830) were captured with an average of 3,231 mRNA molecules (UMIs, median: 2,870, range: 270–16,898). The expression level of each gene was normalized using the NormalizeData function with a scale factor of 10^6^.

#### Integration of scRNA-seq and snRNA-seq datasets.

scRNA-seq and snRNA-seq Treg datasets were integrated using the FindIntegrationAnchors (with reduction as “cca”) and IntegrateData functions in the Seurat R package, followed by clustering with the default assay set as “integrated”. The integrated scRNA-seq and snRNA-seq dataset was processed with the ScaleData, RunPCA, FindNeighbors, and FindClusters functions using default parameters for unbiased clustering. cTreg (*Sell*^+^*Cd44*^−^), transitional eTregs (*Sell*^−^*Cd44*^+ ^*Klrg1*^−^), and terminal eTregs (*Sell*^−^*Cd44*^+ ^*Klrg1*^+^) were annotated by their markers.

### Data visualization

Our unbiased clustering analysis was mapped into 2D space using UMAP. Expression of genes corresponding with specific Treg states was color-coded (red: cTregs; green: transitional eTregs; blue: terminal eTregs) (see Fig 3A). Transcriptomic profiles of control (red) and *Foxp3*^Cre^*Pik3c3*^fl/fl^ (blue) mice were overlapped to visualize differences in gene expression that occur in the absence of Vps34 (see Fig 3D). Pseudotime trajectory analysis was performed using the Slingshot (v.2.12.0) R package with default settings. Activity scores of gene signatures were calculated using the AddModuleScore function of the Seurat package. Raw and processed scRNA-seq and multiomics data have been deposited into the GEO series database under accession GSE279606.

### scATAC-seq (from multiome profiling) analysis

#### Merge samples in scATAC-seq.

ATAC peaks from each sample were merged to create a common peak set and quantified by Signac R package (version 1.14.0). Specifically, peak coordinates for each sample were loaded and converted to genomic ranges. The GenomicRanges::reduce function was used to first create a common set of peaks. Fragment objects were created using the CreateFragmentObject function for each sample to quantify peaks using the FeatureMatrix function. Quantified matrices were used to create a Seurat object for each sample, and the standard merge function was then used to merge the objects. Normalization and linear dimensional reduction were performed on the merged scATAC-seq dataset through latent semantic indexing using RUNTFIDF, FindTopFeatures, and RunSVD functions with default parameters. The scATAC-seq dataset was then integrated with snRNA-seq data through the corresponding cell barcode for each cell. Using the weighted nearest neighbor methods in the Seurat R package, a joint neighbor graph that represents both the gene expression and DNA accessibility measurements was computed by FindMultiModalNeighbors function. cTregs (*Sell*^+^*Cd44*^−^), transitional eTregs (*Sell*^−^*Cd44*^+ ^*Klrg1*^−^), and terminal eTregs (*Sell*^−^*Cd44*^+ ^*Klrg1*^+^) were annotated based on their marker expression from snRNA-seq data on the UMAP.

#### Differential accessibility, motif enrichment analysis, and PSEA.

Differentially accessible peaks between cell types or genotypes were calculated by the FindMarkers function with min.pct =  0.1. Transcription factor motifs were identified using the FindMotifs function based on the differentially upregulated and downregulated peaks. Additionally, we also computed a per-cell motif activity score by running chromVAR (runChromVAR function) in the Signac R package (version 1.14.0) and directly tested for differential activity scores between genotypes in the indicated Treg cell states using the FindMarkers function. For PSEA, differentially upregulated and downregulated peaks in each comparison were filtered based on the indicated thresholds in each figure. Genes closest to the peak region were annotated using the ClosestFeature function in the Signac R package. Functional enrichment analysis of the genes was then performed using the funcEnrich.Fisher function in the NetBID2 R package (version 2.0.3) and the custom gene sets indicated above.

### Statistical analysis

Prism 10 software (GraphPad, version 10.3.1) was used to analyze all non-omics data by performing two-tailed unpaired Student *t* test. Two-way ANOVA was used to compare tumor growth curves. The Mantel–Cox test was used for comparing mouse survival curves. Two-tailed Wilcoxon rank sum test was applied for differential expression or activity score analysis of scRNA-seq data. Two-tailed Kolmogorov–Smirnov test by GSEA was used for pathway activity score analysis of scRNA-seq data. Two-tailed unpaired Student *t* test was used for transcription factor footprinting analysis of ATAC-seq peaks. A *P*-value ≤  0.05 was considered significant, with *P-*values <  0.1 considered trending. Data are presented as mean ±  s.e.m.

## Supporting information

S1 Fig Loss of Vps34 is associated with decreased eTreg survival but intact activation. (**A**) Quantitative PCR analysis for *Pik3c3* deletion efficiency in TCRβ^+^CD4^+ ^Foxp3-YFP^ +^ Tregs that were sort-purified (pooled from spleen and PLNs) from 17- to 42-day-old control (*n* =  4) or *Foxp3*^Cre^*Pik3c3*^fl/fl^ (*n* =  2) mice. PLNs, peripheral lymph nodes. (**B**) Quantification of frequency (left) and number (right) of total TCRβ^+^CD4^+ ^Foxp3-YFP^+^ Tregs derived from the spleen of 20- to 30-day-old control (*n* =  7) or *Foxp3*^Cre^*Pik3c3*^fl/fl^ (*n* =  4) mice. (**C**) Flow cytometry analysis (left) and quantification (right) of frequencies and numbers of total TCRβ^+^CD4^+ ^Foxp3-YFP^ +^ Tregs derived from the spleen of control mosaic (*n* =  13) or *Foxp3*^Cre/+ ^*Pik3c3*^fl/fl^ mosaic (*n* =  15) mice. (**D**) Quantification of the frequency (left) and number (right) of total TCRβ^+^CD4^+ ^Foxp3-YFP^ +^ Tregs derived from the lung of control mosaic or *Foxp3*^Cre/+ ^*Pik3c3*^fl/fl^ mosaic mice (*n* =  6 per group). (**E**) CD4^+ ^Foxp3-YFP^ +^ Tregs (CD45.2^+^) were sort-purified from the spleen of control (*n* =  4) or *Foxp3*^Cre^*Pik3c3*^fl/fl^ (*n* =  5) mixed BM chimera mice and profiled by microarray analysis (see Materials and methods for details). Volcano plot depicting upregulated (red) and downregulated (blue) genes, with select eTreg-associated genes labeled. See also S1 Table. BM, bone marrow. (**F**) Quantification of frequencies (left) and numbers (right) of CD44^low^CD62L^high^ cTregs or CD44^high^CD62L^low^ eTregs among total TCRβ^+^CD4^+ ^Foxp3-YFP^ +^ Tregs derived from the spleen of 20- to 30-day-old control (*n* =  7) or *Foxp3*^Cre^*Pik3c3*^fl/fl^ (*n* =  4) mice. (**G**) Quantification of frequencies (left) and numbers (right) of CD44^low^CD62L^high^ cTregs or CD44^high^CD62L^low^ eTregs among total TCRβ^+^CD4^+ ^Foxp3-YFP^ +^ Tregs derived from the spleen of control mosaic (*n* =  13) or *Foxp3*^Cre/+ ^*Pik3c3*^fl/fl^ mosaic (*n* =  15) mice. (**H**) Quantification of relative (normalized to average control in each experiment) gMFIs of Foxp3 and CD25 in CD44^low^CD62L^high^ cTregs and CD44^high^CD62L^low^ eTregs among total TCRβ^+^CD4^+ ^Foxp3^+^ (Foxp3) or TCRβ^+^CD4^+ ^Foxp3-YFP^+^ (CD25) Tregs (all pre-gated on CD45.2^ +^ cells) derived from the spleen of control (*n* =  22 for Foxp3, 17 for CD25) or *Foxp3*^Cre^*Pik3c3*^fl/fl^ (*n* =  20 for Foxp3, 15 for CD25) mixed BM chimera mice, as determined by flow cytometry analysis. gMFI, geometric mean fluorescence intensity. (**I**) Quantification of the frequencies of Ki67^+^ cells among total TCRβ^+^CD4^+ ^Foxp3^ +^ Tregs, CD4^+ ^Foxp3^+ ^CD44^low^CD62L^high^ cTregs, or CD4^+ ^Foxp3^+ ^CD44^high^CD62L^low^ eTregs (all pre-gated on CD45.2^+^ cells) derived from the spleen of control (*n* =  15) or *Foxp3*^Cre^*Pik3c3*^fl/fl^ (*n* =  13) mixed BM chimera mice. (**J**) Spleen and PLNs of control mosaic or *Foxp3*^Cre/+ ^*Pik3c3*^fl/fl^ mosaic mice were pooled, and TCRβ^+^CD4^+ ^Foxp3-YFP^+^CD44^low^CD62L^high^ cTregs were sort-purified and cultured in vitro in the presence of anti-CD3, anti-CD28, and IL-2 for 72 h. Quantification of the frequency (relative to the average of technical replicates in each experiment) of CD44^high^CD62L^low^ eTreg-like cells (*n* =  4 biological replicates for control mosaic mice and 6 biological replicates for *Foxp3*^Cre/+ ^*Pik3c3*^fl/fl^ mosaic mice). (**K**) Quantification of relative (normalized to average control in each experiment) frequencies of FVD^ +^ non-viable CD44^low^CD62L^high^ cTregs or CD44^high^CD62L^low^ eTregs among total TCRβ^+^CD4^+ ^YFP^+^Foxp3^ +^ Tregs derived from the spleen of control mosaic or *Foxp3*^Cre/+ ^*Pik3c3*^fl/fl^ mosaic mice (*n* =  6 per group). FVD, fixable viability dye. Data are shown as mean ±  s.e.m. (**A**–**D**, **F**–**K**). Two-tailed Student *t* test (**A**–**D**, **F**–**K)** or Wald test (**E**); NS, not significant. Data are compiled from 1 (**A**), 3 (**B**, **F**, **J**), 8 (**C**, **G**), 4 (**D**, **K**), or ≥5 (**H**, **I**) independent experiments. Numbers in plots indicate percentages of cells in gates (**C**). The numerical data underlying the graphs shown in this figure are found in S8 Data (**A**–**D, F**–**K**). Raw microarray data used for analysis in (**E**) have been deposited to GEO SuperSeries access number GSE279606.
(TIFF)

S2 Fig Loss of Vps34 reduces Treg suppressive function and is associated with the development of type-I-dominant inflammatory disease. (**A**) Quantitative PCR analysis for deletion efficiency of *Pik3c3* in TCRβ^+^CD4^+ ^GFP^+^YFP^+^ Tregs that were sort-purified (pooled from spleen and PLNs) from control or *Foxp3*^Cre^*Pik3c3*^fl/fl^ (*n* =  2 per group) mice 16 days after beginning tamoxifen treatment (16 dpi). PLNs, peripheral lymph nodes; dpi, days post-injection. (**B**) Flow cytometry analysis (left) and quantification (right) of frequencies and numbers of CD44^high^CD62L^low^ effector/memory cells among TCRβ^+^CD4^+ ^Foxp3-YFP^−^ (denoted as CD4^+ ^Foxp3^−^) and TCRβ^+^CD8^+^ T cells derived from the spleen of 16- to 23-day-old control (*n* =  7) or *Foxp3*^Cre^*Pik3c3*^fl/fl^ (*n* =  4) mice. (**C**) Splenocytes were stimulated with PMA and ionomycin in the presence of GolgiStop for 4 h. Flow cytometry analysis (left) and quantification (right) of the frequencies of IFN-γ^+^, IL-4^+^, or IL-17A^+^ cells among ΤCRβ^+^CD4^+ ^Foxp3^ − ^CD44^high^ conventional Τ cells and IFN-γ^+^ cells among ΤCRβ^+^CD8^+ ^CD44^high^ T cells from 20- to 30-day-old control or *Foxp3*^Cre^*Pik3c3*^fl/fl^ mice (*n* =  8 per group). (**D**) Representative images of 26-day-old control and *Foxp3*^Cre^*Pik3c3*^fl/fl^ mice. Arrows indicate sites of focal dermatitis on tail and ear. (**E**) Representative images of spleen and PLNs from 26-day-old control or *Foxp3*^Cre^*Pik3c3*^fl/fl^ mice (left) and quantification (right) of the relative (normalized to average control in each experiment) total cell numbers from indicated tissues of 20- to 30-day-old control (*n* =  10 for the spleen and 8 for the PLNs) or *Foxp3*^Cre^*Pik3c3*^fl/fl^ (*n* =  9 for the spleen and 7 for the PLNs) mice. (**F**) Quantification of the frequencies (left) and numbers (right) of CD44^high^CD62L^low^ effector/memory cells among TCRβ^+^CD4^+ ^Foxp3-YFP^−^ (denoted as CD4^+ ^Foxp3^−^) or TCRβ^+^CD8^+^ T cells derived from the spleen of 7- to 11-day-old perinatal control or *Foxp3*^Cre^*Pik3c3*^fl/fl^ mice (*n* =  14 per group). (**G**) Flow cytometry analysis (left) and quantification (right) of the frequencies of IFN-γ^+^ cells among ΤCRβ^+^CD4^+ ^Foxp3^ − ^CD44^high^ conventional Τ cells and ΤCRβ^+^CD8^+ ^CD44^high^ T cells from perinatal control (*n* =  11) or *Foxp3*^Cre^*Pik3c3*^fl/fl^ (*n* =  9) mice. (**H**) Quantification of relative (normalized to average control in each experiment) gMFIs of ICOS (*n* =  13 for control and 12 for *Foxp3*^Cre^*Pik3c3*^fl/fl^ mixed BM chimera), GITR (*n* =  11 for control and 10 for *Foxp3*^Cre^*Pik3c3*^fl/fl^ mixed BM chimera), CD73 (*n* =  11 for control and 10 for *Foxp3*^Cre^*Pik3c3*^fl/fl^ mixed BM chimera), PD-1 (*n* =  12 for control and 10 for *Foxp3*^Cre^*Pik3c3*^fl/fl^ mixed BM chimera), TIGIT (*n* =  18 for control and 16 for *Foxp3*^Cre^*Pik3c3*^fl/fl^ mixed BM chimera), and CTLA-4 (*n* =  16 for control and 14 for *Foxp3*^Cre^*Pik3c3*^fl/fl^ mixed BM chimera) in CD44^low^CD62L^high^ cTregs and CD44^high^CD62L^low^ eTregs among total TCRβ^+^CD4^+ ^Foxp3-YFP^ +^ (ICOS, GITR, CD73, PD-1) or TCRβ^+^CD4^+ ^Foxp3^ +^ (TIGIT, CTLA-4) Tregs (all pre-gated on CD45.2^ +^ cells) derived from the spleen of control or *Foxp3*^Cre^*Pik3c3*^fl/fl^ mixed BM chimera mice, as determined by flow cytometry analysis. gMFI, geometric mean fluorescence intensity. (**I**) Quantification of the relative (normalized to average control in each experiment) frequencies of FVD^ +^ non-viable CD44^low^CD62L^high^ cTregs or CD44^high^CD62L^low^ eTregs among total CD4^+ ^Foxp3^ +^ Tregs (pre-gated on CD45.2^ +^ cells) derived from the spleen of 7- to 11-day-old perinatal control or *Foxp3*^Cre^*Pik3c3*^fl/fl^ mice (*n* =  5 per group). FVD, fixable viability dye. (**J**) Quantification of the relative frequencies of FVD^+^ non-viable CD44^low^CD62L^high^ cTregs or CD44^high^CD62L^low^ eTregs among total TCRβ^+^CD4^+ ^Foxp3^+^ Tregs derived from the spleen of control (*n* =  10 at 16 dpi, 11 at 30 dpi) or *Foxp3*^Cre-ERT2^*Pik3c3*^fl/fl^ (*n* =  10 at 16 dpi, 12 at 30 dpi) mice at 16 or 30 dpi. Relative (normalized to average control in each experiment) fold-change comparisons between genotypes in each cell type are shown in bold (**E**, **I**). Data are shown as mean ±  s.e.m. (**A**–**C**, **E**–**J**). Two-tailed Student *t* test (**B**, **C**, **E**–**J)**; NS, not significant. Data are compiled from 1 (**A**), ≥3 (**B**, **H**), 6 (**C**), 8 (**E**, **G**), 11 (**F**), 4 (**I**), or 5 (**J**) independent experiments. Numbers in plots indicate percentages of cells in gates (**B**, **C**, **G**). The numerical data underlying the graphs shown in this figure are found in S9 Data (**A**–**C, E**–**J**).
(TIFF)

S3 Fig Analysis of Tregs from perinatal mice and mixed BM chimeras for transitional and terminal eTreg states. (**A**, **B**) TCRβ^+^CD4^+ ^Foxp3-YFP^ +^ Tregs were sort-purified from the spleen of 10- to 12-day-old perinatal control or *Foxp3*^Cre^*Pik3c3*^fl/fl^ mice and profiled by single-cell RNA-sequencing or single-nuclear RNA-seq (see Materials and methods for details). scRNA-seq and snRNA-seq datasets of perinatal control (*n* =  2) or *Foxp3*^Cre^*Pik3c3*^fl/fl^ (*n* =  3) Tregs were merged before performing subsequent analyses (see Materials and methods for details). UMAP plots depicting the gene expression profiles of cTreg-related genes (*Sell*, *Tcf7*, *Il2ra*) and eTreg-related genes (*Mki67*, *Cd44*, *Il1rl1*, *Klrg1*). Exp., expression; UMAP, Uniform manifold approximation and projection (**A**). Violin plots of the expression level of cTreg- and eTreg-associated genes shown in **A** across each of the three cell states depicted in Fig 3A (**B**). The change in expression (based on log_2_FC) between each comparison is indicated and statistically significant (*P* <  0.05) unless indicated (NS). (**C**) Quantification of the frequencies and numbers of KLRG1^ −^ and KLRG1^ +^ (upper) or ST2^ −^ and ST2^ +^ (lower) populations among TCRβ^+^CD4^+ ^Foxp3-YFP^+^CD44^high^CD62L^low^ eTregs derived from the liver of 7- to 11-day-old perinatal control or *Foxp3*^Cre^*Pik3c3*^fl/fl^ mice (*n* =  16 per group). (**D**) Quantification of the numbers of CD44^low^CD62L^high^ cTregs and CD44^low^CD62L^high^ eTregs among total TCRβ^+^CD4^+ ^Foxp3-YFP^ +^ Tregs derived from the lung (left) or liver (right) of 7 - to 11-day-old perinatal control or *Foxp3*^Cre^*Pik3c3*^fl/fl^ mice (*n* =  16 per group). (**E**) Quantification of the frequencies and numbers of KLRG1^ −^ and KLRG1^ +^ populations among TCRβ^+^CD4^+ ^Foxp3-YFP^+^CD44^high^CD62L^low^ eTregs derived from the spleen (left) or lung (right) of control (*n* =  12 for spleen, 11 for lung) or *Foxp3*^Cre^*Pik3c3*^fl/fl^ (*n* =  10 for spleen, 9 for lung) mixed BM chimera mice. (**F**) Quantification of the frequencies and numbers of ST2^ −^ and ST2^ +^ populations among TCRβ^+^CD4^+ ^Foxp3-YFP^+^CD44^high^CD62L^low^ eTregs derived from the spleen (left) or lung (right) of control (*n* =  12 for spleen, 11 for lung) or *Foxp3*^Cre^*Pik3c3*^fl/fl^ (*n* =  10 for spleen, 9 for lung) mixed BM chimera mice. Data are shown as mean ±  s.e.m. (**C**–**F**). Wilcoxon rank-sum test (**B**) or two-tailed Student *t* test (**C**–**F)**; NS, not significant. Data are compiled from 12 (**C**, **D**) or 3 (**E**, **F**) independent experiments. The numerical data underlying the graphs shown in this figure are found in S10 Data (**C**–**F**). Raw scRNA-seq and snRNA-seq data used for analysis in (**A**, **B**) have been deposited to GEO SuperSeries access number GSE279606.
(TIFF)

S4 Fig Chromatin accessibility analysis of perinatal Vps34-deficient Tregs. (**A**) Quantification of relative (normalized to average control in each experiment) gMFIs of TMRM (*n* =  10 for control, 11 for *Foxp3*^Cre^*Pik3c3*^fl/fl^), MitoTracker Deep Red (*n* =  21 per group), CellROX (*n* =  10 for control, 11 for *Foxp3*^Cre^*Pik3c3*^fl/fl^), and MitoSOX (*n* =  17 per group) in TCRβ^+^CD4^+ ^Foxp3-YFP^+^CD44^low^CD62L^high^ cTregs and TCRβ^+^CD4^+ ^Foxp3-YFP^+^CD44^low^CD62L^high^ eTregs derived from the spleen of control or *Foxp3*^Cre^*Pik3c3*^fl/fl^ mixed BM chimera mice, as determined by flow cytometry analysis. gMFI, geometric mean fluorescence intensity; BM, bone marrow. (**B**) Quantification of relative (normalized to average control in each experiment) gMFIs of CellROX and MitoSOX in TCRβ^+^CD4^+ ^Foxp3-YFP^+^CD44^low^CD62L^high^ cTregs and TCRβ^+^CD4^+ ^Foxp3-YFP^+^CD44^low^CD62L^high^ eTregs derived from the spleen of 7- to 11-day-old perinatal control or *Foxp3*^Cre^*Pik3c3*^fl/fl^ mice, as determined by flow cytometry analysis (*n* =  15 for control and 14 for *Foxp3*^Cre^*Pik3c3*^fl/fl^ for both CellROX and MitoSOX). (**C**) GSEA enrichment plot showing decreased Hallmark TNFα signaling via NFκB signature in Vps34-deficient versus control terminal eTregs from single-cell transcriptome profiling (as described in Fig 3). See also S4 Table. (**D**, **E**) Total TCRβ^+^CD4^+ ^Foxp3-YFP^ +^ Tregs were sort-purified from the spleen and lung of 10-day-old perinatal control (*n* =  4 for spleen, 2 for lung) and *Foxp3*^Cre^*Pik3c3*^fl/fl^ (*n* =  2 for both tissues) mice and profiled by bulk ATAC-seq (see Materials and methods for details). Principal component analysis (PCA) plot shows tissue (i.e., spleen versus lung)- and genotype (i.e., perinatal *Foxp3*^Cre^*Pik3c3*^fl/fl^ versus control)-dependent chromatin alterations, with percentages of variances shown (**D**). Venn diagrams showing the numbers of upregulated (left; log_2_FC >  0.5, FDR <  0.05) and downregulated (right; log_2_FC < –0.5, FDR <  0.05) accessible peaks in perinatal Vps34-deficient versus control Tregs from the spleen (purple) and the lung (orange), including those peaks that are shared between both tissues. See also Fig 4E and S5 Table (**E**). (**F**) scATAC-seq analysis (via multiome profiling) was performed in total TCRβ^+^CD4^+ ^Foxp3-YFP^ +^ Tregs from the spleen of 12-day-old perinatal control (*n* =  1) or *Foxp3*^Cre^*Pik3c3*^fl/fl^ (*n* =  2) mice. Chromatin peak tracks of selective cTreg- (*Ccr7*, *Tcf7*) and eTreg-associated (*Tigit*, *Il10*, *Klrg1*) genes aligned with annotated cTreg (red), transitional eTreg (green), and terminal eTreg (blue) populations as described in Fig 3A. See also S3A and S3B Fig. Exp., expression. (**G**) Differentially accessible chromatin regions (|log_2_FC | >  0.5 and FDR <  0.05) identified in cTregs (left), transitional eTregs (middle) and terminal eTregs (right) via scATAC-seq multiome analysis of perinatal *Foxp3*^Cre^*Pik3c3*^fl/fl^ versus perinatal control mice. See also S6 Table. (**H**) Transcription factor motif enrichment analysis of differentially accessible peaks (|log_2_FC | >  0.5*,* FDR <  0.05) from bulk ATAC-seq data (as described in **D**, **E**) of total TCRβ^+^CD4^+ ^Foxp3-YFP^ +^ Tregs derived from the spleen of perinatal *Foxp3*^Cre^*Pik3c3*^fl/fl^ versus perinatal control mice. AP-1-related transcription factors with predicted decreased activity are labeled. See also S7 Table. (**I**) Transcription factor footprinting analysis from bulk ATAC-seq data (as described in **D**, **E**) of total TCRβ^+^CD4^+ ^Foxp3-YFP^ +^ Tregs derived from the spleen of perinatal *Foxp3*^Cre^*Pik3c3*^fl/fl^ versus perinatal control mice, ranked based on *z*-scores. AP-1-related transcription factors with predicted decreased activity are labeled. See also S8 Table. (**J**) Transcription factor motif enrichment analysis of open chromatin regions with reduced accessibility (log_2_FC <  0) from scATAC-seq data (as described in **F**) of cTregs among total TCRβ^+^CD4^+ ^Foxp3-YFP^ +^ Tregs derived from the spleen of perinatal *Foxp3*^Cre^*Pik3c3*^fl/fl^ versus perinatal control mice. Transcription factors with predicted decreased activity, including AP-1-related transcription factors, are labeled. Relative (normalized to average control in each experiment) fold-change comparisons between genotypes in each cell type are shown in bold (**A**, **B**). Data are shown as mean ±  s.e.m. (**A**, **B**). Two-tailed Student *t* test (**A**, **B**, **I**), Benjamini–Hochberg test (**C**), Wilcoxon rank-sum test (**G**), Fisher’s exact tes*t* (**H**), or binomial test (**J**); NS, not significant. Data are compiled from ≥4 (**A**) or 10 (**B**) independent experiments. The numerical data underlying the graphs shown in this figure are found in S11 Data (**A**, **B**). Raw microarray data used for analysis in (**C**–**J**) have been deposited to GEO SuperSeries access number GSE279606.
(TIFF)

S5 Fig Uvrag in Tregs is dispensable for establishment of immune tolerance and eTreg accumulation. (**A**, **B**) Quantitative PCR for deletion efficiency of *Atg14* (**A**) or *Uvrag* (**B**) in total TCRβ^+^CD4^+ ^Foxp3-YFP^ +^ Tregs that were sort-purified (pooled from spleen and PLNs) from control (*n* =  4) or *Foxp3*^Cre^*Atg14*^fl/fl^ (*n* =  7) mice (**A**) and control or *Foxp3*^Cre^*Uvrag*^fl/fl^ mice (**B**; *n* =  3 per group). PLNs, peripheral lymph nodes. (**C**) Quantification of the relative (normalized to average control in each experiment) total cell numbers from indicated tissues of 4- to 5-month-old control or *Foxp3*^Cre^*Uvrag*^fl/fl^ mice (*n* =  6 per group for both tissues). (**D**) Flow cytometry analysis (upper) and quantification (lower) of frequencies and numbers of CD44^high^CD62L^low^ effector/memory cells among TCRβ^+^CD4^+ ^Foxp3-YFP^−^ (denoted as CD4^+ ^Foxp3^−^) and TCRβ^+^CD8^+^ T cells derived from the spleen of 4- to 5-month-old control or *Foxp3*^Cre^*Uvrag*^fl/fl^ mice (*n* =  6 per group). (**E**) Splenocytes were stimulated with PMA and ionomycin in the presence of GolgiStop for 4 h. Quantification of the frequencies of IFN-γ^+^, IL-4^+ ^, or IL-17A^+^ cells among ΤCRβ^+^CD4^+ ^Foxp3^− ^CD44^high^ conventional Τ cells and IFN-γ^+^ cells among ΤCRβ^+^CD8^+ ^CD44^high^ T cells from 4- to 5-month-old control or *Foxp3*^Cre^*Uvrag*^fl/fl^ mice (*n* =  4 per group). (**F)** Quantification of frequency (left) and number (right) of total ΤCRβ^+^CD4^+ ^Foxp3-YFP^+^ Tregs derived from the spleen of 4- to 5-month-old control or *Foxp3*^Cre^*Uvrag*^fl/fl^ mice (*n* =  6 per group). (**G**) Quantification of frequencies (left) and numbers (right) of TCRβ^+^CD4^+ ^Foxp3-YFP^+^CD44^low^CD62L^high^ cTregs or TCRβ^+^CD4^+ ^Foxp3-YFP^+^CD44^high^CD62L^low^ eTregs derived from the spleen of 4- to 5-month-old control or *Foxp3*^Cre^*Uvrag*^fl/fl^ mice (*n* =  6 per group). (**H**) Flow cytometry analysis (upper) and quantification (lower) of frequencies and numbers of total TCRβ^+^CD4^+ ^Foxp3-YFP^ +^ Tregs derived from the spleen of control mosaic (*n* =  6) or *Foxp3*^Cre/+ ^*Uvrag*^fl/fl^ mosaic (*n* =  5) mice. (**I**) Flow cytometry analysis (upper) and quantification (lower) of frequencies and numbers of TCRβ^+^CD4^+ ^Foxp3-YFP^+^CD44^low^CD62L^high^ cTregs or TCRβ^+^CD4^+ ^Foxp3-YFP^+^CD44^high^CD62L^low^ eTregs derived from the spleen of control mosaic (*n* =  6) or *Foxp3*^Cre/+ ^*Uvrag*^fl/fl^ mosaic (*n* =  5) mice. Data are shown as mean ±  s.e.m. (**A**–**I**). Two-tailed Student *t* test (**A**–**I)**; NS, not significant. Data are compiled from 1 (**A**, **B**), 3 (**C**, **D**, **F**, **G**), or 2 (**E**, **H**, **I**) independent experiments. Numbers in plots indicate percentages of cells in gates (**D**, **H**, **I**). The numerical data underlying the graphs shown in this figure are found in S12 Data (**A**–**I**).
(TIFF)

S6 Fig Atg14 does not contribute to terminal eTreg generation during perinatal life. (**A**) Quantification of frequency (left) and number (right) of total TCRβ^+^CD4^+ ^Foxp3-YFP^ +^ Tregs derived from the spleen of 4- to 5-month-old control (*n* = 10) or *Foxp3*^Cre^*Atg14*^fl/fl^ (*n* = 13) mice. (**B**) Quantification of frequencies (left) and numbers (right) of TCRβ^+^CD4^+ ^Foxp3-YFP^+^CD44^low^CD62L^high^ cTregs or TCRβ^+^CD4^+ ^Foxp3-YFP^+^CD44^high^CD62L^low^ eTregs derived from the spleen of 4- to 5-month-old control (*n* = 10) or *Foxp3*^Cre^*Atg14*^fl/fl^ (*n* = 13) mice. (**C**) Spleen and PLNs of control mosaic or *Foxp3*^Cre/+^*Atg14*^fl/fl^ mosaic mice were pooled, and CD4^+ ^Foxp3-YFP^+^CD44^low^CD62L^high^ cTregs were sort-purified and cultured in vitro in the presence of anti-CD3, anti-CD28, and IL-2 for 72 h. Quantification of the frequency of CD44^high^CD62L^low^ eTreg-like cells (*n* =  7 technical replicates from 2 biological replicates for each group). PLNs, peripheral lymph nodes. (**D**) Quantification of the frequency of Ki67^ +^ cells among total TCRβ^+^CD4^+ ^Foxp3^+^ Tregs, TCRβ^+^CD4^+ ^Foxp3^+ ^CD44^low^CD62L^high^ cTregs, or TCRβ^+^CD4^+ ^Foxp3^+ ^CD44^high^CD62L^low^ eTregs (all pre-gated on CD45.2^+^ cells) derived from the spleen of control or *Foxp3*^Cre^*Atg14*^fl/fl^ mixed BM chimera mice (*n* =  9 per group). BM, bone marrow. (**E**) Quantification of the relative (normalized to average control in each experiment) frequencies of FVD^ +^ non-viable TCRβ^+^CD4^+ ^Foxp3-YFP^+^CD44^low^CD62L^high^ cTregs or TCRβ^+^CD4^+ ^Foxp3-YFP^+^CD44^high^CD62L^low^ eTregs derived from the spleen of control mosaic (*n* =  6) or *Foxp3*^Cre/+^*Atg14*^fl/fl^ mosaic (*n* =  7) mice. FVD, fixable viability dye. (**F**) Quantification of Bim/Mcl1 (left) and Bim/Bcl2 (right) ratios (based on gMFIs) from TCRβ^+^CD4^+ ^Foxp3-YFP^+^CD44^low^CD62L^high^ cTregs or TCRβ^+^CD4^+ ^Foxp3-YFP^+^CD44^high^CD62L^low^ eTregs (all pre-gated on CD45.2^ +^ cells) derived from the spleen of control (*n* =  9 for Mcl1, 5 for Bcl2) or *Foxp3*^Cre^*Atg14*^fl/fl^ (*n* =  10 for Mcl1, 4 for Bcl2) mixed BM chimera mice. gMFI, geometric mean fluorescence intensity. (**G**) CD45.2^+ ^CD4^+ ^Foxp3-YFP^+^CD44^low^CD62L^high^ cTregs and CD45.2^+ ^CD4^+ ^Foxp3-YFP^+^CD44^high^CD62L^low^ eTregs were sort-purified from the spleen of control (*n* =  3) and *Foxp3*^Cre^*Atg14*^fl/fl^ (*n* =  4) mixed BM chimera mice and profiled by bulk ATAC-seq (see Materials and methods for details). Principal component analysis (PCA) plot shows state (i.e., cTregs versus eTregs)- and genotype (i.e., Atg14-deficient versus control)-dependent chromatin alterations, with percentages of variances shown. (**H**) Quantification of the frequencies (left) and numbers (right) of CD44^high^CD62L^low^ effector/memory cells among TCRβ^+^CD4^+ ^Foxp3^−^ and CD8^ +^ (gated as TCRβ^+^CD4^−^) T cells derived from the spleen of 7- to 11-day-old perinatal control or *Foxp3*^Cre^*Atg14*^fl/fl^ mice (*n* =  5 per group). (**I**) Splenocytes were stimulated with PMA and ionomycin in the presence of GolgiStop for 4 h. Quantification of the frequencies of IFN-γ^+^ cells among ΤCRβ^+^CD4^+ ^Foxp3^ − ^CD44^high^ conventional Τ cells and ΤCRβ^+^CD8^+ ^CD44^high^ T cells from 7- to 11-day-old perinatal control or *Foxp3*^Cre^*Atg14*^fl/fl^ mice (*n* =  5 per group). (**J**) Quantification of frequencies (left) and numbers (right) of total ΤCRβ^+^CD4^+ ^Foxp3-YFP^ +^ Tregs, ΤCRβ^+^CD4^+ ^Foxp3-YFP^+^CD44^low^CD62L^high^ cTregs, or CD4^+ ^Foxp3-YFP^+^CD44^high^CD62L^low^ eTregs derived from the spleen of 7- to 11-day-old perinatal control or *Foxp3*^Cre^*Atg14*^fl/fl^ mice (*n* =  5 per group). (**K**) Quantification of frequencies (left) and numbers (right) of total ΤCRβ^+^CD4^+ ^Foxp3-YFP^ +^ Tregs derived from the lung and liver of 7- to 11-day-old perinatal control or *Foxp3*^Cre^*Atg14*^fl/fl^ mice (*n* =  5 per group for both tissues). (**L**) Quantification of the relative (normalized to average control in each experiment) frequencies of FVD^ +^ non-viable cells among ΤCRβ^+^CD4^+ ^Foxp3^+ ^CD44^low^CD62L^high^ cTregs or ΤCRβ^+^CD4^+ ^Foxp3^+ ^CD44^high^CD62L^low^ eTregs derived from the spleen of 7- to 11-day-old perinatal control or *Foxp3*^Cre^*Atg14*^fl/fl^ mice (*n* =  5 per group). Relative (normalized to average control in each experiment) fold-change comparisons between genotypes in each cell type are shown in bold (**E**, **F**, **J**–**L**). Data are shown as mean ±  s.e.m. (**A**–**F**, **H**–**L**). Two-tailed Student *t* test (**A**–**F, H**–**L)**; NS, not significant. Data are compiled from 5 (**A**, **B**), ≥2 (**C**, **F**), 4 (**D**, **H**–**L**), or 3 (**E**) independent experiments. The numerical data underlying the graphs shown in this figure are found in S13 Data (**A**–**F**, **H**–**L**). Raw ATAC-seq data used for analysis in (**G**) have been deposited to GEO SuperSeries access number GSE279606.
(TIFF)

S7 Fig Targeting of Vps34 reduces B16F10 tumor growth and working model for Vps34-dependent regulation of eTreg transitional heterogeneity and maintenance. (**A**−**C**) Control (*n* =  5) or *Foxp3*^Cre-ERT2^*Pik3c3*^fl/fl^ (*n* =  7) mice were inoculated with B16F10 tumor cells and treated with TAM on days 7 to 11 after tumor inoculation (see Fig 7A for experimental schematic). Tumor growth curves (left) and tumor weights (right) at endpoint (day 31) in indicated mice (**A**). Quantification of the frequency of control and Vps34-deficient TCRβ^+^CD4^+ ^GFP^+^YFP^ +^ Tregs derived from the spleen or B16F10 tumor of control or *Foxp3*^Cre-ERT2^*Pik3c3*^fl/fl^ mice at day 31 after tumor inoculation (**B**). Quantification of the ratio of total TCRβ^+^CD8^+^ T cells to total TCRβ^+^CD4^+ ^GFP^ +^ Tregs derived from the spleen or B16F10 tumor of control or *Foxp3*^Cre-ERT2^*Pik3c3*^fl/fl^ mice at day 31 after tumor inoculation (**C**). (**D**) Proposed “two-hit” model by which Vps34 orchestrates eTreg transitional heterogeneity and functional adaptation. Vps34 coordinates both terminal eTreg generation during perinatal life (left) and eTreg maintenance after perinatal life (right) to respectively establish immune and tissue tolerance versus suppress anti-tumor immunity. Vps34 complex I (Atg14) but not Vps34 complex II (Uvrag) selectively orchestrates eTreg maintenance after perinatal life. TFs, transcription factors. Image created using BioRender. Data are shown as mean ±  s.e.m. (**A**−**C**). Two-way ANOVA (tumor volume; **A**), Welch’s *t* test (tumor weight; **A**), or two-tailed Student *t* test (**B**, **C**); NS, not significant. Data are representative of 3 independent experiments (**A**–**C**). The numerical data underlying the graphs shown in this figure are found in S14 Data (**A**–**C**).
(TIFF)

S1 File Gating strategies used in this study. (PDF)

S1 Table Differentially expressed genes in Vps34-deficient Tregs compared to control Tregs. (XLSX)

S2 Table Lists containing the Vps34-suppressed signature genes and Vps34-activated signature genes. (XLSX)

S3 Table Differentially expressed genes in Vps34-deficient versus control perinatal Treg states. (XLSX)

S4 Table Gene set enrichment analysis of Hallmark gene signatures in Vps34-deficient versus WT perinatal Treg states. (XLSX)

S5 Table Differentially accessible chromatin regions in Vps34-deficient versus control perinatal Tregs from lung and spleen. (XLSX)

S6 Table Differentially accessible chromatin regions in Vps34-deficient versus control perinatal Treg states. (XLSX)

S7 Table Transcription factor motif enrichment analysis of Vps34-deficient versus control perinatal Tregs from lung or spleen. (XLSX)

S8 Table Transcription factor footprinting analysis of Vps34-deficient versus control perinatal Tregs from lung or spleen. (XLSX)

S9 Table Transcription factor motif enrichment analysis of Vps34-deficient versus control perinatal Treg states. (XLSX)

S10 Table Differentially expressed genes in Atg14-deficient versus control cTregs or Atg14-deficient versus control eTregs. (XLSX)

S11 Table Gene set enrichment analysis of Atg14-deficient versus control cTregs or Atg14-deficient versus control eTregs. (XLSX)

S12 Table Chromatin accessibility analysis in Atg14-deficient versus control cTregs or Atg14-deficient versus control eTregs. (XLSX)

S13 Table Transcription factor footprinting analysis in Atg14-deficient versus control eTregs. (XLSX)

S14 Table List of genes in Vps34-activated eTreg signature or Atg14-activated eTreg signature. (XLSX)

S15 Table Flow cytometry reagents and dilutions used in this study. (XLSX)

S1 Data Source data for Fig 1 numerical data. (XLSX)

S2 Data Source data for Fig 2 numerical data. (XLSX)

S3 Data Source data for Fig 3 numerical data. (XLSX)

S4 Data Source data for Fig 4 numerical data. (XLSX)

S5 Data Source data for Fig 5 numerical data. (XLSX)

S6 Data Source data for Fig 6 numerical data. (XLSX)

S7 Data Source data for Fig 7 numerical data. (XLSX)

S8 Data Source data for S1 Fig numerical data. (XLSX)

S9 Data Source data for S2 Fig numerical data. (XLSX)

S10 Data Source data for S3 Fig numerical data. (XLSX)

S11 Data Source data for S4 Fig numerical data. (XLSX)

S12 Data Source data for S5 Fig numerical data. (XLSX)

S13 Data Source data for S6 Fig numerical data. (XLSX)

S14 Data Source data for S7 Fig numerical data. (XLSX)

## Abbreviations

ATAC,: assay for transposase-accessible chromatin;
BM: bone marrow
BSA: bovine serum albumin
CPM: counts per million
cTreg: central Treg
eTreg: effector Treg
FBS: fetal bovine serum
FC: fold change
FDR: false discovery rate
FVD: fixable viability dye
GSEA: gene set enrichment analysis
H&E: hematoxylin and eosin
HNSCC: head and neck squamous cell carcinoma
IACUC: Institutional Animal Care and Use Committee
LN: lymph node
LP: lamina propria
PCA: principal component analysis
PI3Ks: phosphatidylinositol-3-kinases
PLNs: peripheral lymph nodes
PMA: phorbol myristate acetate
PPs: Peyer’s patches
PSEA: peak set enrichment analysis
s.c.: subcutaneously
SCLC: small cell lung cancer
TILs: tumor-infiltrating lymphocytes
TME: tumor microenvironment
Treg: regulatory T cell
UMAP: Uniform Manifold Approximation and Projection
