# Supplementary figures and images for: Vps34-orchestrated lipid signaling processes regulate the transitional heterogeneity and functional adaptation of effector regulatory T cells
Source: PLoS Biol. 2025 Apr 11;23(4):e3003074. doi: 10.1371/journal.pbio.3003074 (PMC11990774; doi:10.1371/journal.pbio.3003074)

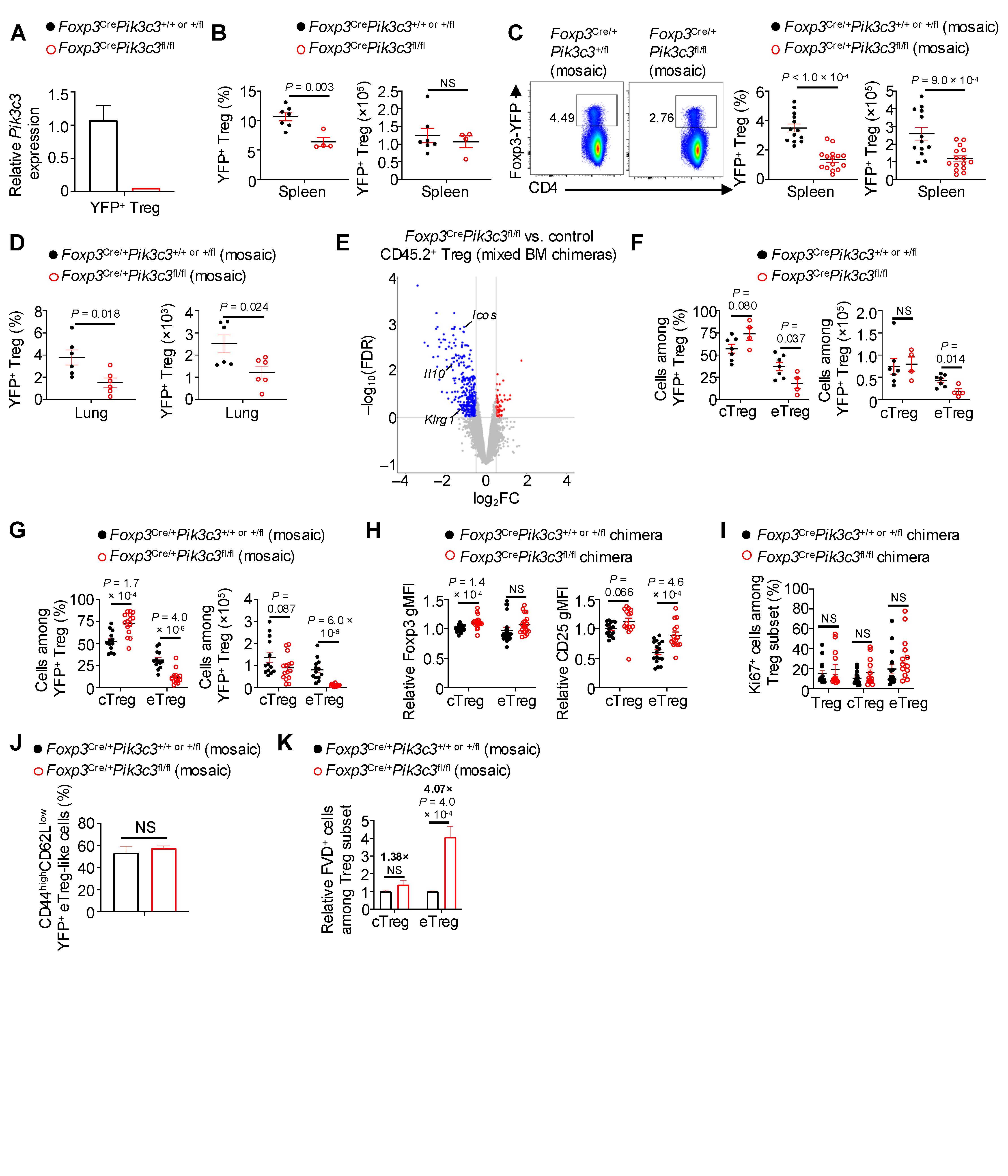

Supplement: S1 Fig — (A) Quantitative PCR analysis for Pik3c3 deletion efficiency in TCRβ+CD4+ Foxp3-YFP + Tregs that were sort-purified (pooled from spleen and PLNs) from 17- to 42-day-old control (n = 4) or Foxp3CrePik3c3fl/fl (n = 2) mice. PLNs, peripheral lymph nodes. (B) Quantification of frequency (left) and number (right) of total TCRβ+CD4+ Foxp3-YFP+ Tregs derived from the spleen of 20- to 30-day-old control (n = 7) or Foxp3CrePik3c3fl/fl (n = 4) mice. (C) Flow cytometry analysis (left) and quantification (right) of frequencies and numbers of total TCRβ+CD4+ Foxp3-YFP + Tregs derived from the spleen of control mosaic (n = 13) or Foxp3Cre/+ Pik3c3fl/fl mosaic (n = 15) mice. (D) Quantification of the frequency (left) and number (right) of total TCRβ+CD4+ Foxp3-YFP + Tregs derived from the lung of control mosaic or Foxp3Cre/+ Pik3c3fl/fl mosaic mice (n = 6 per group). (E) CD4+ Foxp3-YFP + Tregs (CD45.2+) were sort-purified from the spleen of control (n = 4) or Foxp3CrePik3c3fl/fl (n = 5) mixed BM chimera mice and profiled by microarray analysis (see Materials and methods for details). Volcano plot depicting upregulated (red) and downregulated (blue) genes, with select eTreg-associated genes labeled. See also S1 Table. BM, bone marrow. (F) Quantification of frequencies (left) and numbers (right) of CD44lowCD62Lhigh cTregs or CD44highCD62Llow eTregs among total TCRβ+CD4+ Foxp3-YFP + Tregs derived from the spleen of 20- to 30-day-old control (n = 7) or Foxp3CrePik3c3fl/fl (n = 4) mice. (G) Quantification of frequencies (left) and numbers (right) of CD44lowCD62Lhigh cTregs or CD44highCD62Llow eTregs among total TCRβ+CD4+ Foxp3-YFP + Tregs derived from the spleen of control mosaic (n = 13) or Foxp3Cre/+ Pik3c3fl/fl mosaic (n = 15) mice. (H) Quantification of relative (normalized to average control in each experiment) gMFIs of Foxp3 and CD25 in CD44lowCD62Lhigh cTregs and CD44highCD62Llow eTregs among total TCRβ+CD4+ Foxp3+ (Foxp3) or TCRβ+CD4+ Foxp3-YFP+ (CD25) Tregs (all pre-gated on C [file pbio.3003074.s001.tiff]

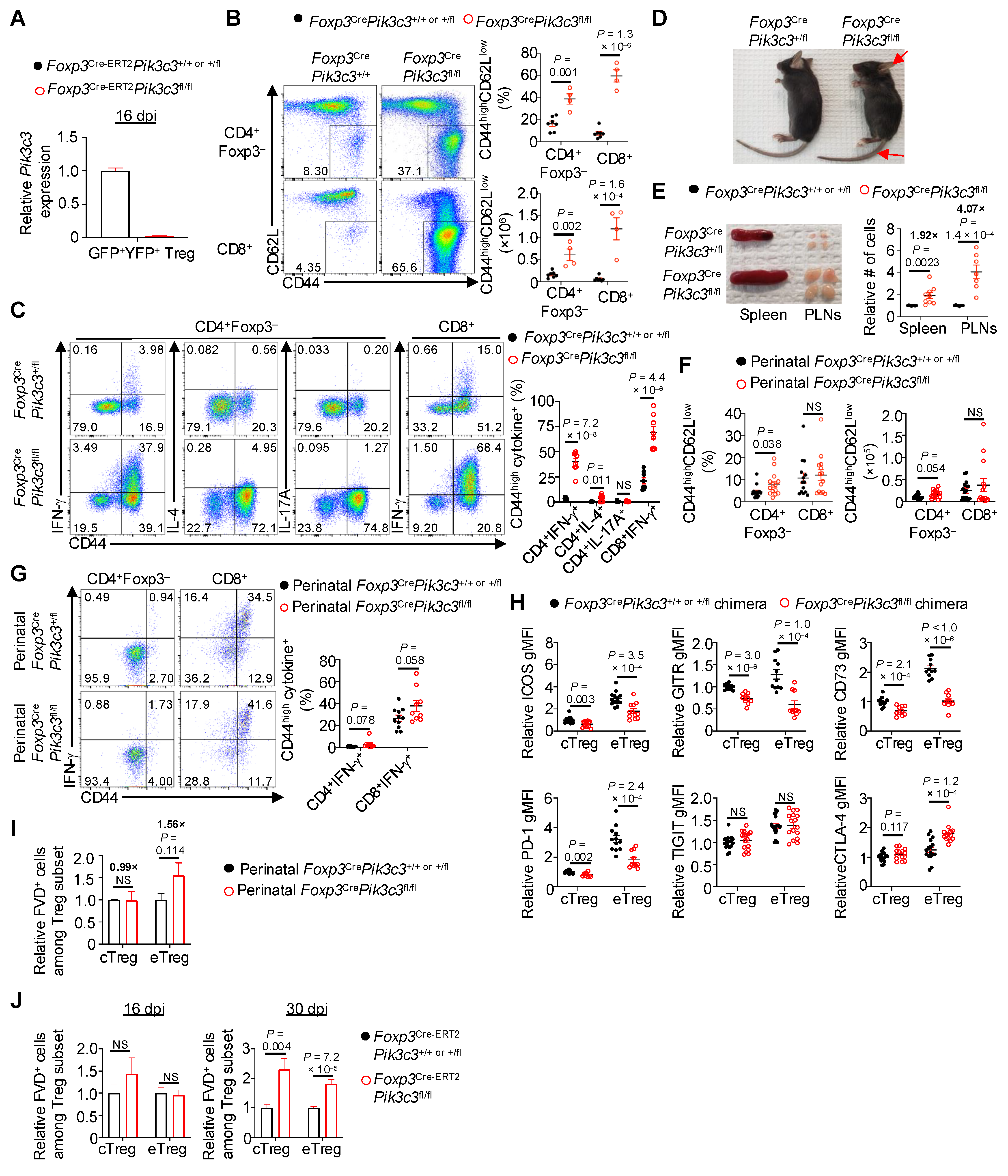

Supplement: S2 Fig — (A) Quantitative PCR analysis for deletion efficiency of Pik3c3 in TCRβ+CD4+ GFP+YFP+ Tregs that were sort-purified (pooled from spleen and PLNs) from control or Foxp3CrePik3c3fl/fl (n = 2 per group) mice 16 days after beginning tamoxifen treatment (16 dpi). PLNs, peripheral lymph nodes; dpi, days post-injection. (B) Flow cytometry analysis (left) and quantification (right) of frequencies and numbers of CD44highCD62Llow effector/memory cells among TCRβ+CD4+ Foxp3-YFP− (denoted as CD4+ Foxp3−) and TCRβ+CD8+ T cells derived from the spleen of 16- to 23-day-old control (n = 7) or Foxp3CrePik3c3fl/fl (n = 4) mice. (C) Splenocytes were stimulated with PMA and ionomycin in the presence of GolgiStop for 4 h. Flow cytometry analysis (left) and quantification (right) of the frequencies of IFN-γ+, IL-4+, or IL-17A+ cells among ΤCRβ+CD4+ Foxp3 − CD44high conventional Τ cells and IFN-γ+ cells among ΤCRβ+CD8+ CD44high T cells from 20- to 30-day-old control or Foxp3CrePik3c3fl/fl mice (n = 8 per group). (D) Representative images of 26-day-old control and Foxp3CrePik3c3fl/fl mice. Arrows indicate sites of focal dermatitis on tail and ear. (E) Representative images of spleen and PLNs from 26-day-old control or Foxp3CrePik3c3fl/fl mice (left) and quantification (right) of the relative (normalized to average control in each experiment) total cell numbers from indicated tissues of 20- to 30-day-old control (n = 10 for the spleen and 8 for the PLNs) or Foxp3CrePik3c3fl/fl (n = 9 for the spleen and 7 for the PLNs) mice. (F) Quantification of the frequencies (left) and numbers (right) of CD44highCD62Llow effector/memory cells among TCRβ+CD4+ Foxp3-YFP− (denoted as CD4+ Foxp3−) or TCRβ+CD8+ T cells derived from the spleen of 7- to 11-day-old perinatal control or Foxp3CrePik3c3fl/fl mice (n = 14 per group). (G) Flow cytometry analysis (left) and quantification (right) of the frequencies of IFN-γ+ cells among ΤCRβ+CD4+ Foxp3 − CD44high conventional Τ cells and ΤCRβ+CD8+ CD44high T cells fro [file pbio.3003074.s002.tiff]

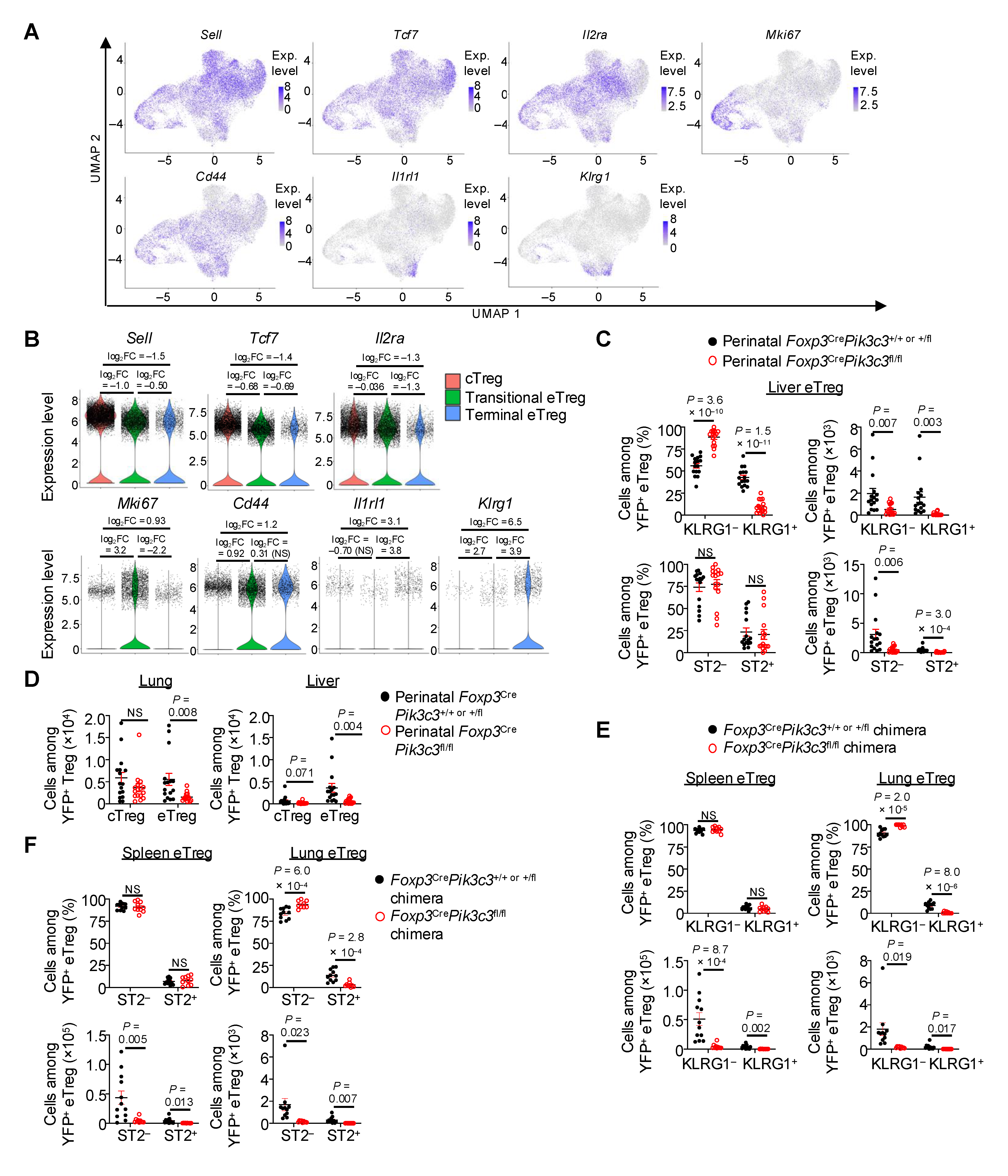

Supplement: S3 Fig — (A, B) TCRβ+CD4+ Foxp3-YFP + Tregs were sort-purified from the spleen of 10- to 12-day-old perinatal control or Foxp3CrePik3c3fl/fl mice and profiled by single-cell RNA-sequencing or single-nuclear RNA-seq (see Materials and methods for details). scRNA-seq and snRNA-seq datasets of perinatal control (n = 2) or Foxp3CrePik3c3fl/fl (n = 3) Tregs were merged before performing subsequent analyses (see Materials and methods for details). UMAP plots depicting the gene expression profiles of cTreg-related genes (Sell, Tcf7, Il2ra) and eTreg-related genes (Mki67, Cd44, Il1rl1, Klrg1). Exp., expression; UMAP, Uniform manifold approximation and projection (A). Violin plots of the expression level of cTreg- and eTreg-associated genes shown in A across each of the three cell states depicted in Fig 3A (B). The change in expression (based on log2FC) between each comparison is indicated and statistically significant (P < 0.05) unless indicated (NS). (C) Quantification of the frequencies and numbers of KLRG1 − and KLRG1 + (upper) or ST2 − and ST2 + (lower) populations among TCRβ+CD4+ Foxp3-YFP+CD44highCD62Llow eTregs derived from the liver of 7- to 11-day-old perinatal control or Foxp3CrePik3c3fl/fl mice (n = 16 per group). (D) Quantification of the numbers of CD44lowCD62Lhigh cTregs and CD44lowCD62Lhigh eTregs among total TCRβ+CD4+ Foxp3-YFP + Tregs derived from the lung (left) or liver (right) of 7 - to 11-day-old perinatal control or Foxp3CrePik3c3fl/fl mice (n = 16 per group). (E) Quantification of the frequencies and numbers of KLRG1 − and KLRG1 + populations among TCRβ+CD4+ Foxp3-YFP+CD44highCD62Llow eTregs derived from the spleen (left) or lung (right) of control (n = 12 for spleen, 11 for lung) or Foxp3CrePik3c3fl/fl (n = 10 for spleen, 9 for lung) mixed BM chimera mice. (F) Quantification of the frequencies and numbers of ST2 − and ST2 + populations among TCRβ+CD4+ Foxp3-YFP+CD44highCD62Llow eTregs derived from the spleen (left) or lung (right) of control (n = 12 for splee [file pbio.3003074.s003.tiff]

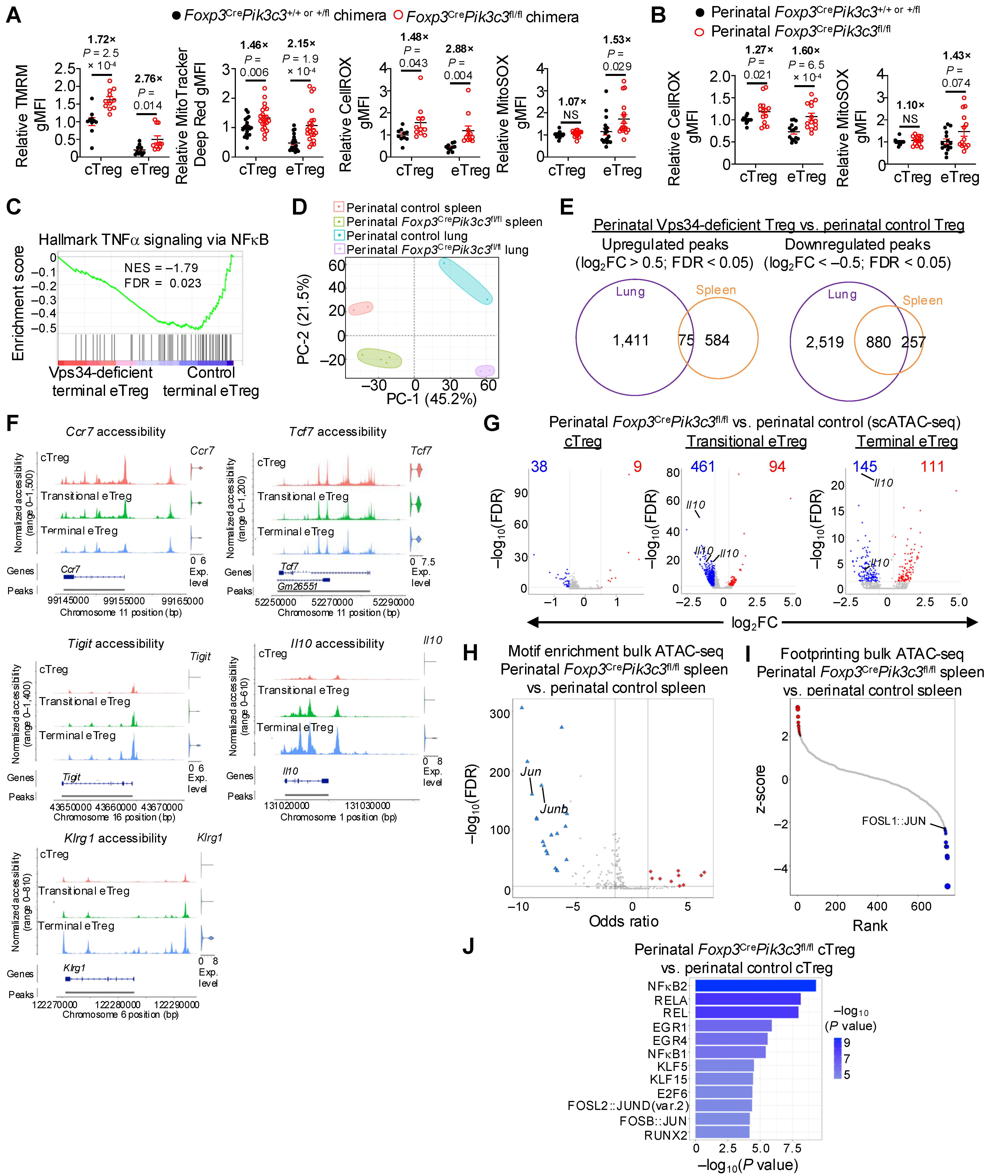

Supplement: S4 Fig — (A) Quantification of relative (normalized to average control in each experiment) gMFIs of TMRM (n = 10 for control, 11 for Foxp3CrePik3c3fl/fl), MitoTracker Deep Red (n = 21 per group), CellROX (n = 10 for control, 11 for Foxp3CrePik3c3fl/fl), and MitoSOX (n = 17 per group) in TCRβ+CD4+ Foxp3-YFP+CD44lowCD62Lhigh cTregs and TCRβ+CD4+ Foxp3-YFP+CD44lowCD62Lhigh eTregs derived from the spleen of control or Foxp3CrePik3c3fl/fl mixed BM chimera mice, as determined by flow cytometry analysis. gMFI, geometric mean fluorescence intensity; BM, bone marrow. (B) Quantification of relative (normalized to average control in each experiment) gMFIs of CellROX and MitoSOX in TCRβ+CD4+ Foxp3-YFP+CD44lowCD62Lhigh cTregs and TCRβ+CD4+ Foxp3-YFP+CD44lowCD62Lhigh eTregs derived from the spleen of 7- to 11-day-old perinatal control or Foxp3CrePik3c3fl/fl mice, as determined by flow cytometry analysis (n = 15 for control and 14 for Foxp3CrePik3c3fl/fl for both CellROX and MitoSOX). (C) GSEA enrichment plot showing decreased Hallmark TNFα signaling via NFκB signature in Vps34-deficient versus control terminal eTregs from single-cell transcriptome profiling (as described in Fig 3). See also S4 Table. (D, E) Total TCRβ+CD4+ Foxp3-YFP + Tregs were sort-purified from the spleen and lung of 10-day-old perinatal control (n = 4 for spleen, 2 for lung) and Foxp3CrePik3c3fl/fl (n = 2 for both tissues) mice and profiled by bulk ATAC-seq (see Materials and methods for details). Principal component analysis (PCA) plot shows tissue (i.e., spleen versus lung)- and genotype (i.e., perinatal Foxp3CrePik3c3fl/fl versus control)-dependent chromatin alterations, with percentages of variances shown (D). Venn diagrams showing the numbers of upregulated (left; log2FC > 0.5, FDR < 0.05) and downregulated (right; log2FC < –0.5, FDR < 0.05) accessible peaks in perinatal Vps34-deficient versus control Tregs from the spleen (purple) and the lung (orange), including those peaks that are shared between both tissues. [file pbio.3003074.s004.tiff]

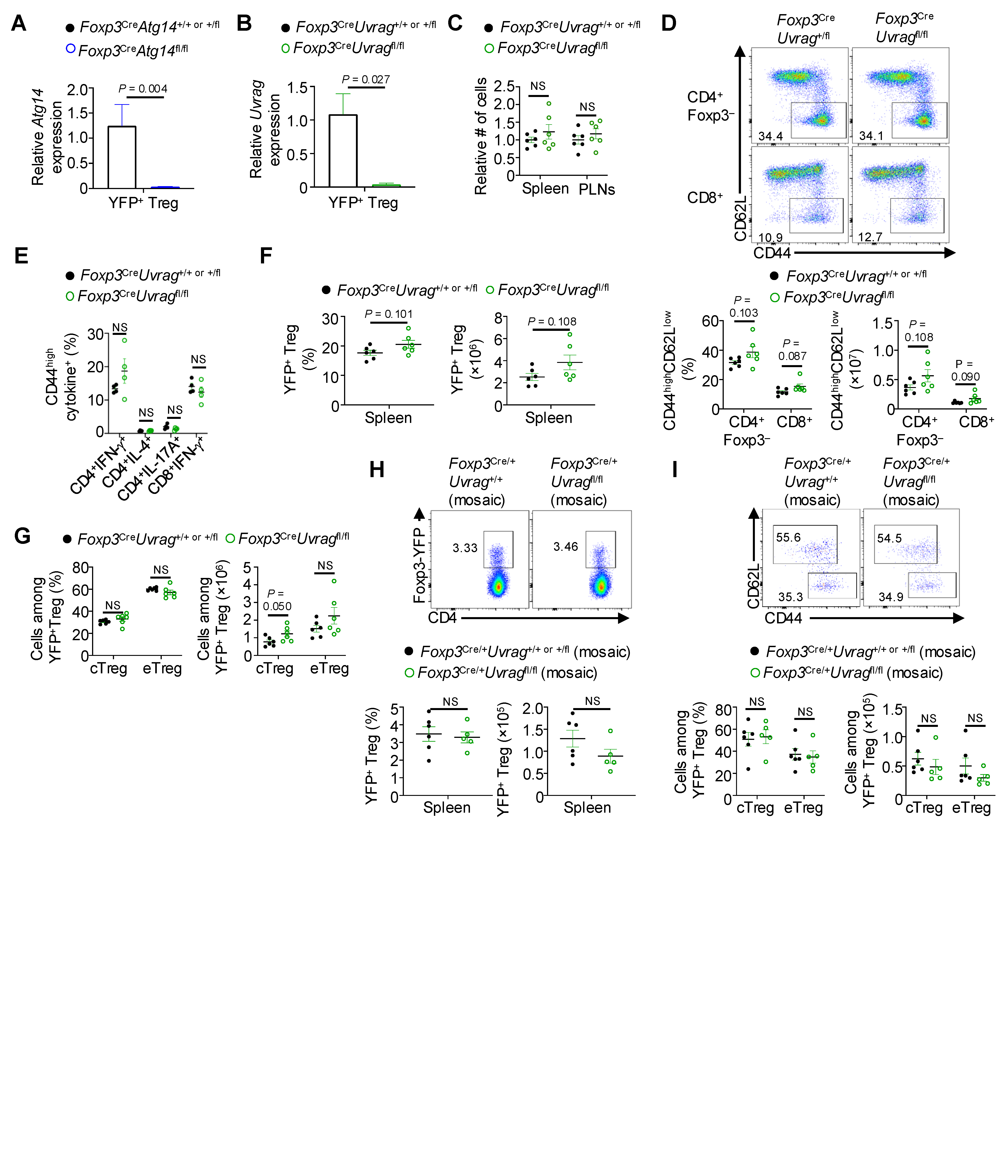

Supplement: S5 Fig — (A, B) Quantitative PCR for deletion efficiency of Atg14 (A) or Uvrag (B) in total TCRβ+CD4+ Foxp3-YFP + Tregs that were sort-purified (pooled from spleen and PLNs) from control (n = 4) or Foxp3CreAtg14fl/fl (n = 7) mice (A) and control or Foxp3CreUvragfl/fl mice (B; n = 3 per group). PLNs, peripheral lymph nodes. (C) Quantification of the relative (normalized to average control in each experiment) total cell numbers from indicated tissues of 4- to 5-month-old control or Foxp3CreUvragfl/fl mice (n = 6 per group for both tissues). (D) Flow cytometry analysis (upper) and quantification (lower) of frequencies and numbers of CD44highCD62Llow effector/memory cells among TCRβ+CD4+ Foxp3-YFP− (denoted as CD4+ Foxp3−) and TCRβ+CD8+ T cells derived from the spleen of 4- to 5-month-old control or Foxp3CreUvragfl/fl mice (n = 6 per group). (E) Splenocytes were stimulated with PMA and ionomycin in the presence of GolgiStop for 4 h. Quantification of the frequencies of IFN-γ+, IL-4+ , or IL-17A+ cells among ΤCRβ+CD4+ Foxp3− CD44high conventional Τ cells and IFN-γ+ cells among ΤCRβ+CD8+ CD44high T cells from 4- to 5-month-old control or Foxp3CreUvragfl/fl mice (n = 4 per group). (F) Quantification of frequency (left) and number (right) of total ΤCRβ+CD4+ Foxp3-YFP+ Tregs derived from the spleen of 4- to 5-month-old control or Foxp3CreUvragfl/fl mice (n = 6 per group). (G) Quantification of frequencies (left) and numbers (right) of TCRβ+CD4+ Foxp3-YFP+CD44lowCD62Lhigh cTregs or TCRβ+CD4+ Foxp3-YFP+CD44highCD62Llow eTregs derived from the spleen of 4- to 5-month-old control or Foxp3CreUvragfl/fl mice (n = 6 per group). (H) Flow cytometry analysis (upper) and quantification (lower) of frequencies and numbers of total TCRβ+CD4+ Foxp3-YFP + Tregs derived from the spleen of control mosaic (n = 6) or Foxp3Cre/+ Uvragfl/fl mosaic (n = 5) mice. (I) Flow cytometry analysis (upper) and quantification (lower) of frequencies and numbers of TCRβ+CD4+ Foxp3-YFP+CD44lowCD62Lhigh cTregs or TCRβ+C [file pbio.3003074.s005.tiff]

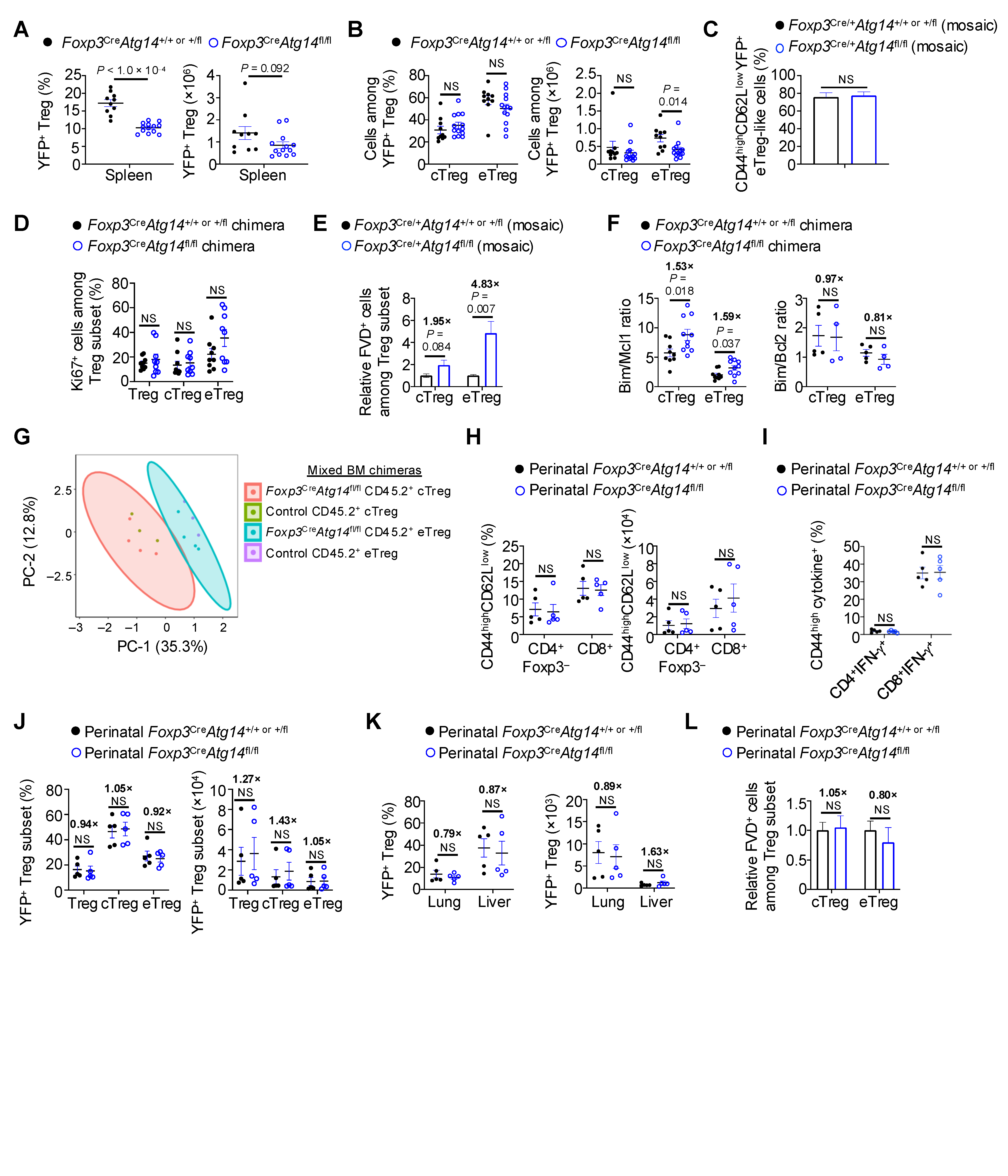

Supplement: S6 Fig — (A) Quantification of frequency (left) and number (right) of total TCRβ+CD4+ Foxp3-YFP + Tregs derived from the spleen of 4- to 5-month-old control (n = 10) or Foxp3CreAtg14fl/fl (n = 13) mice. (B) Quantification of frequencies (left) and numbers (right) of TCRβ+CD4+ Foxp3-YFP+CD44lowCD62Lhigh cTregs or TCRβ+CD4+ Foxp3-YFP+CD44highCD62Llow eTregs derived from the spleen of 4- to 5-month-old control (n = 10) or Foxp3CreAtg14fl/fl (n = 13) mice. (C) Spleen and PLNs of control mosaic or Foxp3Cre/+Atg14fl/fl mosaic mice were pooled, and CD4+ Foxp3-YFP+CD44lowCD62Lhigh cTregs were sort-purified and cultured in vitro in the presence of anti-CD3, anti-CD28, and IL-2 for 72 h. Quantification of the frequency of CD44highCD62Llow eTreg-like cells (n = 7 technical replicates from 2 biological replicates for each group). PLNs, peripheral lymph nodes. (D) Quantification of the frequency of Ki67 + cells among total TCRβ+CD4+ Foxp3+ Tregs, TCRβ+CD4+ Foxp3+ CD44lowCD62Lhigh cTregs, or TCRβ+CD4+ Foxp3+ CD44highCD62Llow eTregs (all pre-gated on CD45.2+ cells) derived from the spleen of control or Foxp3CreAtg14fl/fl mixed BM chimera mice (n = 9 per group). BM, bone marrow. (E) Quantification of the relative (normalized to average control in each experiment) frequencies of FVD + non-viable TCRβ+CD4+ Foxp3-YFP+CD44lowCD62Lhigh cTregs or TCRβ+CD4+ Foxp3-YFP+CD44highCD62Llow eTregs derived from the spleen of control mosaic (n = 6) or Foxp3Cre/+Atg14fl/fl mosaic (n = 7) mice. FVD, fixable viability dye. (F) Quantification of Bim/Mcl1 (left) and Bim/Bcl2 (right) ratios (based on gMFIs) from TCRβ+CD4+ Foxp3-YFP+CD44lowCD62Lhigh cTregs or TCRβ+CD4+ Foxp3-YFP+CD44highCD62Llow eTregs (all pre-gated on CD45.2 + cells) derived from the spleen of control (n = 9 for Mcl1, 5 for Bcl2) or Foxp3CreAtg14fl/fl (n = 10 for Mcl1, 4 for Bcl2) mixed BM chimera mice. gMFI, geometric mean fluorescence intensity. (G) CD45.2+ CD4+ Foxp3-YFP+CD44lowCD62Lhigh cTregs and CD45.2+ CD4+ Foxp3-YFP+CD44highCD62Llow eTr [file pbio.3003074.s006.tiff]

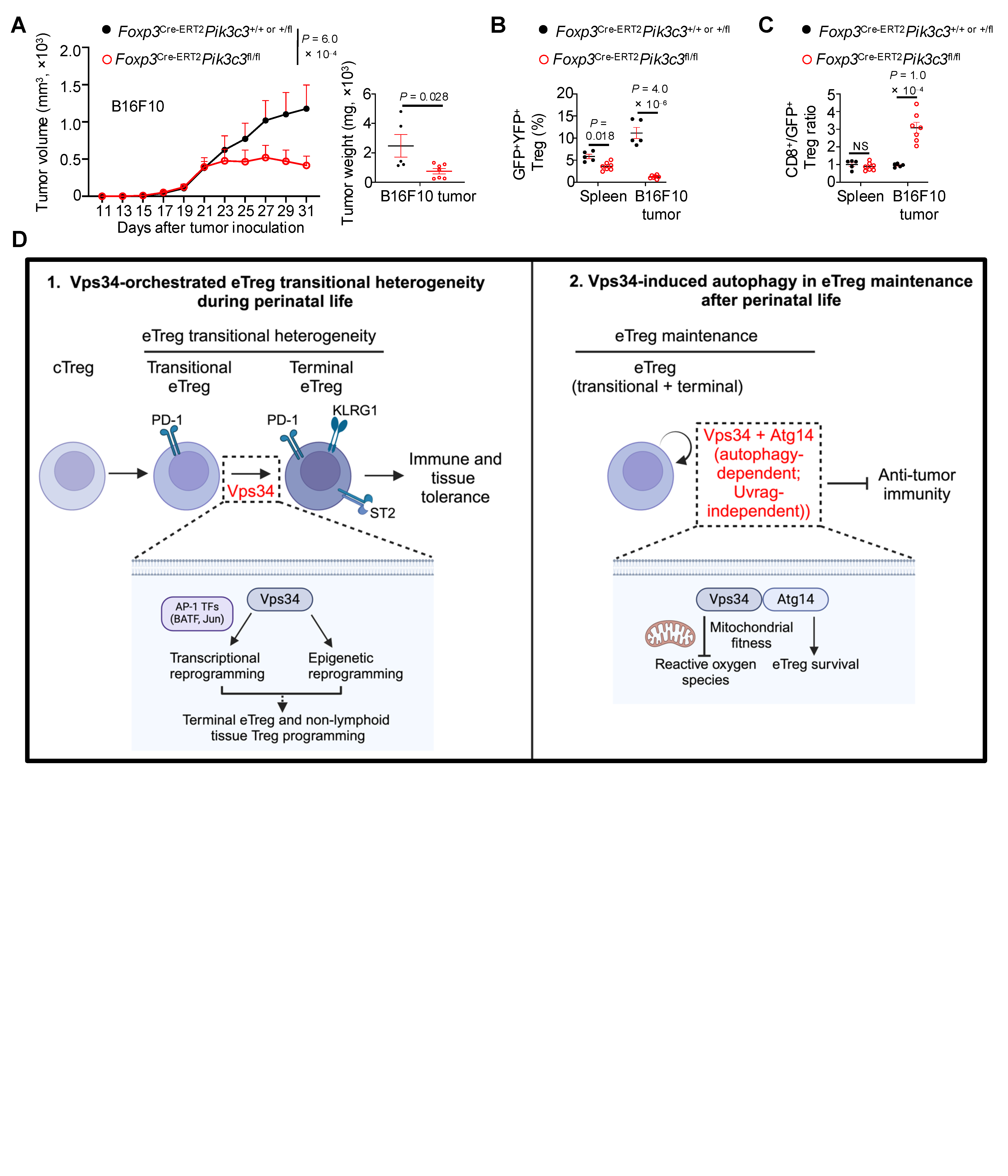

Supplement: S7 Fig — (A−C) Control (n = 5) or Foxp3Cre-ERT2Pik3c3fl/fl (n = 7) mice were inoculated with B16F10 tumor cells and treated with TAM on days 7 to 11 after tumor inoculation (see Fig 7A for experimental schematic). Tumor growth curves (left) and tumor weights (right) at endpoint (day 31) in indicated mice (A). Quantification of the frequency of control and Vps34-deficient TCRβ+CD4+ GFP+YFP + Tregs derived from the spleen or B16F10 tumor of control or Foxp3Cre-ERT2Pik3c3fl/fl mice at day 31 after tumor inoculation (B). Quantification of the ratio of total TCRβ+CD8+ T cells to total TCRβ+CD4+ GFP + Tregs derived from the spleen or B16F10 tumor of control or Foxp3Cre-ERT2Pik3c3fl/fl mice at day 31 after tumor inoculation (C). (D) Proposed “two-hit” model by which Vps34 orchestrates eTreg transitional heterogeneity and functional adaptation. Vps34 coordinates both terminal eTreg generation during perinatal life (left) and eTreg maintenance after perinatal life (right) to respectively establish immune and tissue tolerance versus suppress anti-tumor immunity. Vps34 complex I (Atg14) but not Vps34 complex II (Uvrag) selectively orchestrates eTreg maintenance after perinatal life. TFs, transcription factors. Image created using BioRender. Data are shown as mean ± s.e.m. (A−C). Two-way ANOVA (tumor volume; A), Welch’s t test (tumor weight; A), or two-tailed Student t test (B, C); NS, not significant. Data are representative of 3 independent experiments (A–C). The numerical data underlying the graphs shown in this figure are found in S14 Data (A–C). (TIFF) [file pbio.3003074.s007.tiff]
