# Supplementary material for: Vps34-orchestrated lipid signaling processes regulate the transitional heterogeneity and functional adaptation of effector regulatory T cells
Source: PLoS Biol. 2025 Apr 11;23(4):e3003074. doi: 10.1371/journal.pbio.3003074 (PMC11990774; doi:10.1371/journal.pbio.3003074)

A. Gating strategy for Figs 1A, 1F, and 2H (ICOS, GITR, CD73, PD-1):

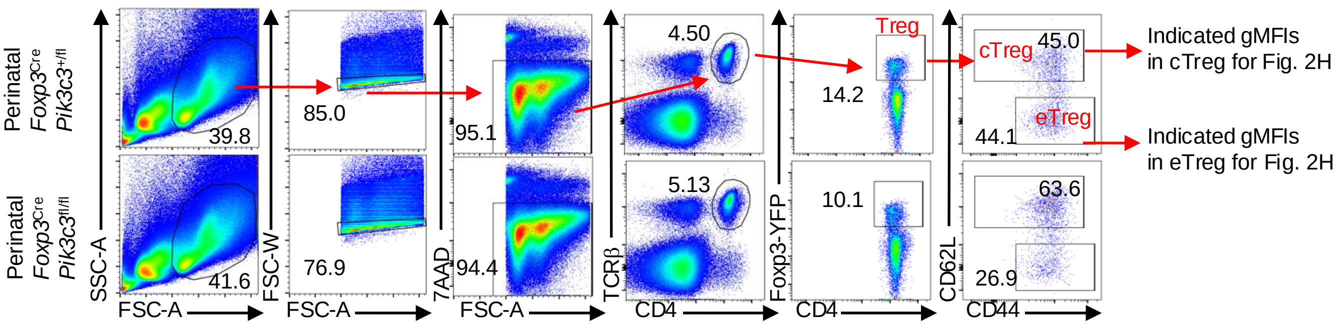

B. Gating strategy for Figs 1B and 3J (lung)

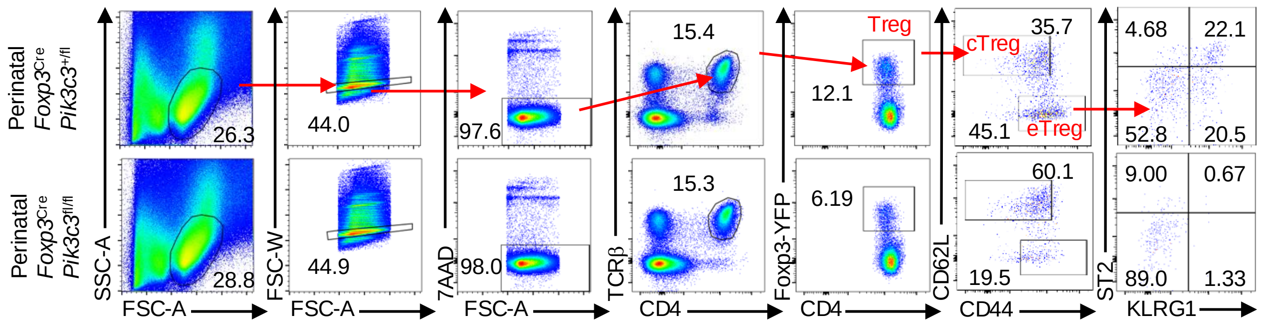

C. Gating strategy for Fig 1B (liver)

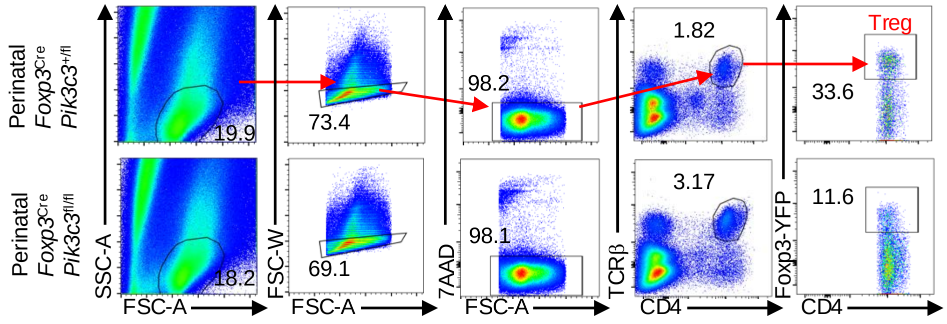

D. Gating strategy for Figs. 1C, 1G, S1H (CD25) and S2H (ICOS, GITR, CD73, PD-1):

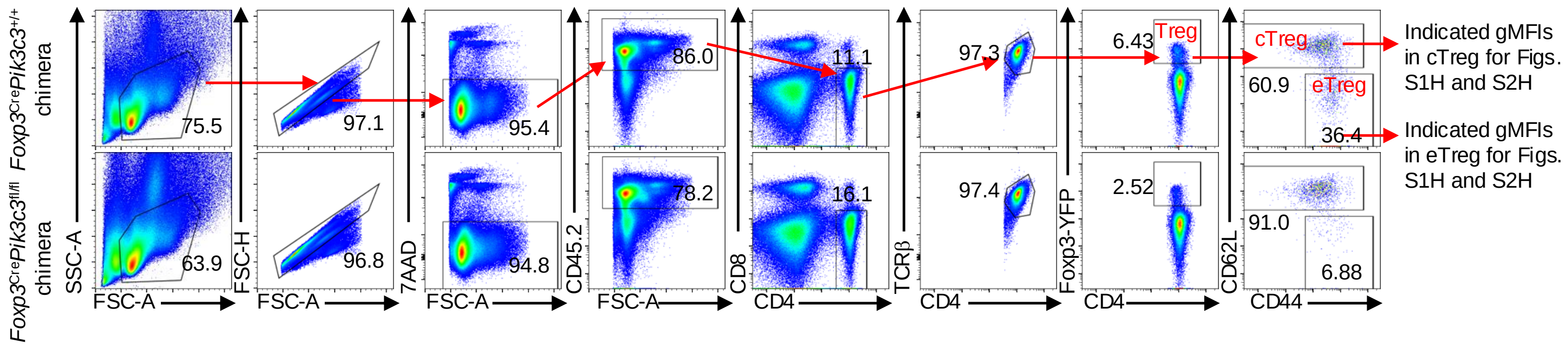

E. Gating strategy for Fig 1H

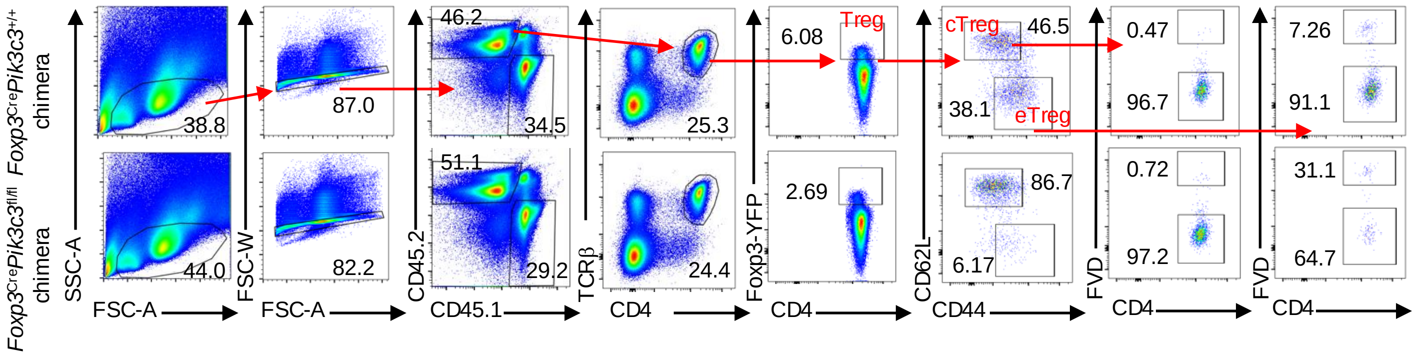

F. Gating strategy for Fig 2D:

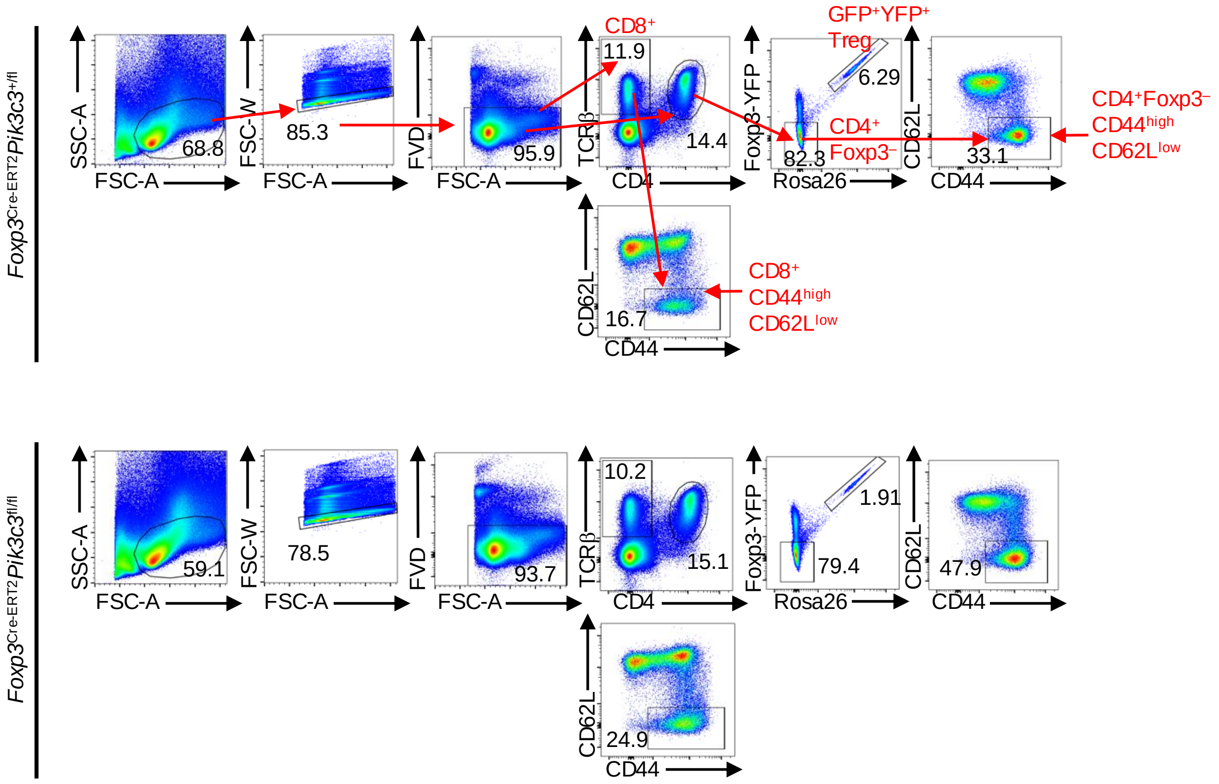

G. Gating strategy for Fig 2E:

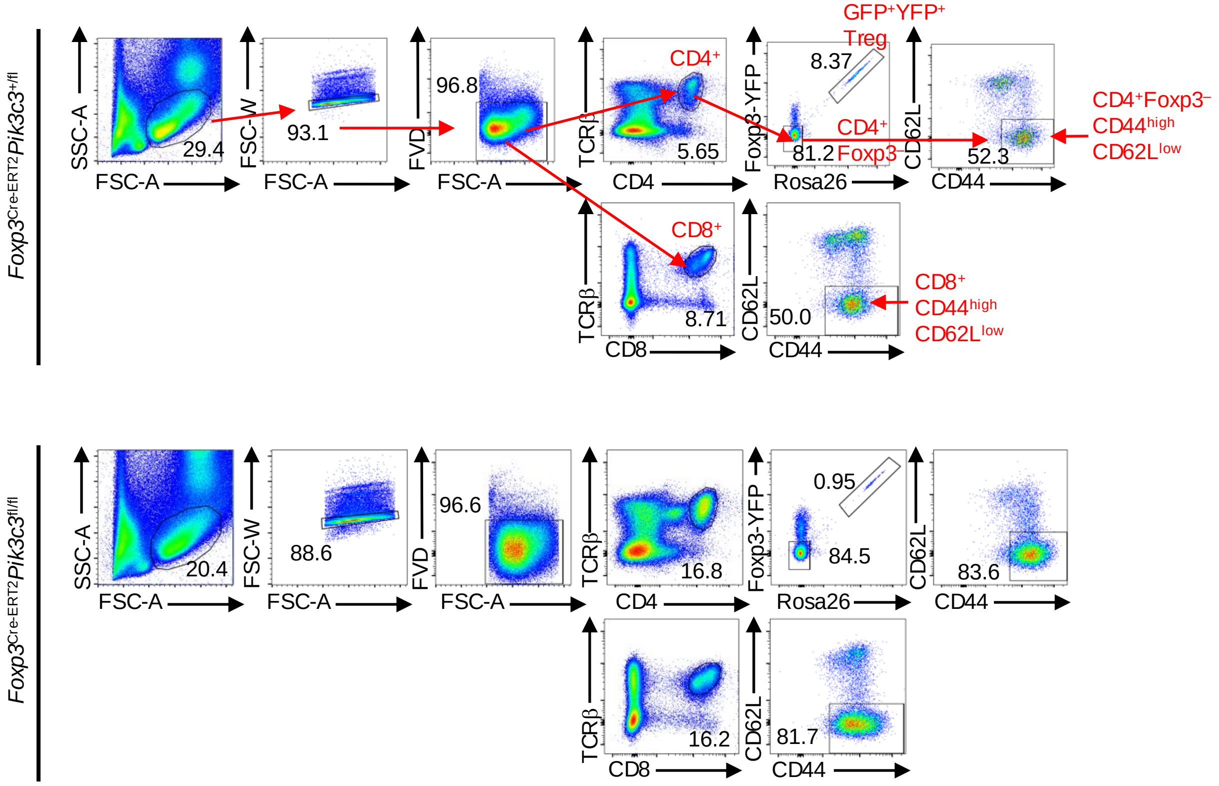

H. Gating strategy for Fig 2H (TIGIT and CTLA-4)

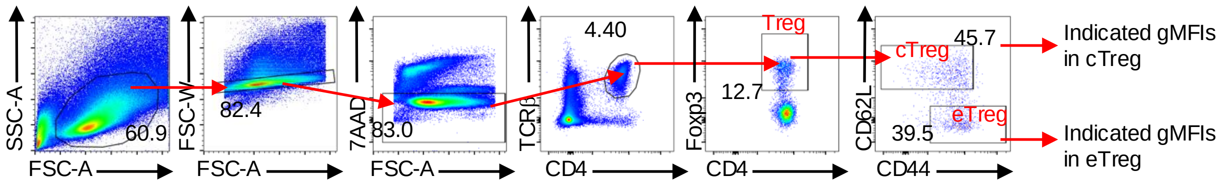

I. Gating strategy for Fig 3G:

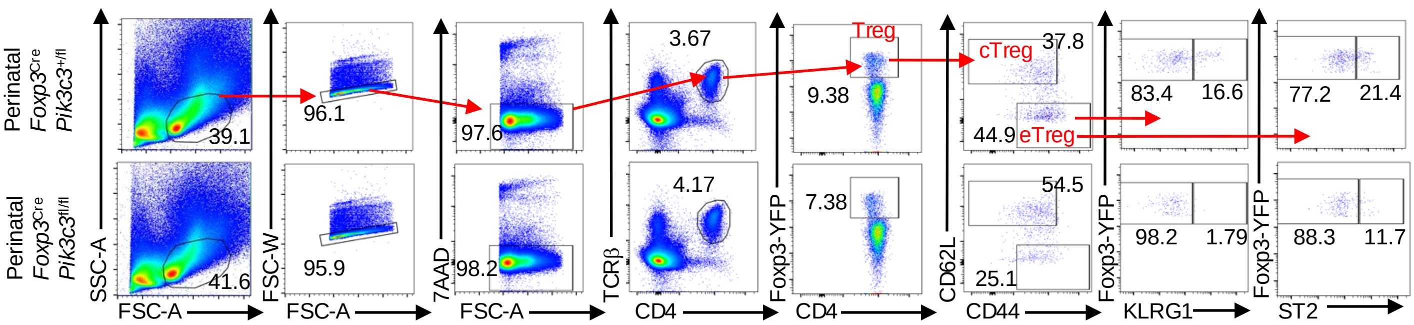

J. Gating strategy for Fig 3I:

Spleen

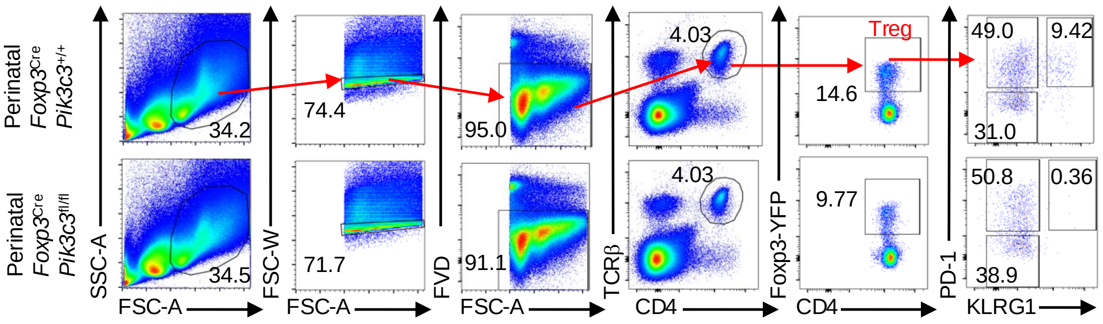

Lung

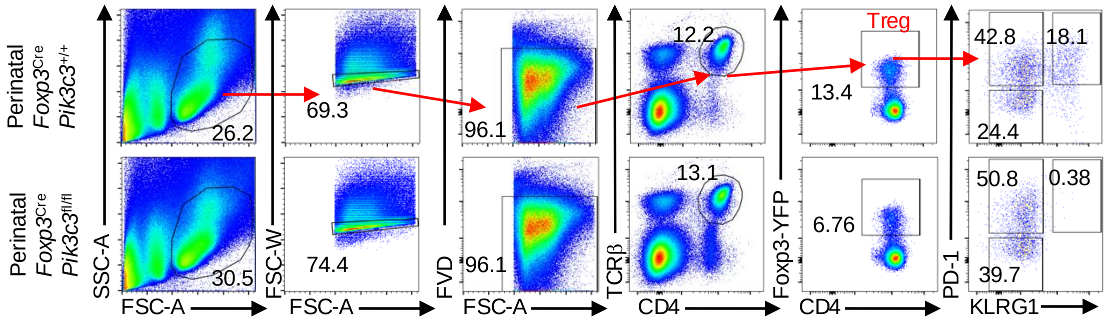

K. Gating strategy for Figs. 4D and S4B:

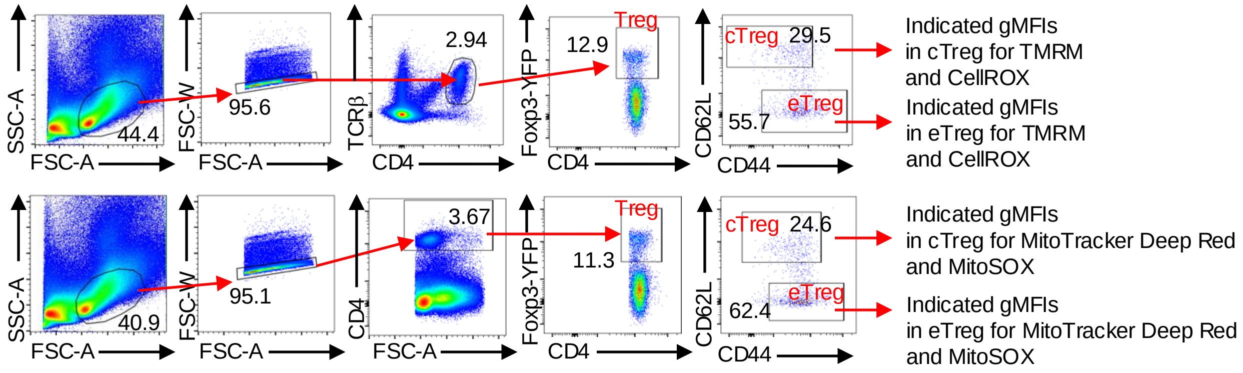

L. Gating strategy for Fig 5C:

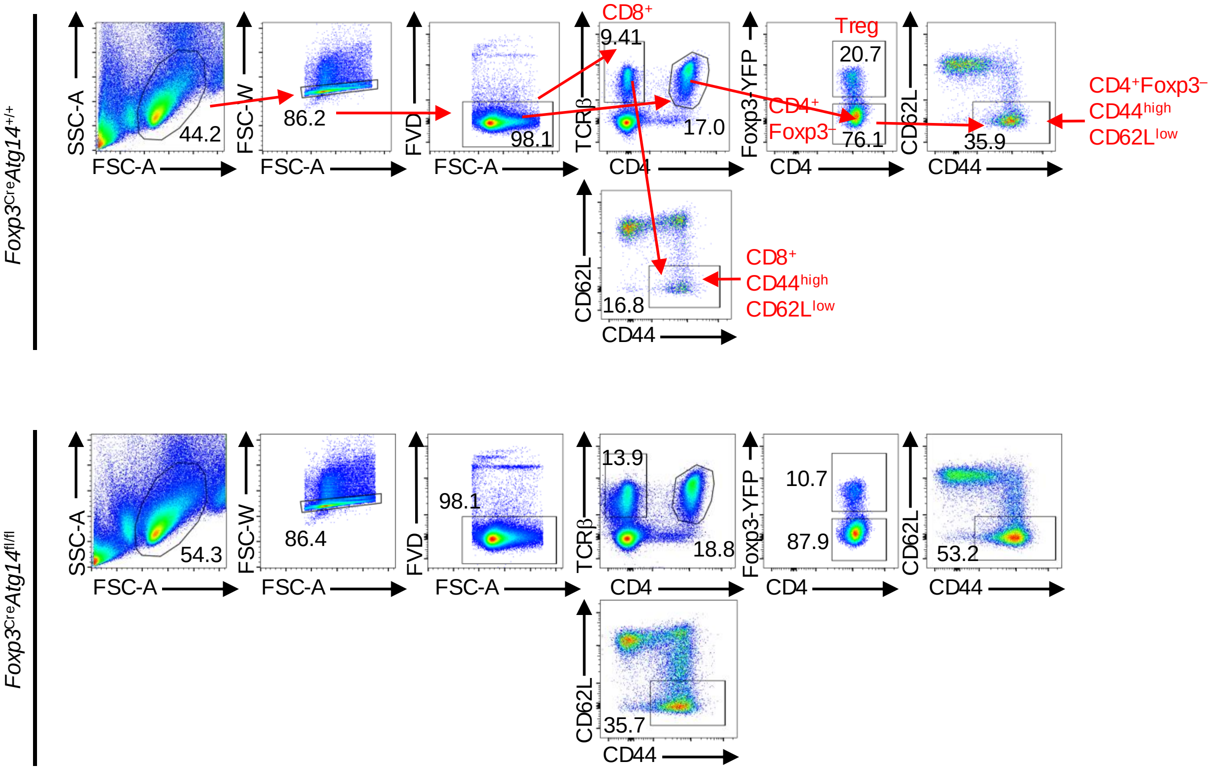

M. Gating strategy for Fig 5D:

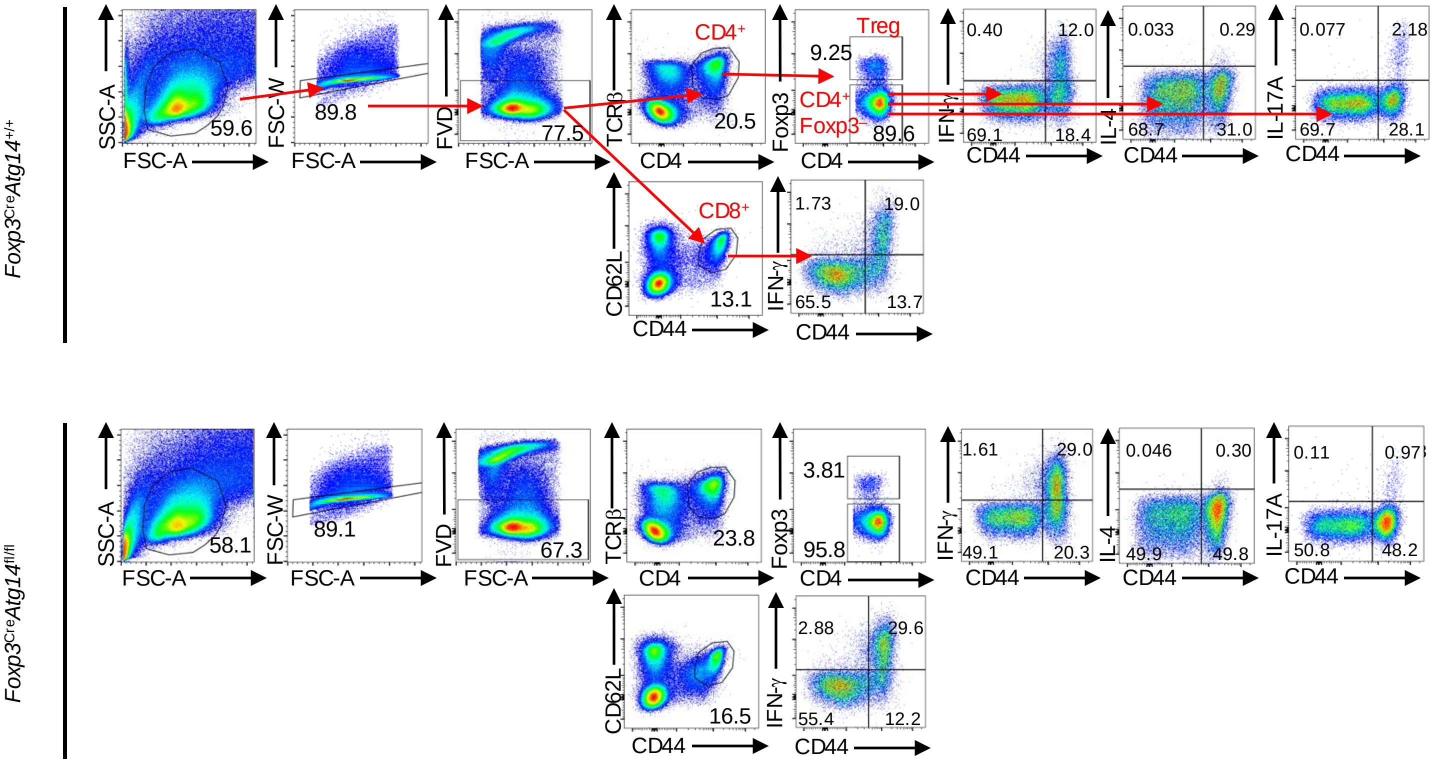

N. Gating strategy for Fig 5E and 5F:

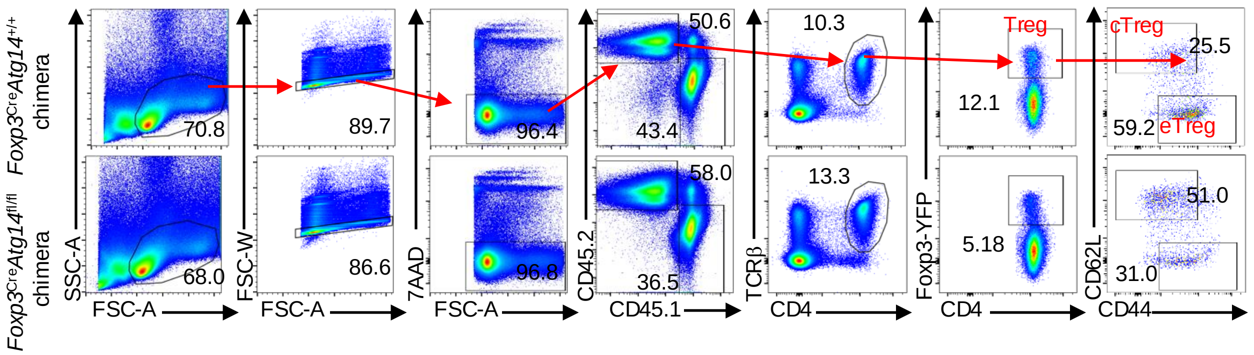

O. Gating strategy for Fig 6C and 6E:

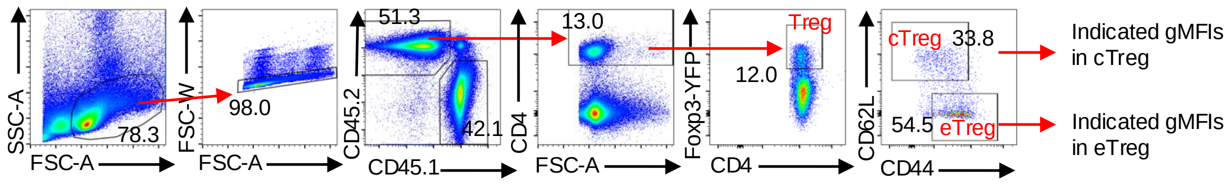

P. Gating strategy for Fig 6J:

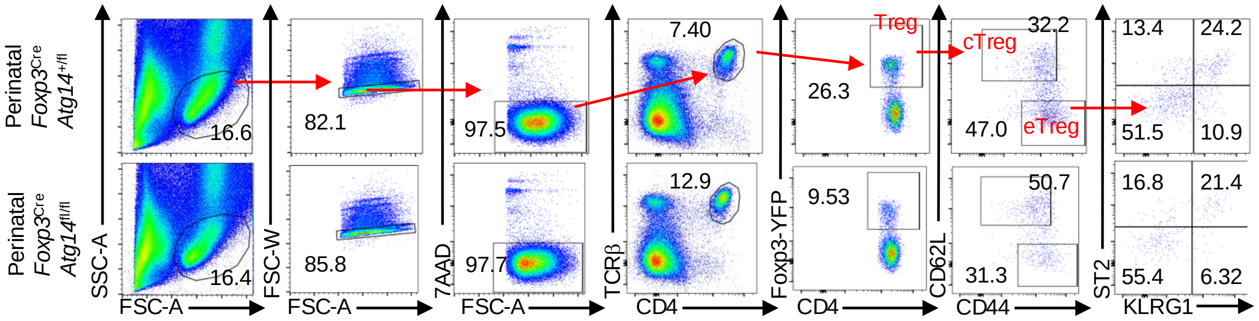

Q. Gating strategy for Fig S1C:

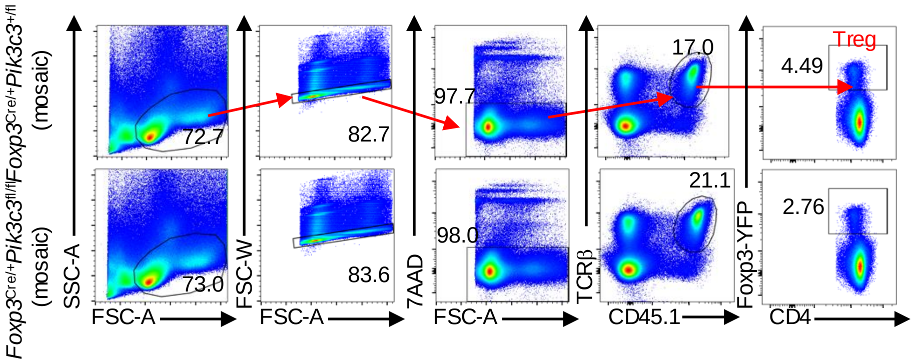

R. Gating strategy for Figs. S1H (Foxp3), S1I, and S2H (TIGIT, CTLA-4):

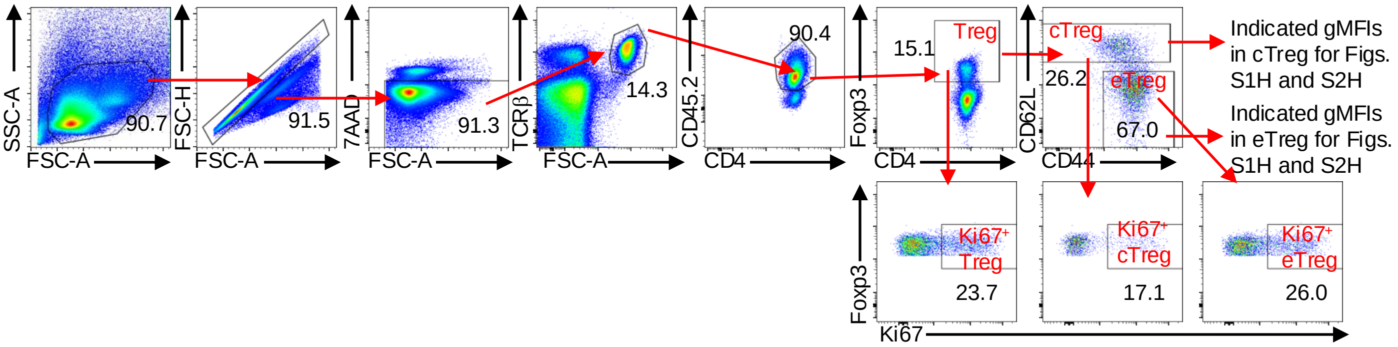

S. Gating strategy for Fig S2B:

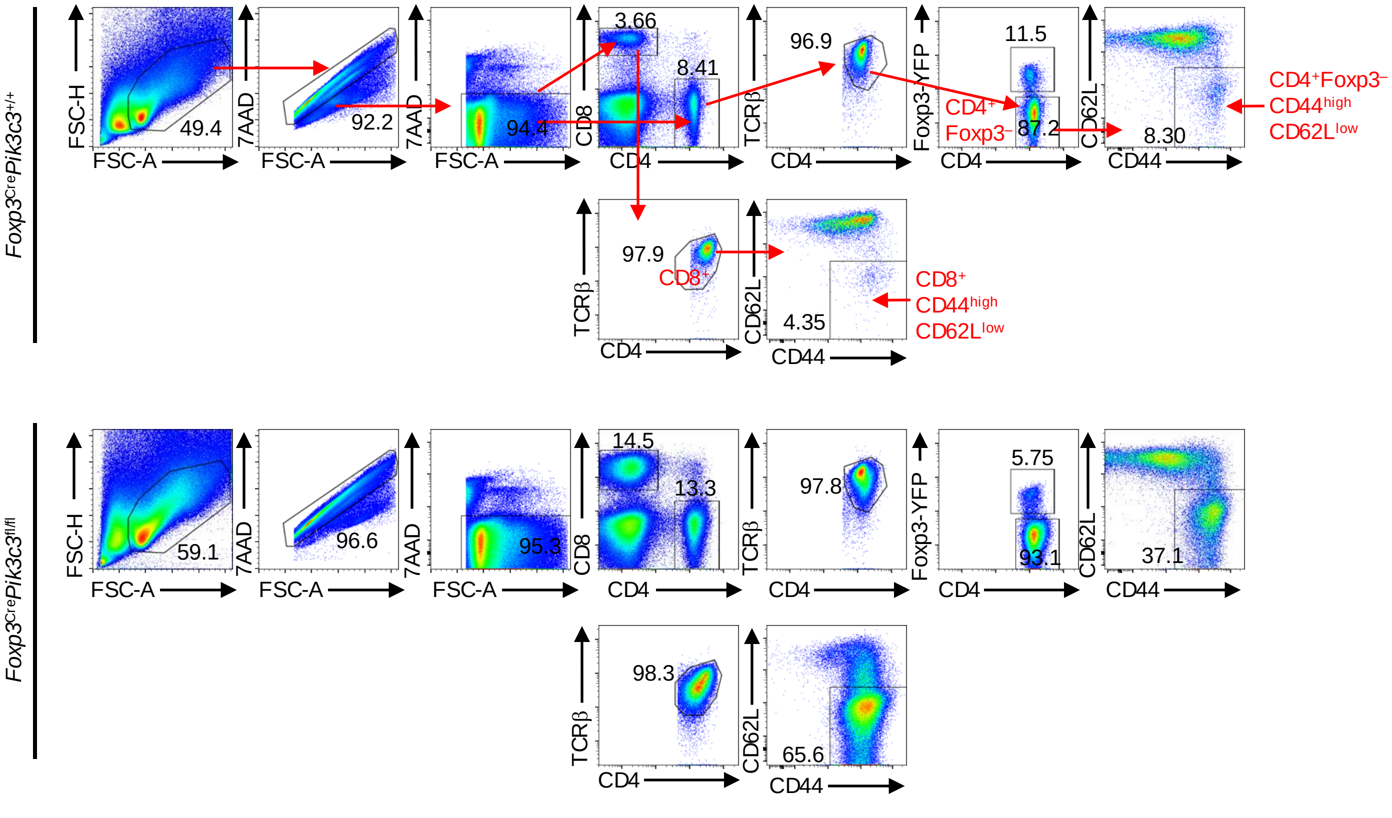

T. Gating strategy for Fig S2C:

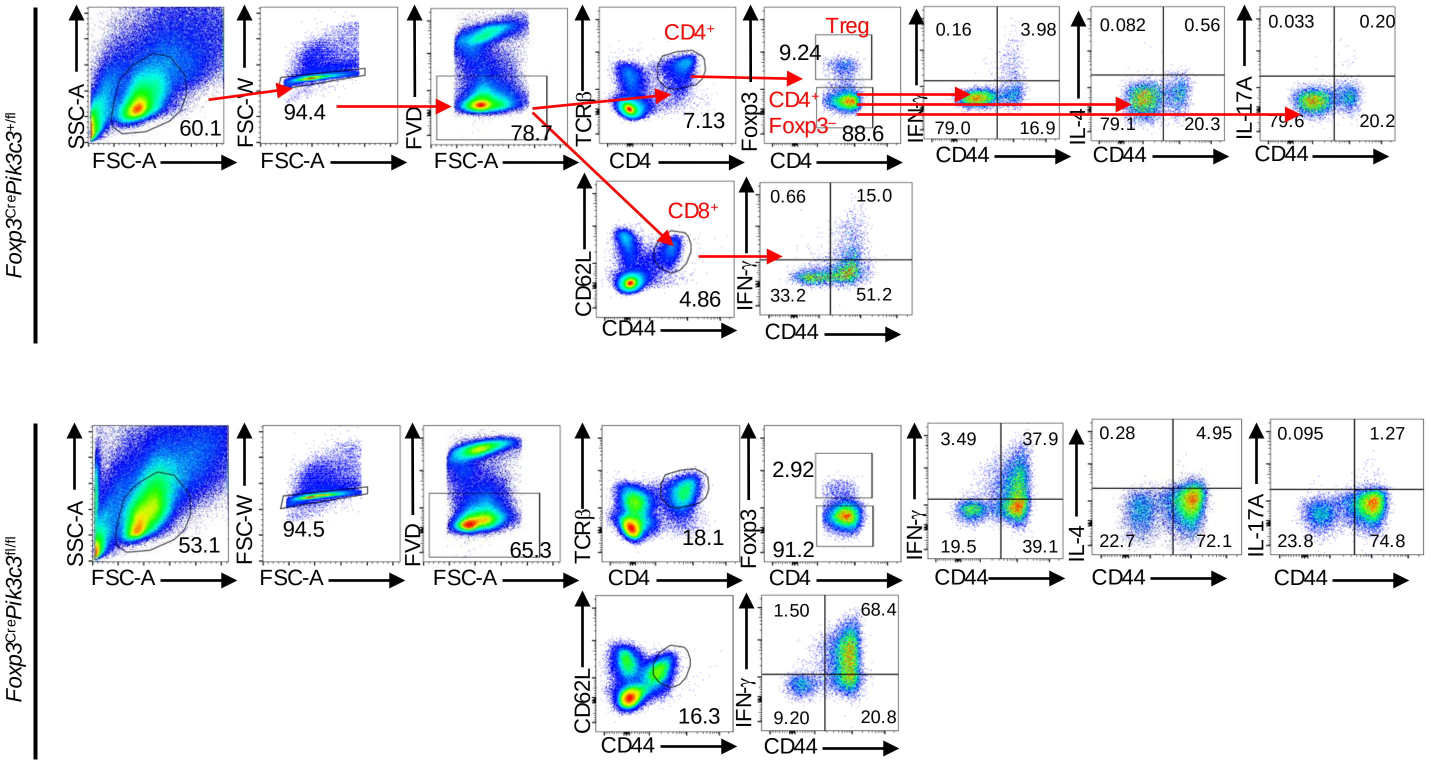

U. Gating strategy for Fig S2G:

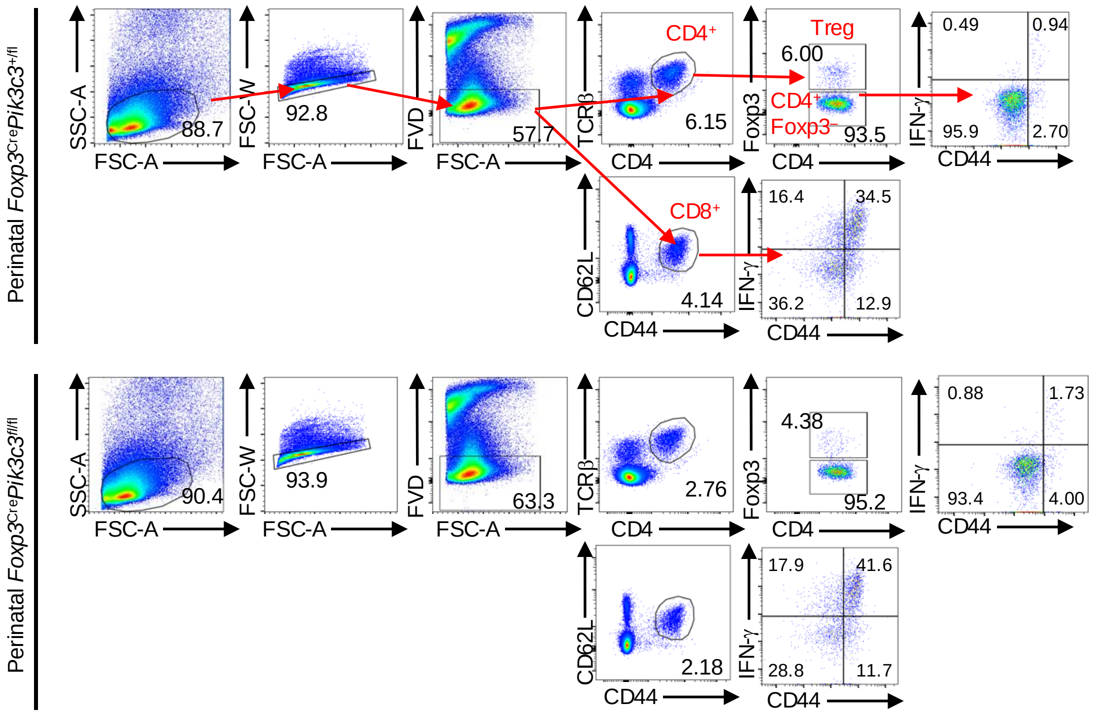

V. Gating strategy for Fig S4A:

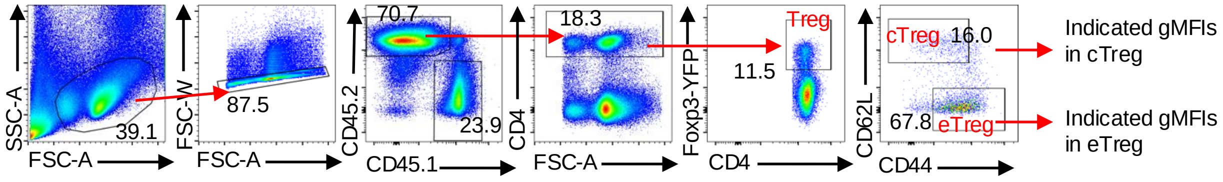

W. Gating strategy for Fig S5D:

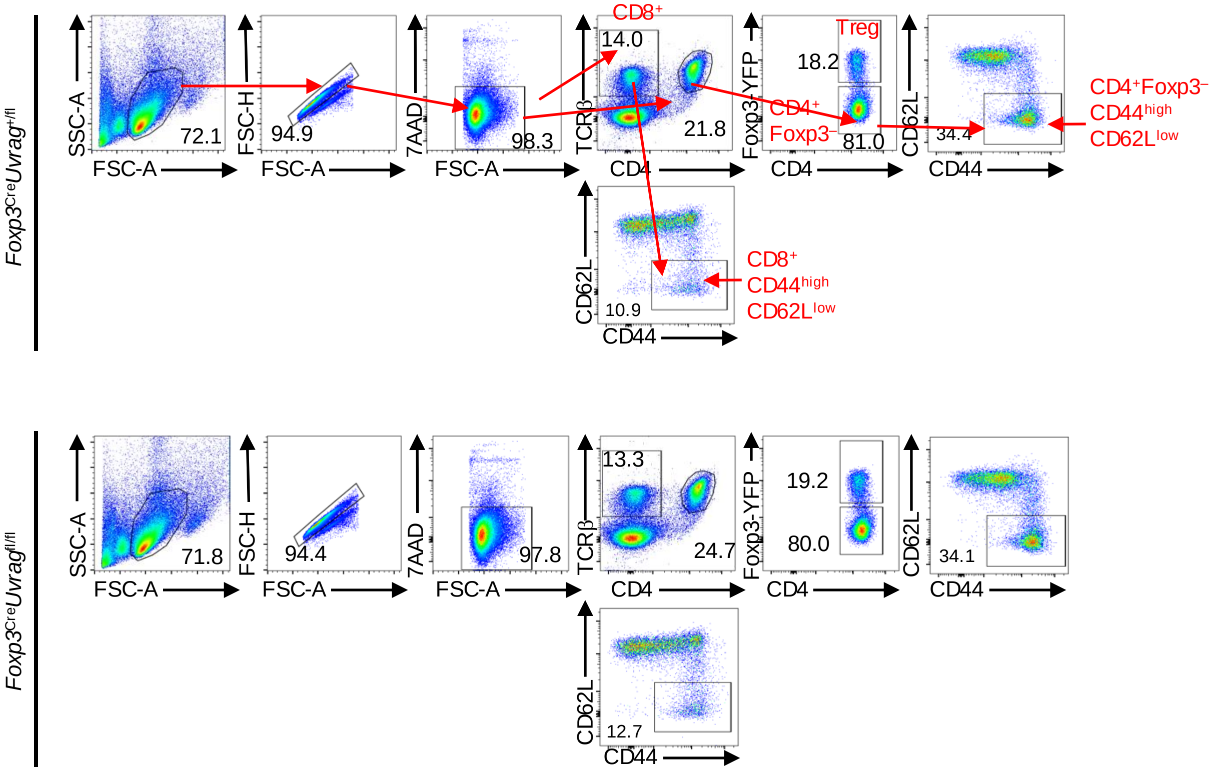

X. Gating strategy for Fig S5H and S5I:

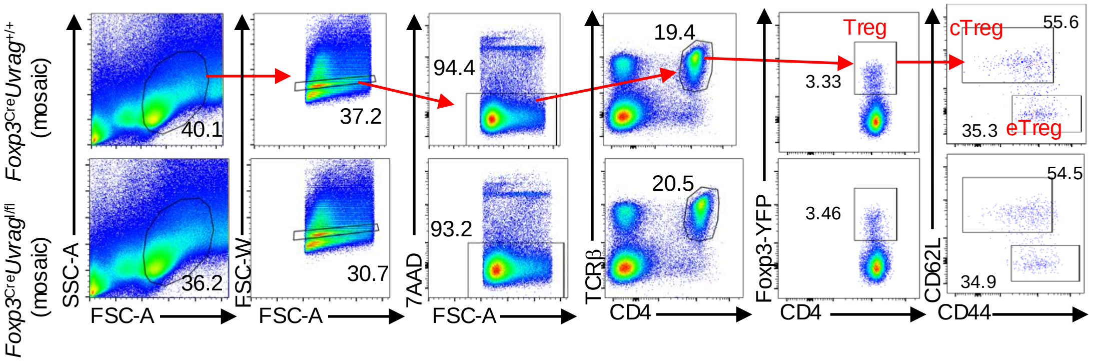

Supplement: S1 File — (PDF) [file pbio.3003074.s008.pdf]
